# Organellar proteomics reveals hundreds of novel nuclear proteins in the malaria parasite *Plasmodium falciparum*

**DOI:** 10.1186/gb-2012-13-11-r108

**Published:** 2012-11-26

**Authors:** Sophie C Oehring, Ben J Woodcroft, Suzette Moes, Johanna Wetzel, Olivier Dietz, Andreas Pulfer, Chaitali Dekiwadia, Pascal Maeser, Christian Flueck, Kathrin Witmer, Nicolas MB Brancucci, Igor Niederwieser, Paul Jenoe, Stuart A Ralph, Till S Voss

**Affiliations:** 1 Department of Medical Parasitology and Infection Biology, Swiss Tropical and Public Health Institute, Socinstrasse 57, Basel 4051, Switzerland; 2 University of Basel, Petersplatz 1, Basel 4003, Switzerland; 3 Department of Biochemistry and Molecular Biology, Bio21 Molecular Science and Biotechnology Institute, The University of Melbourne, 30 Flemington Road, Parkville 3010, Australia; 4 Biozentrum, University of Basel, Klingelbergstrasse 50/70, Basel 4056, Switzerland

**Keywords:** Malaria, *Plasmodium falciparum*, Nucleus, Proteomics, Bioinformatics, IFA, Transcription, Nucleolus, Nuclear pore, Transfection

## Abstract

**Background:**

The post-genomic era of malaria research provided unprecedented insights into the biology of *Plasmodium *parasites. Due to the large evolutionary distance to model eukaryotes, however, we lack a profound understanding of many processes in *Plasmodium *biology. One example is the cell nucleus, which controls the parasite genome in a development- and cell cycle-specific manner through mostly unknown mechanisms. To study this important organelle in detail, we conducted an integrative analysis of the *P. falciparum *nuclear proteome.

**Results:**

We combined high accuracy mass spectrometry and bioinformatic approaches to present for the first time an experimentally determined core nuclear proteome for *P. falciparum*. Besides a large number of factors implicated in known nuclear processes, one-third of all detected proteins carry no functional annotation, including many phylum- or genus-specific factors. Importantly, extensive experimental validation using 30 transgenic cell lines confirmed the high specificity of this inventory, and revealed distinct nuclear localization patterns of hitherto uncharacterized proteins. Further, our detailed analysis identified novel protein domains potentially implicated in gene transcription pathways, and sheds important new light on nuclear compartments and processes including regulatory complexes, the nucleolus, nuclear pores, and nuclear import pathways.

**Conclusion:**

Our study provides comprehensive new insight into the biology of the *Plasmodium *nucleus and will serve as an important platform for dissecting general and parasite-specific nuclear processes in malaria parasites. Moreover, as the first nuclear proteome characterized in any protist organism, it will provide an important resource for studying evolutionary aspects of nuclear biology.

## Background

As one of the most deadly infectious diseases in the world, malaria causes close to 500 million clinical cases and 1 million deaths every year [1,2]. Most of this burden is due to infections with *Plasmodium falciparum*, one of six *Plasmodium *species known to elicit malaria in humans [3,4]. Malaria-related morbidity and mortality is exclusively associated with the erythrocytic stage of infection where repeated rounds of intracellular parasite development and re-invasion into red blood cells (RBCs) lead to exponential parasite proliferation. The entire parasite life cycle is much more complex involving several morphologically and functionally distinct extra- and intracellular stages, and obligate transmission between two hosts, female *Anopheles *spp. and humans.

The key to this amazing biological complexity lies within the parasite nucleus that, in the case of *P. falciparum*, encloses and regulates a 23Mb genome encoding 5,400 genes on 14 linear chromosomes [5]. However, albeit many nuclear processes such as transcription, splicing, DNA replication/repair, mitosis, and the temporal and spatial organization of the nucleus have been studied in detail in model eukaryotes our understanding of nuclear biology in *P. falciparum *is very limited. This is not surprising given that >50% of all genes code for proteins with no known or even inferred function [5-7]. While many seminal studies in the post-genomic era of malaria research provided unprecedented insights into the biology of *P. falciparum*, they also highlighted our profound lack of understanding of basic biological processes in this parasite. In light of spreading drug resistance and the eager expectation for an effective vaccine, acquisition of such knowledge is urgently needed.

During the pre-replicative phase of the intra-erythrocytic developmental cycle (IDC), parasites develop into morphologically distinct ring and trophozoite stages. Schizogony is characterized by multiple rounds of genome replication and closed mitosis before cytokinesis produces new daughter merozoites from multinucleated schizonts [8,9]. At the ultrastructural level, the parasite nucleus appears spherical and contains a mixture of electron-sparse and electron-dense material probably reflecting euchromatic and heterochromatic zones, respectively. The distribution of this material appears to be sensitive to its precise fixation and preparation [10-12].

Several high-throughput transcriptome and proteome profiling studies revealed that in addition to a core set of genes expressed in multiple/all life cycle stages, a large number of genes are specifically expressed in only a single stage, many of which are involved in highly specialized processes and pathways [13-19]. Differential gene expression is also strikingly observed during the 48-h IDC. Detailed microarray experiments performed at high temporal resolution identified a surprisingly structured cascade of gene transcription during this stage [20-22]. About 80% of the genes expressed during the IDC display temporal variation in transcript abundance where genes appear to be activated only when their encoded protein functions are required [21]. Notably, the timely expression of variant protein families involved in immune evasion and RBC invasion is directly related to the pronounced virulence of *P. falciparum *[23]. However, despite the fact that many of these processes are likely governed by transcriptional control little detail on the underlying mechanisms has yet been elucidated.

General transcription factors (TFs) and chromatin remodelling activities are well conserved in the *P. falciparum *proteome [24-27]. Several factors of the latter class have recently been localized to different subcompartments within the parasite nucleus [28]. Functional studies identified important roles for the histone deacetylase PfGCN5, silent information regulator 2 (PfSIR2) and heterochromatin protein 1 (PfHP1) in parasite development, heterochromatin formation, and virulence gene expression [29-37]. In contrast, however, the striking under-representation of identified sequence-specific TFs in the *P. falciparum *proteome compared to those of fungi, plants, and metazoans has hampered targeted research to understand gene-specific control [24,38]. Until recently, only a single TF, PfMYB1, had been analyzed to any extent *in vivo *[39]. Fortunately, the discovery of the apicomplexan-specific ApiAP2 family of DNA-binding factors and functional analysis of some members sparked new interest in this field [40-46].

Most proteomic studies on *Plasmodium *parasites have focused on elucidating whole cell proteomes, which generated valuable insight into the overall structure of, and differences between, the active proteomes in different parasite life cycle stages [13,14,16,17,47], or in response to perturbations such as drug treatment [48-51]. However, while these approaches typically detect large numbers of different proteins they fail to provide information on subcellular protein localization. Organellar proteomics is an important tool to gain new insight into cellular structures and functions since proteins localizing to distinct subcellular compartments are usually associated with the function of these compartments. To date, only a few mass spectrometry-based studies aimed at identifying protein compositions of *Plasmodium *cellular compartments. These include the analysis of fractions enriched for *P. falciparum *food vacuoles (116 proteins) [52], Maurer's clefts (78 proteins) [53], and the infected RBC membrane (36 proteins) [54]. Rodent *Plasmodium *spp. have also been analyzed, searching for rhoptry (36 proteins) [55] and micronemal proteins (345 proteins) [56].

Here, we performed a detailed proteomic analysis of *P. falciparum *nuclei during intra-erythrocytic development. Our approach combined multidimensional protein identification technology (MudPIT) of purified and fractionated nuclei with validation by both bioinformatic appraisal and *in-vivo *localization experiments. We present a robust core nuclear proteome consisting of 802 proteins. Our comprehensive analysis of this inventory provides unprecedented insight into the parasite nucleus and will be of great benefit to future studies investigating nuclear biology in this important pathogen.

## Results

### Isolation and fractionation of parasite nuclei

**Figure 1:**
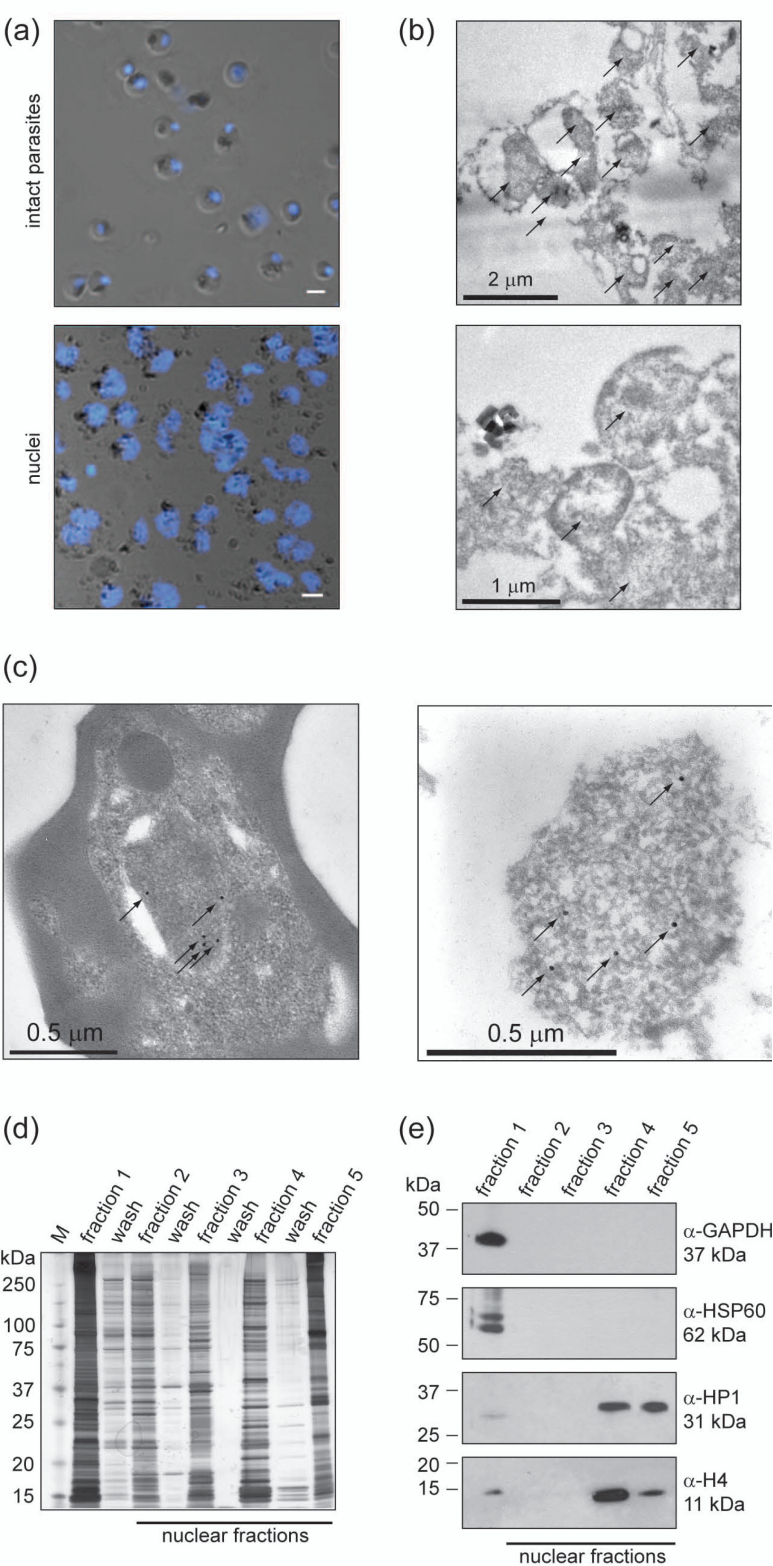
**Preparation of crude *P. falciparum *nuclei for proteomic analysis**. **(a) **Visual assessment of the crude nuclear preparation. Intact parasites (control) and isolated nuclei were stained with DAPI and analyzed by DIC and fluorescence microscopy. Scale bar 2 μm. **(b) **TEM analysis of isolated nuclei shows intact as well as damaged nuclei and very little contamination from other organelles. Arrows indicate individual nuclei. **(c) **Immunoelectron microscopy verifies the identity of nuclei. Anti-H3K4me3 antibodies were used to label nuclei in intact cells (left panel) and in the nuclear preparation (right panel) (arrows). **(d) **1D-SDS PAGE analysis of all protein fractions obtained from trophozoites, visualized by silver staining. **(e) **Analysis of trophozoite cytoplasmic and nuclear fractions by western blot.

To obtain a broad overview of proteins localized to the parasite nucleus we identified the protein content of crude nuclear preparations followed by further biochemical fractionation from ring, trophozoite, and schizont stage parasites by high accuracy mass spectrometry (Additional file 1). Isolated nuclei were significantly increased in size compared to intact parasites, a phenomenon regularly seen in preparations of nuclei after hypotonic lysis [57,58] (Figure 1a). Despite extensive washing of the nuclear pellet, contamination with free hemozoin crystals was obvious. Analysis by transmission electron microscopy (TEM) showed that the nuclear fraction mainly consisted of rounded and enlarged nuclei with varying degrees of intactness (Figure 1b). No consistent organellar impurities were apparent, although some haemozoin crystals were also visible throughout these samples. Importantly, immunolabeling with antibodies specific for histone 3 lysine 4 tri-methylation (H3K4me3) identified these structures as parasite nuclear material (Figure 1c).

We next performed protein fractionation to reduce sample complexity. Nuclei were serially extracted with 0.1 M KCl (fraction 2), DNAseI (fraction 3), 1 M KCl (fraction 4), and 2% SDS (fraction 5). Each fraction displayed a distinct 1D-SDS-PAGE protein pattern indicative of differential protein extraction (Figure 1dand Additional file 1). The cytosolic enzyme glyeraldehyde-3-phosphate dehydrogenase (GAPDH) and the mitochondrial heat shock protein 60 (HSP60) were exclusively detected in the NP40-soluble cytoplasmic extract (fraction 1) demonstrating efficient lysis of the parasite plasma membrane and the double membrane-bound mitochondrion (Figure 1e). In contrast, H4 and PfHP1 were soluble only after extraction of nuclear pellets with high salt and SDS, consistent with their intimate association with chromatin. In summary, the microscopy-, SDS-PAGE-, and immunodetection-based assessments show that the protocol applied here efficiently separated the cytoplasmic and nuclear compartments and yielded distinct subnuclear protein fractions suitable for mass spectrometry analysis.

### Nuclear proteome determination by MudPIT

**Figure 2:**
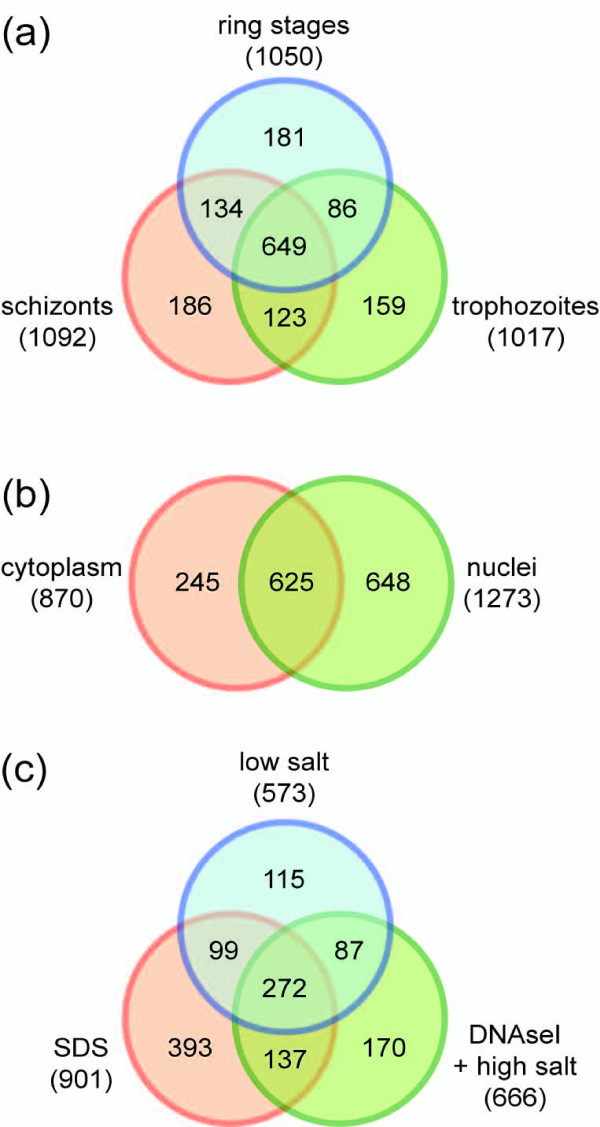
**Distribution of the 1,518 detected proteins in developmental stages, subcellular compartments, and nuclear fractions**. Detection of proteins in the various pools is depicted by Venn diagrams. Absolute numbers of proteins in each pool are indicated in brackets. **(a) **Protein distribution in ring stages, trophozoites, and schizonts. **(b) **Protein distribution in the cytoplasmic versus the combined nuclear fractions. **(c) **Protein distribution across the four nuclear fractions (DNAseI and high salt fractions were combined).

Endoproteinase LysC- and trypsin-digested total proteins were analyzed by two-dimensional capillary liquid chromatography and tandem mass spectrometry (MudPIT). MS/MS spectra were searched against a combined *P. falciparum/*human proteome database using the SEQUEST algorithm [59]. We identified 1,518 different parasite proteins that were represented by at least one peptide in any of the 30 samples analyzed (fractions 1 to 5 each for ring stages, trophozoites, schizonts; two biological replicate samples each) (Additional file 2). Similar numbers of proteins were identified for ring stages (1,050), trophozoites (1,017), and schizonts (1,092). A total of 649 proteins were shared by all three stages, and similar numbers of proteins were either unique to one stage or shared between any two of the three stages (Figure 2a). In the cytoplasmic and combined nuclear fractions 870 and 1,273 proteins were detected, respectively, and 625 were shared between both compartments (Figure 2b). Figure 2c shows the distribution of proteins in individual nuclear fractions (DNAseI- and high salt-soluble fractions were combined into one category potentially enriched in DNA/chromatin-associated proteins). The percentages of proteins measured in the low salt, DNAseI/high salt and SDS fractions were 20.1%, 25.5% and 43.6%, respectively, and were unique to the corresponding fraction.

On average, 69.7% (+/- 14.7 SD) of proteins measured in a given sample were also detected in its matching replicate. Furthermore, calculation of Spearman rank coefficients showed that each sample correlated best with its matching replicate (Additional file 2). This clearly underlines the repeatability of the fractionation and the mass spectrometric methods applied. The absolute correlations between replicates were moderate (0.50 +/- 0.09 SD; Spearman rank coefficients). This can be explained by the stochastic sampling of peptides in the mass spectrometer, a limitation inherently associated with shotgun proteomics of complex samples, and by biological and experimental sample-to-sample variation. Undersampling is a consequence of the limited speed with which MS/MS spectra can be acquired and is specific for the instrument used. Both factors contribute to the differential detection/identification of proteins, particularly for low abundance proteins.

To maximally cover the nuclear proteome, we accepted protein identifications based on single peptide matches. For reliable peptide identifications we used the decoy search strategy querying a reversed sequence database [60]. This strategy resulted in very low mean false discovery rates of 0.0028 +/- 0.0024 SD per sample, reflecting the stringent search criteria applied for peptide identification and acceptance of single peptide hits (Additional files 1 and 2). Furthermore, random inspection of single peptide-based protein identifications confirmed the automated protein identification of the search engine. In light of these high confidence identifications, proteins represented by single peptides were included in all downstream analyses. The SEQUEST output data for all stages and fractions (Additional files 3, 4, and 5) and a comprehensive summary table (Additional file 6) are provided as supplementary information.

### The preparation of isolated nuclei is enriched in nuclear proteins

Functional enrichment analyses using the David and the GOstat tools [61,62] revealed that in all cases proteins detected in the combined nuclear fractions only were statistically enriched in annotations consistent with nuclear functions, whereas proteins found in the cytoplasmic fraction only were enriched in functions known to be cytosolic (Additional files 1 and 7). As shown in Table 1, all annotated histones were detected and their distribution was marked by a clear enrichment in the nuclear fractions. We measured 10 out of 12 subunits of the DNA-directed RNA polymerase II (RNApolII) complex, all of which were detected in fraction 4. Peptides derived from ApiAP2 factors (13 out of 27 detected) were exclusively present in nuclear fractions. Furthermore, we detected 111 out of 247 *in silico *predicted *P. falciparum *transcription-associated proteins (TAPs) [38,63], 108 of which (97.3%) were present in any of the four nuclear fractions (Additional file 6).

**Table 1:** Distribution of peptides derived from a representative selection of known nuclear proteins across the three IDC stages.

Protein ID	Product description	Fraction 1			Fraction 2			Fraction 3			Fraction 4			Fraction 5		
		**R**	**T**	**S**	**R**	**T**	**S**	**R**	**T**	**S**	**R**	**T**	**S**	**R**	**T**	**S**

	**Histones**															
PFF0860c	histone H2A	1	1	1	7	1	3	12	3	5	7	4	8	1	1	3
PFC0920w	histone H2A.Z				2		2	9	4	6	8	3	5	4	3	4
PF11_0062	histone H2B	2	1		7	2	9	36	7	10	12	14	22	9	7	12
PF07_0054	histone H2B variant	2	1		1		3	17	9	4	12	7	15	8	5	7
PFF0510w	histone H3				1			4			8	4	7	2		
PFF0865w	histone H3 variant							1					3			
PF13_0185	histone H3, CENP-A variant										3		2			
PF11_0061	histone H4	1	3	2	3	1	7	10	3	4	15	13	22	8	6	13
	**DNA-directed RNA polymerase II complex**															
PFC0805w	DNA-directed RNA pol II, putative	4									8	31	5	4	4	
PFI1130c	DNA-directed RNA pol II, putative										1	6	2			
PF10_0269	DNA-directed RNA pol II, putative								1			2				
PFB0715w	DNA-directed RNA pol II 2nd largest subunit, putative										1	18	4		2	
PFL0665c	RNA polymerase subunit 8c, putative											1				
PFB0245c	DNA-directed RNA pol II 16 kDa subunit, putative											2				
PF13_0341	DNA-directed RNA pol II, putative											3	1			
PFA0505c	DNA-directed RNA pol II, putative										1	1				
PF13_0023	DNA-directed RNA pol II, putative											3	2			
PF07_0027	DNA-directed RNA pol II 8.2 kDa polypeptide, putative											1				
	**ApiAP2 transcription factors**															
PFF0200c	PfSIP2									1						7
PFE0840c	transcription factor with AP2 domain(s), putative					3									1	
PFD0985w	transcription factor with AP2 domain(s), putative													1	1	
PF11_0404	transcription factor with AP2 domain(s), putative								1						6	
PF10_0075	transcription factor with AP2 domain(s), putative				3	9				4		3		2	8	10
PF14_0471	transcription factor with AP2 domain(s), putative					4								1	4	5
PF14_0633	transcription factor with AP2 domain(s), putative					1			2	2				2	6	6
PF14_0533	transcription factor with AP2 domain(s), putative														1	3
PF11_0091	transcription factor with AP2 domain(s), putative				2	9	1			2					1	5
PFL1900w	transcription factor with AP2 domain(s), putative					29			2						14	21
PFF0670w	transcription factor with AP2 domain(s), putative									4				2	3	35
PF14_0079	transcription factor with AP2 domain(s), putative														1	
MAL8P1.153	transcription factor with AP2 domain(s), putative															4

Indicated are the numbers of unique tryptic peptides measured for each protein in each fraction and IDC stage, derived from either replicate A or B, whichever displayed the higher number of unique peptides. R, ring stages; S, schizonts; T, trophozoites;.

**Figure 3:**
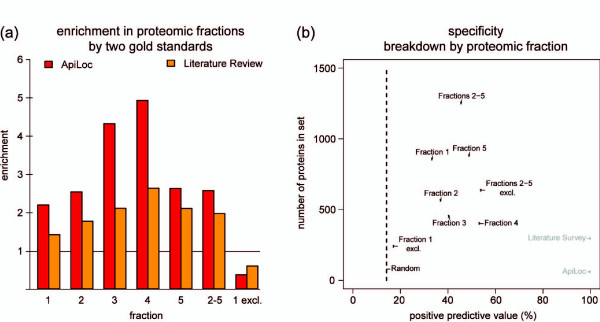
**Enrichment of nuclear proteins in nuclear fractions by comparison to gold standards**. **(a) **Enrichment was defined as the percentage of true nuclear proteins found in each fraction, divided by the number of nuclear proteins expected in a random set of equivalent size. The horizontal line indicates the theoretical enrichment in a random selection of proteins. **(b) **Nuclear protein content in various protein fractions. The vertical dashed line indicates the PPV for proteins taken at random from the entire parasite proteome. 1-5, fractions 1 to 5; 2-5, all proteins found in any of the four nuclear fractions; 1 excl., proteins found exclusively in fraction 1; 2-5 excl., all proteins found exclusively in any of the four nuclear fractions.

To further assess the enrichment in nuclear proteins, we carried out comparisons to curated lists of proteins with known or likely nuclear localization. First, lists were derived from ApiLoc, a database of apicomplexan proteins previously localized by microscopy [64]. At the time of analysis, it held information on 424 *P. falciparum *proteins, 60 of which were nuclear-localized at any stage during the life cycle (14.3%). A simple algorithm, designed to decide whether the annotation recorded in ApiLoc is consistent with a protein being found in the nucleus (Additional file 1), showed that 37 of these 60 nuclear proteins were detected in the nuclear preparation (Additional file 6). Proteins in the cytoplasmic and any of the four nuclear fractions were enriched in nuclear-localized proteins, whereas those detected exclusively in the cytoplasmic fraction were depleted (one nuclear protein detected) (Figure 3a). These enrichments were statistically significant under the assumption that the proteins with recorded localization annotation were representative (*P *value = 1.4 × 10^-7^; Fisher's exact test). Similarly, accuracy was tested by comparison to a curated list of proteins whose localization has been inferred in peer-reviewed articles, largely through their having an experimentally localized predicted orthologue in model organisms such as *Saccharomyces cerevisiae *or *Homo sapiens *(Additional file 8). Of 465 proteins in this list, 296 (63.7%) are linked to nuclear localization, 136 of which were detected in the nuclear preparation (enrichment *P *value = 3.3 × 10^-5^), and only eight were present exclusively in the cytoplasmic fraction (Figure 3a and Additional file 6). In summary, this overall assessment of the proteomic output highlights the enrichment of known and probable nuclear proteins in the nuclear fractions.

Next, we applied two measures, positive predictive value (PPV) and the number of proteins remaining in each set, to assess differences in content between the nuclear fractions. The pool of detected proteins annotated as nuclear in the ApiLoc database [64] and/or by literature review was used as reference set (159 proteins). As expected, the sets of proteins found in individual fractions showed varying PPVs with the least and most predictive observed in the cytoplasmic lysate and high salt nuclear extract, respectively (Figure 3b). The sum of proteins found in the combined nuclear fractions gave the largest set at a high PPV. Considering only those proteins in this set that were not also detected in the cytoplasm increased the PPV by 8% but came at too steep a cost by removing 49% of all proteins including many of nuclear and unknown localization. This shows that the cytoplasmic fraction is not especially deficient in nuclear proteins, consistent with the presence of many nuclear proteins in both compartments. In contrast, proteins found exclusively in the cytoplasmic fraction were markedly deficient in predicted nuclear proteins.

### Application of bioinformatic reduction techniques to generate a high-confidence core nuclear proteome dataset

**Figure 4:**
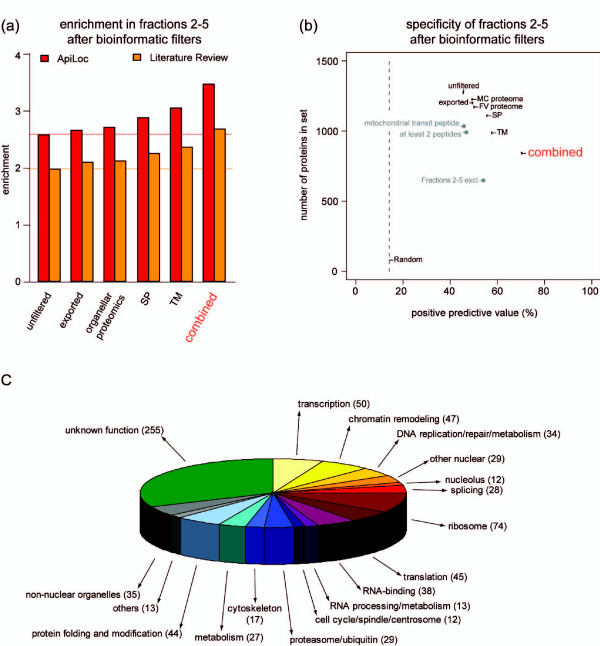
**Bioinformatic filtering of the nuclear proteomics data**. **(a) **Postprocessing enrichments in the set of proteins found in fractions 2 to 5. **(b) **Nuclear protein content in fractions 2 to 5 after bioinformatic filtering. exported, removal of proteins predicted to be exported; organellar proteomics, removal of proteins detected in the Maurer's cleft (MC) [53] and/or the food vacuole (FV) proteome [52]; SP, removal of proteins carrying a predicted SP; TM, removal of proteins carrying predicted TM domains; combined, the five accepted filters (SP, TM, FV, MC, EXP) combined; mitochondrial transit peptides, removal of proteins predicted by PlasMit [67]; at least two peptides, removal of proteins detected by a single peptide only; fractions 2-5 excl., removal of proteins detected in the cytoplasmic fraction. **(c) **Classification of the core nuclear proteome into broad generic functional classes was done by manual inspection of the dataset and is based on functional annotation available on PlasmoDB [68] and literature review. Each protein was assigned to a single class only.

A variety of bioinformatic filters were applied to remove components inconsistent with nuclear localization. Each filter applied was motivated by a hypothesis grounded in current knowledge of the properties of nuclear proteins. We accepted filters to remove proteins: (1) carrying a predicted signal peptide (SP); (2) carrying a predicted transmembrane (TM) domain; (3) predicted to be exported to the RBC based on the presence of a PEXEL motif [65,66] and/or the association of the encoding genes with PfHP1 [35]; and (4,5) found in two previously published *P. falciparum *organellar proteomic studies of Maurer's clefts [53] and the food vacuole [52]. We rejected filters to remove proteins: (1) predicted to localize to the mitochondrion by PlasMit [67]; (2) detected by a single peptide only; and (3) detected in the cytoplasmic fraction. Benchmarked results for each filter are shown in Figure 4, and full rationales for accepting or rejecting filters are available in Additional file 1.

Applying the five filters resulted in a set of 841 proteins with a PPV of 70% (Figure 4b). After removal of 55 proteins experimentally localized to non-nuclear compartments, re-addition of 16 known nuclear proteins, and adjustments due to altered SP annotations (Additional file 9) we present a set of 802 proteins, whereof we estimate that 76% represent true nuclear proteins (compared to 46% in the unfiltered dataset) (Figure 4b). This set is henceforth referred to as the 'core nuclear proteome' and all further analyses were carried out on this set (Additional file 10).

An overview of the contents of the core nuclear proteome is shown in Figure 4c where all proteins are categorized into broad generic classes based on functional annotation. Apart from proteins engaged in typical nuclear processes such as transcription, chromatin remodeling, DNA replication/repair, splicing, and nucleolar functions, the core nuclear proteome contains many putative RNA-binding proteins and factors involved in RNA processing and metabolism, protein folding, modification and degradation, and cytoskeletal organization. Notably, one-third of the core nuclear proteome consists of proteins with no annotated function, and only 5% of all proteins are reliably allocated to non-nuclear compartments.

### Experimental validation by subcellular localization of selected nuclear candidates

To test if the core nuclear proteome will be useful in identifying true nuclear proteins we experimentally validated the subcellular localization of 28 proteins by indirect immuno-fluorescence assays (IFA). We generated transgenic parasite lines expressing full-length C-terminally tagged proteins from episomally maintained plasmids. We chose 22 nuclear protein candidates (NuProCs1-22) that were included in the core nuclear proteome, and six non-nuclear protein candidates (n-NuProCs1-6) that were excluded from the list based on the presence of predicted TM domains and/or SPs (Additional file 11). Of the 22 NuProCs, seven carried annotations indicative for nuclear localization and 15 were annotated as hypothetical proteins at the time of selection on PlasmoDB [68]. Amongst the negative control set, n-NuProC1 (PF11_0099) is annotated as heat shock protein DnaJ homolog Pfj2 whereas all other n-NuProCs are hypothetical proteins with unknown function.

**Figure 5:**
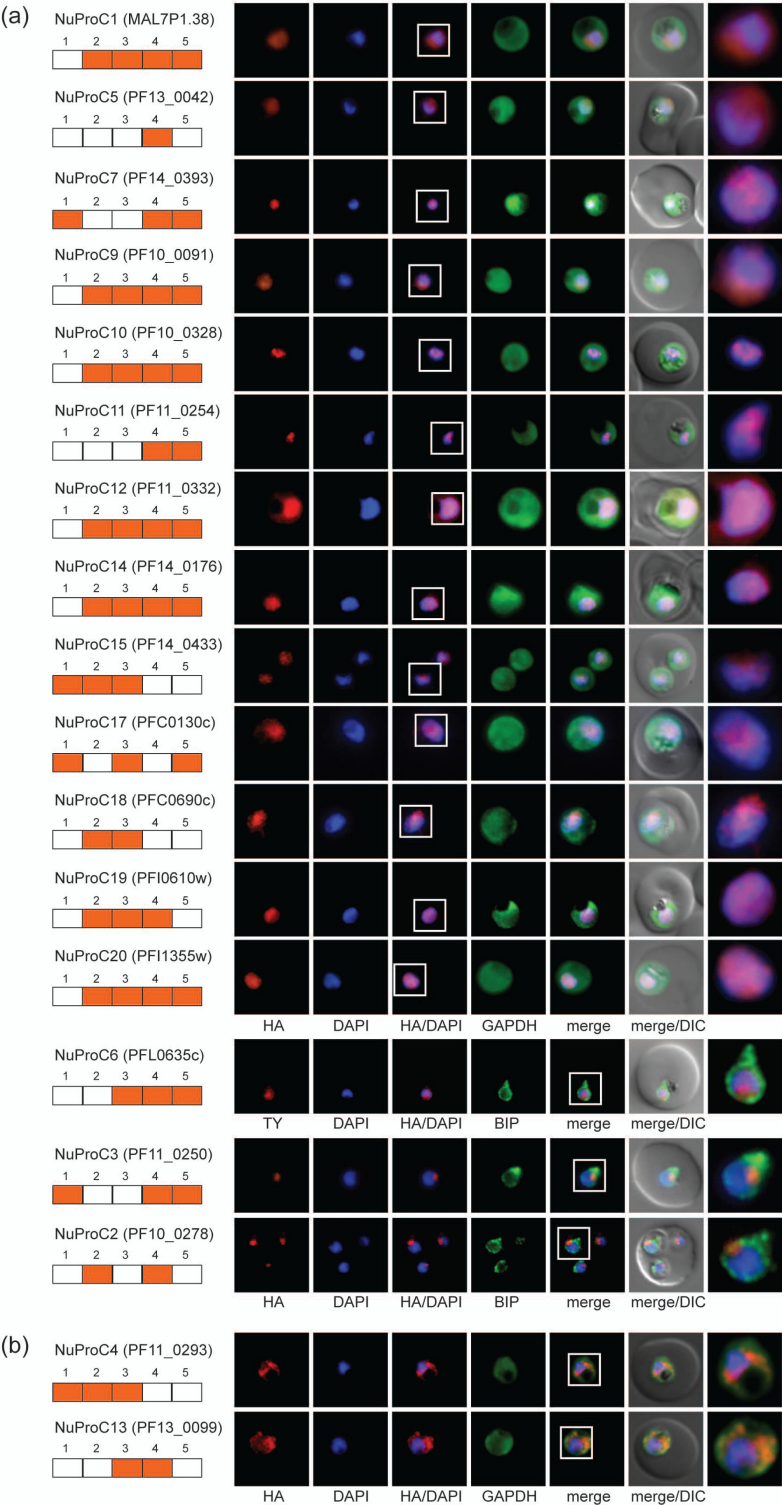
**Experimental validation of the core nuclear proteome by IFA - nuclear localization**. All panels show protein localization in trophozoites. Protein acronym and PlasmoDB gene accession numbers are indicated to the left. Peptide detection or absence in individual fractions is indicated by orange or white boxes, respectively. Epitope-tagged candidates were detected using anti-HA or anti-Ty antibodies (red). The reference compartments cytosol and ER were visualized using antibodies against GAPDH and PfBIP, respectively (green). White frames refer to the magnified view of the nuclear area shown in the rightmost images. **(a) **16 NuProCs co-localize exclusively with the DAPI-stained area of the parasite nucleus. **(b) **NuProC4 and NuProC13 are associated with the nuclear periphery.

Sixteen of the 22 NuProCs co-localized exclusively with the DAPI-stained area of the nucleus in trophozoites (Figure 5a) (detailed IFA localization results throughout the IDC for all NuProC cell lines are available in Additional file 12). Within this group of confirmed nuclear proteins are six of the seven candidates annotated as putative nuclear proteins. A diffuse pattern was observed for NuProC1 (putative regulator of chromosome condensation) and NuProC5 (putative fork head domain protein). NuProC7 (structure-specific recognition protein) displayed a more condensed appearance throughout the IDC. NuProC2 (putative nucleolar preribosomal assembly protein) and NuProC3 (putative splicing factor) both localized to a distinct subnuclear region. NuProC6 encodes a putative bromodomain protein with a more restricted and peripheral localization in ring stages and a diffuse nuclear pattern in trophozoites and schizonts.

Ten candidates annotated as hypothetical proteins (NuProC9, 10, 11, 12, 14, 15, 17-20) were also unambiguously localized within the parasite nucleus. A diffuse pattern was evident for NuProC12, NuProC15, NuProC17, and NuProC20. NuProC18 showed a cytosolic staining in late schizont and ring stages but was clearly nuclear in trophozoites. NuProC9 changed from a rather ubiquitous to a more condensed nuclear localization upon transition from the early to late schizont stage. Both NuProc10 and NuProC11 are localized to unknown nuclear subcompartments. NuProC14 and NuProC19 were preferentially associated with the nuclear periphery in ring stages but showed a diffuse pattern in trophozoite nuclei.

Two additional proteins, NuProC4 (putative multiprotein bridging factor type 1 MBF1) and NuProC13 localized outside but in close proximity to the DAPI signal (Figure 5b). These patterns were clearly distinct from the cytosolic GAPDH signal and reminiscent of that obtained for the nuclear porin PfNUP100 [28] suggesting that these two factors may be associated with the nuclear periphery. In addition, NuProC4 appeared to accumulate in the cytosol in mature trophozoites.

**Figure 6:**
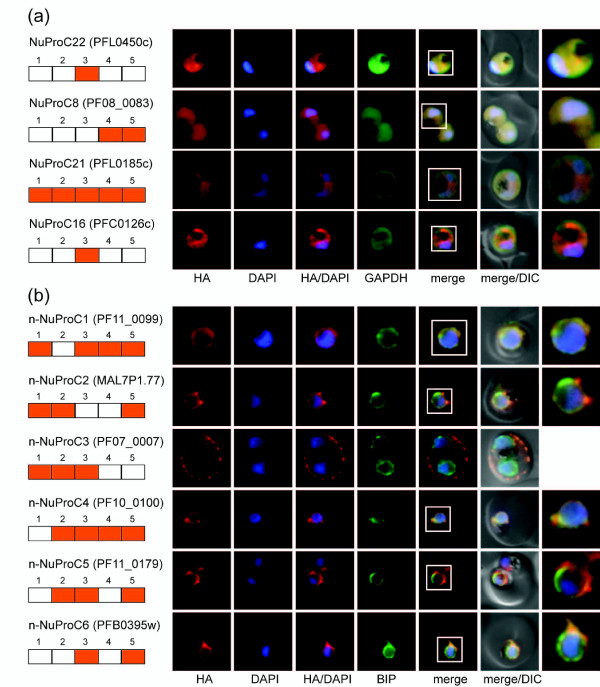
**Experimental validation of the core nuclear proteome by IFA - non-nuclear localization**. All panels show protein localization in trophozoites. Protein acronym and PlasmoDB gene accession numbers are indicated to the left. Peptide detection or absence in individual fractions is indicated by orange or white boxes, respectively. Epitope-tagged candidates were detected using anti-HA antibodies (red). The reference compartments cytosol and ER were visualized using antibodies against GAPDH and PfBIP, respectively (green). White frames refer to the magnified view of the nuclear area shown in the rightmost images. **(a) **Four NuProCs do not co-localize exclusively with the DAPI-stained area of the parasite nucleus. **(b) **None of the six non-nuclear protein candidates (n-NuProCs) localize to the parasite nucleus, and n-NuProCs 1, 2, 4, 5, and 6 co-localize with the ER marker PfBIP.

The remaining four of the 22 tagged NuProCs showed a fluorescence pattern inconsistent with nuclear localization (Figure 6a). NuProC21 (nucleosome assembly protein PfNAPL; PFL0185c), NuProC8, and NuProC22 co-localized to various degrees with GAPDH throughout the IDC suggesting a cytosolic/cytoplasmic localization. Interestingly, NuProC22 occurred in close proximity to the nuclear periphery in late and segmented schizonts indicating that this protein may be transiently associated with the parasite nucleus. NuProC16, currently annotated as putative PFMNL-1 CISD1-like iron-sulphur protein, localized to a cytoplasmic structure distinct from the cytosolic and ER compartments, which likely represents the mitochondrion. This conclusion is supported by the presence of an iron-binding zinc finger domain CDGSH [69] found in the outer mitochondrial membrane protein MitoNEET in vertebrates [70].

None of the six non-nuclear protein candidates localized to the nucleus. Five co-localized with PfBIP throughout the IDC, and n-NuProC3 was associated with the ER in early stages and exported into the host cell in trophozoites and schizonts (Figure 6b) (detailed IFA localization results throughout the IDC for all n-NuProC cell lines are available in Additional file 13). The strong representation of ER-localized proteins in the negative control set indicates that the crude nuclear preparation was enriched in ER-associated proteins. However, we cannot exclude the possibility that ER association of some of these proteins may be artefactual due to over-expression of the tagged proteins.

In summary, our experimental validation of the predicted core nuclear proteome tested 16 out of 22 nuclear protein candidates (72.7%) positive for true nuclear localization, plus another two that are associated with the nuclear periphery. This figure is consistent with the estimated precision of the bioinformatically reduced core set of nuclear proteins and validates this inventory as an important platform for the identification and characterization of novel parasite nuclear proteins.

### Two novel nucleolar proteins in *P. falciparum*

**Figure 7:**
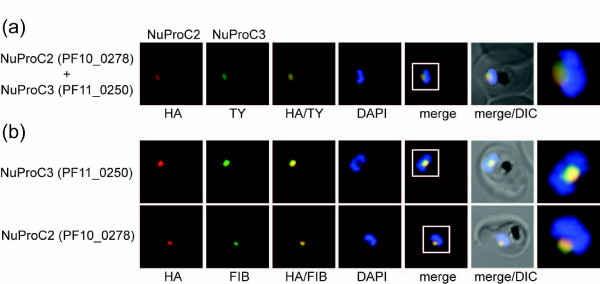
**Identification of two novel *P. falciparum *nucleolar proteins**. **(a) **IFA co-localization of NuProC2-3xHA and NuProC3-2xTy in a double transgenic line. Epitope-tagged proteins were detected using anti-HA (red) and anti-Ty (green) antibodies. **(b) **NuProC2-3xHA and NuProC3-3xHA co-localize with nucleolar fibrillarin. HA-tagged proteins were detected in single transgenic lines using anti-HA antibodies (red). Fibrillarin (PfFIB) was visualized using an anti-human fibrillarin antibody [71,72].

To test if the two distinct subnuclear domains delineated by NuProC2 and NuProC3 represented the same compartment we generated a parasite line expressing both proteins simultaneously. IFA analysis revealed that both proteins co-localized within the same intra-nuclear compartment that was preferentially located towards the nuclear periphery in a DAPI-negative area (Figure 7a). A monoclonal antibody directed against the human nucleolar marker fibrillarin [71] that is cross-reactive with *Toxoplasma gondii *fibrillarin [72], specifically recognized the same compartment as NuProC2 or NuProC3 (Figure 7b). Hence, these co-localization experiments identified two novel nucleolar proteins in *P. falciparum*.

### Identification of novel domains associated with *P. falciparum *nuclear proteins

Using the core nuclear proteome as an input collection, we sought to detect novel functional domains enriched in nuclear proteins using an all-*versus*-all BLAST search followed by manual inspection (Additional file 1). Six novel domains comprised of 17 proteins warranted further inspection, all of which were conserved across Apicomplexa providing further evidence that these modules are functional.

**Figure 8:**
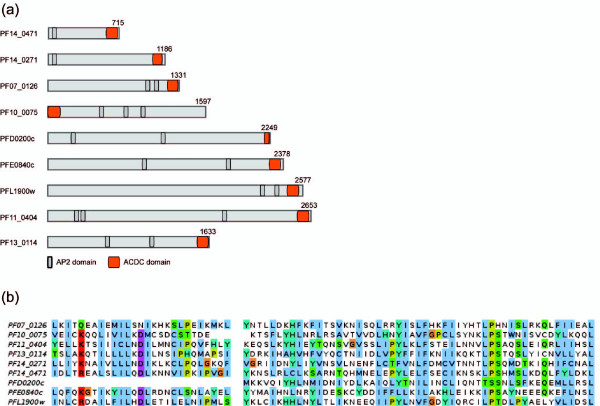
**Schematic of the novel ACDC domain in *P. falciparum *proteins**. (a) The novel ACDC domain was found in nine *P. falciparum *ApiAP2 proteins. The figure was generated with DomainDraw [134] and modified with Inkscape and Adobe Illustrator. (b) Multiple sequence alignment of the nine *P. falciparum *ACDC domains is shown. The vertical gap signifies a short, less conserved region omitted for clarity.

One of these domains (approximately 80 aa) was found exclusively in ApiAP2 proteins [40,73,74]. It occurred in nine *P. falciparum*, 15 *T. gondii*, and two *Babesia bovis *proteins, and in one protein from each of the sequenced *Cryptosporidium *spp. (Additional files 14, 15 and 16). Unlike AP2 domains, this novel domain was only ever found once in each protein. Interestingly, this domain was located at the C-terminus in all but one of the *P. falciparum *ApiAP2s where it is found at the N-terminus (Figure 8). This relative position appears to be at least moderately conserved evolutionarily with 42 of 56 proteins having this domain located <50 amino acids away from the C-terminus. Hence, we termed this novel domain ACDC (AP2-coincident domain mostly at the C-terminus). The noticeable co-occurrence of these domains in a family of apicomplexan DNA-binding proteins invites testable hypotheses about potential roles of the ACDC domain in gene expression, chromatin structure, and/or other aspects of chromosome biology.

**Figure 9:**
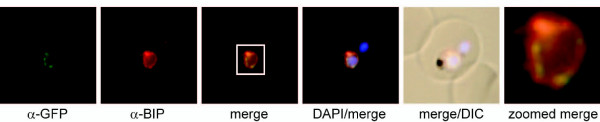
**Identification of a novel nuclear pore component in *P. falciparum***. PF14_0442 represents a novel *P. falciparum *nuclear pore candidate. Endogenous GFP-tagged PF14_0442 and PfBIP were visualized in 3D7/pH_0442-GFPint using anti-GFP (green) and anti-PfBIP (red) antibodies, respectively. The white frame refers to the magnified view shown in the rightmost image.

Four of the five other novel domains are associated with either cleavage of mRNA 3' UTRs (partial cleavage stimulation factor (CSTF) domain), transcriptional regulation (extended ELM2 domain, MYND domain), or the cytoskeleton (alveolin domain) (Additional files 1, 14, 17, 18, and 19). A further domain identified in our search was encoded by two proteins annotated as PfNUP100 (PFI0250c) and a hypothetical protein (PF14_0442). The localization of NUP100 to the nuclear membrane [28], and the fact that much of the conservation between the two proteins lies in the phenylalanine-glycine (FG) pairs of amino acids, suggested that both are FG-repeat nuclear pore components [75] (Additional file 20). To verify if PF14_0442 encodes a nuclear pore protein we generated a transgenic cell line expressing endogenously tagged PF14_0442-GFP (Additional file 21). Indeed, IFA analysis revealed that this protein localized to the nuclear rim, internal to the ER, with several foci of higher intensity, a staining pattern reminiscent of nuclear pores (Figure 9).

### Classical nuclear localization signals are only marginally over-represented in *P. falciparum *nuclear proteins

Classical nuclear localization signals (cNLSs) have been the subject of several bioinformatic prediction algorithms, and our core nuclear proteome provided an opportunity to test their utility in identifying *P. falciparum *nuclear proteins. We used three different bioinformatic tools to predict cNLSs: NLStradamus [76], predictNLS [77], and cNLS mapper [78]. All three algorithms attempt to predict nuclear localization based solely on the presence of cNLSs and not other protein domains.

NLStradamus, PredictNLS, and cNLS mapper suggest 51%, 37%, and 22% of proteins found in the core nuclear proteome contain a cNLS, respectively (Additional file 10), compared to 45%, 31%, and 17% of the entire *P. falciparum *proteome (*P *values 0.008, 0.006, and 0.006, respectively; Fisher's exact test). In contrast, only 47%, 32%, and 18% of proteins found in the whole cell trophozoite proteome [17] were predicted by these algorithms (*P *values 0.17, 0.4, and 0.18, respectively, relative to all *P. falciparum *proteins; Fisher's exact test). While this result adds some evidence to the hypothesis that cNLS-mediated nuclear import may operate in *P. falciparum *similarly to model eukaryotes, the difference in the percentage of cNLS-containing proteins in the core nuclear proteome compared to the set of all *P. falciparum *proteins is only marginal. Likewise, the same three predictors identify only 50% (NLStradamus), 37% (PredictNLS), and 28% (cNLS mapper) of proteins in the combined ApiLoc and literature review reference set of 317 *P. falciparum *nuclear proteins to contain a cNLS (*P *values 0.08, 0.07, and 0.0004, respectively, relative to all *P. falciparum *proteins; Fisher's exact test). Using more stringent bioinformatic filters on the core nuclear proteome resulted only in a slightly higher percentage of proteins with predicted cNLS (Additional file 22). Together, the low percentage of nuclear proteins being predicted suggests that either *P. falciparum *cNLSs may be too divergent for current bioinformatic predictors, or that the major mode of nuclear import may not occur through classical import signals, or a combination of both.

## Discussion

To deepen our insight into nuclear biology in *P. falciparum *we performed a comprehensive proteomic analysis of the parasite nucleus and detected a total of 1,273 proteins in sequential extracts of crude nuclei. The proportion of predicted nuclear proteins in this set was estimated at 46%, which compares well with two similar studies in *S. cerevisiae*. Mosley et al. detected 2,674 yeast proteins in crude nuclei, 46% of which are annotated as nuclear proteins in the *Saccharomyces *genome database SGD [79], and a second study based on sucrose gradient purification of nuclei detected 1,889 proteins, 55% of which were annotated as nuclear in SGD [80].

The major sources of contamination in nuclear preparations are membrane fractions of non-nuclear organelles, particularly the ER. To eliminate such likely contaminants we applied an informed bioinformatic filtering approach that resulted in a markedly improved positive predictive value (76%) for true nuclear proteins in the core nuclear proteome. Importantly, we confirmed the specificity of this set by *in-vivo *experimental validation. Out of 22 candidates 18 localized in different patterns to the nucleus. Some proteins displayed a rather ubiquitous distribution within the nucleus, whereas others localized to more restricted, undefined subnuclear regions, to the nucleolus, or to the nuclear periphery. These alternative localizations are a likely consequence of the different functions these factors carry out in the nucleus. Four candidates localized primarily to the cytoplasm, however, some of them may also be nuclear. First, we determined localizations using episomally expressed epitope-tagged proteins, which may cause over-expression and cytosolic accumulation of some candidates. Second, proteins primarily located in the cytosol may still be present in the nucleus in lower concentrations. For instance, PfNAPL (NuProC21), which localized to the cytoplasm here and in a previous study [81], is a putative orthologue of ScNAP1 that shuttles between the nucleus and cytosol [82]. Another example is PFL0450c (NuProc22), a protein with unknown function that localized to the cytoplasm in ring and trophozoite stages but was found at the nuclear periphery in late schizonts.

### Functional classification of the core nuclear proteome

We detected 299 proteins (37.3%) implicated in nuclear processes such as transcription, chromatin remodeling, DNA replication/repair, RNA binding and processing, ribosome biogenesis, but also in more general processes such as protein folding and modification, protein degradation, translation, cytoskeleton organization, and metabolism. This classification is based on direct experimental evidence, literature review, and/or sequence similarity to known *S. cerevisiae *nuclear proteins (Additional file 10). A total of 118 proteins (14.7%), for which such evidence is missing, carry annotations similarly consistent with roles in DNA/chromatin interactions, cell cycle control, mitosis, RNA binding and processing, protein folding and modification, protein degradation, cytoskeleton organization, and metabolic processes. Ninety-five proteins (11.8%) represent ribosomal subunits or translation-associated factors, and 35 proteins (4.4%) are predicted to localize to other compartments such as the mitochondrion, ER/Golgi, protein sorting vesicles, or the vacuole. Finally, the largest fraction of the core nuclear proteome consists of 255 proteins of unknown function (31.8%).

To date, only three proteins have been localized to the parasite nucleolus (RNA polI, PFE0465c; fibrillarin/PfNOP1, Pf14_0068; PfNOP5, PF10_0085) [83,84]. Further, only six *P. falciparum *proteins are annotated with the GO term 'nucleolus', and 36 are annotated by GeneDB [85] as putative nucleolar proteins. Here, we detected 12 of these proteins including fibrillarin, PfNOP5, RNA methyltransferase (PF11_0305), putative ribonucleoprotein (RNP) components such as LSM homologs (PFL0460w, PF08_0049), and several predicted pre-ribosomal assembly proteins, as well as another 19 putative homologs of *S. cerevisiae *nucleolar proteins (Additional file 10). Notably, two of these candidates, NuProC2 (PF10_0278) and NuProC3 (PF11_0250), co-localized with fibrillarin to the parasite nucleolus. These novel identifications expand our current knowledge of the *P. falciparum *nucleolus and provide a basis for detailed analyses of this essential nuclear compartment.

*Plasmodium *protein kinases (PK) play central roles in growth, development, and differentiation throughout the life cycle, and are intensely studied as a class of promising antimalarial targets [86,87]. We identified kinases or their accessory factors of seven PK systems, six of which have been implicated in nuclear roles in *Plasmodium *or other eukaryotes: PfMAT1 (PFE0610c) [88]; the α- and β-subunits of casein kinase 2 (PF11_0096, PF11_0048) [89-91]; casein kinase 1 (PF11_0377) [92,93]; the catalytic and regulatory subunits of cAMP-dependent protein kinase (PFI1685w, PFL1110c) [94]; NIMA-related protein kinase PfNEK-1 (PFL1370w) [95,96]; and mitogen-activated protein kinase 2 PfMAP2 (PF11_0147) [97,98]. PfMAP2 is a substrate of PfNEK-1 [96] and both kinases are essential for completion of the IDC in *P. falciparum *[95,99], whereas in *P. berghei *PbMAP2 is implicated specifically in the development of male gametes [97]. Interestingly, while the targets of these and other nuclear kinases remain largely unknown, we find that 70% of proteins in the core nuclear proteome (562/802) were recently shown to be phosphorylated [100] (Additional file 10) suggesting an important role for kinase signalling in the regulation of nuclear processes in *P. falciparum*.

We detected 32 of the 33 annotated *P. falciparum *proteasome components and all, except two of the 19S regulatory particle (RP) subunits, were represented in the cytoplasmic fraction. Intriguingly, all subunits of the RP were also associated with the insoluble nuclear fraction in ring stage parasites (Additional file 10). In other species, the RP interacts with chromatin and is involved in transcriptional regulation [101]. For example, the *S. cerevisiae *RP participates in SAGA histone acetyltransferase complex recruitment [102], interacts with the FACT (facilitates chromatin transcription) complex [103], and influences H3 methylation and gene silencing [104]. Hence, we speculate that the 19S RP may have similar non-proteolytic roles in *P. falciparum *transcriptional regulation.

The nuclear proteome also contained several orthologues of the mRNP complex implicated in translational repression during *P. berghei *gametocytogenesis [105,106], such as the RNA helicase PfDOZI (PFC0915w), PfCITH (PF14_0717), the RNA-binding proteins PfHOBO (PF14_0096) and PfHOMU (PFI0820c), and a homolog of a yeast poly(A)-binding protein (PFL1170w). Although DOZI and CITH were described as cytoplasmic proteins in *P. berghei*, their localizations do not appear to exclude the nucleus. Furthermore, homologs of PfHOMU and PFL1170w shuttle between the cytosol and nucleus in mammalian cells and *S. cerevisiae*, respectively [107,108]. The detection of these proteins in the asexual nuclear proteome suggests the presence of translational repression machinery during the IDC, although *P. berghei *DOZI and CITH loss-of-function mutants have no apparent phenotype in asexual parasites [105,106].

We also discovered novel domains that are likely linked to previously unrecognized nuclear functions of parasite proteins, and we envisage their roles will be more thoroughly recognized in future studies. Of particular interest is the identification of the ACDC domain, which we identified exclusively in members of the ApiAP2 family. Moreover, we experimentally validated the identification of PF14_0442 as a novel subunit of *P. falciparum *nuclear pores. Testing this in more detail will be an important future task given that *Plasmodium *parasites lack identifiable orthologues of most nuclear pore components [109].

### Stage- and fraction-specific aspects of the core nuclear proteome

We made several interesting observations regarding stage- and fraction-specific protein profiles that allow us to speculate about potential temporal and spatial expression patterns of nuclear proteins. In light of the stochastic undersampling of complex proteomes in shotgun proteomics approaches and possible sample-to-sample variation, however, these data have to be interpreted with caution.

A total of 58, 90, and 105 proteins were found exclusively in ring, trophozoite, and schizont stages, respectively. In trophozoites and schizonts, expression of these proteins occurred roughly in line with transcription of the encoding genes (Additional files 1 and 23). mRNA expression of ring stage-specific nuclear proteins was somewhat surprising, with a collective profile similar to that observed in schizonts, and an additional smaller peak at 15 to 20 hpi. This suggests that some proteins were newly synthesized in the ring stage while some remained from the preceding schizont stage. A total of 159 of the 253 stage-specifically detected proteins carry no functional annotation and several others carry annotated domains that indicate little about their function. Hence, a large number of novel proteins have been assigned a potential stage-specific role in nuclear biology where previously no informative annotation was available. Further, 13 predicted TFs [63] were detected specifically in a single IDC stage only, indicating a role for these factors as cell cycle-specific regulators of transcription and genome regulation (Additional file 24).

Of 145 proteins predicted to interact with DNA and/or chromatin, 137 (94.5%) were identified in the chromatin-containing fractions (DNAseI-, high salt-, and/or SDS-soluble fractions) (Additional file 10). Some of these factors, such as histones, SNF2 helicase (PFF1185w), chromodomain-helicase-DNA-binding protein 1 (CHD1) (PF10_0232), putative chromosome assembly factor 1 (PFE0090w), and the nuclear peroxiredoxin PfnPRX (PF10_0268) [110] were detected in all three chromatin-associated fractions. In contrast, nine out of the 10 RNA pol II subunits identified were exclusively detected in the high salt nuclear extract. Both FACT components (PFE0870w, PF14_0393) extracted almost identically only after high salt and SDS extraction. The two recently described high mobility group box proteins (PFL0145c, MAL8P1.72) [111] were identified in DNAse1- and high salt-soluble extracts but not in the SDS fraction. These examples suggest that at least some regulatory complexes were extracted as interacting entities. Interestingly, all ApiAP2 factors showed a noticeable association with the insoluble nuclear fraction. While the reason for this remains unknown our observation hints at possible functions of these DNA-binding proteins. Most ApiAP2 proteins are large and, apart from the short and well-defined DNA-binding AP2 domains, consist of extensive uncharacterized regions. It is possible that these non-AP2 regions may mediate the formation of regulatory complexes involved in diverse processes such as DNA replication, transcriptional regulation, or functional organization of the genome. Such complexes are often resistant to extraction with DNAseI and high salt buffers, as observed for many RNA- or DNA-binding proteins associated with the nuclear matrix [112]. In case of PfSIP2, the only *P. falciparum *ApiAP2 characterized *in vivo*, the detection of PfSIP2-derived peptides in the insoluble nuclear fraction is consistent with the association of this factor with condensed heterochromatin [46].

A large number of proteins in the core nuclear proteome (379) were also detected in the cytoplasmic fraction. This pool of proteins contained members of all functional classes but was clearly enriched in distinct pathways associated with the various functions of the nucleolus in other eukaryotes [113-115]. These include the majority of ribosomal subunits (90.5%), RNA-binding proteins (78.9%), factors involved in protein degradation (69.0%), and protein folding and modification (65.1%). Furthermore, 84.4% of translation-related factors were identified in the cytoplasmic and nuclear fractions, a finding consistent with the existence of nuclear translation [116,117]. We also noticed that 42% (62 proteins) of confirmed or likely nuclear proteins in the core nuclear proteome were detected in the cytoplasmic fraction. Moreover, six proteins experimentally localized to the nucleus in this study had peptides detected in the cytoplasmic fraction. Hence, while some of the dually detected proteins may represent cytoplasmic contaminants such as abundant cytosolic proteins or macromolecular complexes, our results show that many nuclear proteins in *P. falciparum *shuttle between the nucleus and cytosol and/or perform their tasks at multiple destinations.

### Nuclear import in *P. falciparum*

Transport of proteins into the nucleus remains poorly understood in apicomplexan parasites [118]. In yeast and mammalian model systems short, arginine-, and lysine-rich cNLSs are thought to be the major mediators of nuclear import, though alternative and redundant mechanisms have been described [119]. The poor enrichments of cNLSs in both the core nuclear proteome and the set of 317 curated nuclear proteins show that bioinformatic discrimination of nuclear vs. non-nuclear *P. falciparum *proteins via prediction of cNLSs remains impractical. Notably, however, cNLS predictors are also problematic in the reliable identification of nuclear proteins in the model systems they were designed for [120]. Nevertheless, differences between *P. falciparum *nuclear proteins and these current computational models of cNLSs appear prevalent but remain unclear. It has not been determined whether the majority of *P. falciparum *nuclear proteins are imported independently of importin α, the protein that binds cNLSs [118], or alternately by similar but unrecognized cNLSs that do mediate importin α-dependent translocation. The fact that only 22% to 51% of nuclear proteins are predicted to contain cNLSs (depending on the predictor used) suggests that in its current definitional form the cNLS is not the major mode of nuclear import in *P. falciparum*. Notably, the important insight that most verifiable *Plasmodium *nuclear proteins lack a recognizable nuclear localization sequence, and need thus be identified through empirical strategies, reinforces the value of an experimentally robust nuclear proteome for understanding *Plasmodium *nuclear biology.

### Lineage-specific nuclear proteins

Using a combination of OrthoMCL and synteny analyses (Additional files 1 and 25) only around 10 proteins are genuinely *falciparum*-specific. Unsurprisingly, nearly all of them have no functional annotation, though one is the gametocyte-specific protein Pfg27 (PF13_0011). Pfg27 binds RNA and has previously been localized to the cytoplasm and nucleus of gametocytes [121]. Interestingly, we detected another nuclear *P. falciparum*-specific protein of unknown function (PFB0115w) that is expressed during the IDC and in gametocytes and contains C-terminal homology to the Pfg27-specific fold [122] suggesting a functional relation between these two proteins.

The pool of around 100 genus-specific proteins represents a promising group from which to characterize features of nuclear biology peculiar to *Plasmodium*. Several are potentially worthy of prioritized treatment; these include some of the previously characterized ApiAP2s [40], as well as a large number of uncharacterized proteins with nucleic acid-binding and chromatin-interacting domains. Several others in this group have kinase or phosphatase domains and may be involved in expression regulation cascades. One of the *Plasmodium*-specific nuclear proteins is a putative metacaspase (PF14_0363). Interestingly, unrelated trypanosomatid parasites also possess nuclear localized metacaspases that are required for proliferation, possibly through modulation of cell cycle progression [123].

## Conclusion

To our knowledge, this is the most detailed organellar proteome analysis in apicomplexan parasites and the first study describing a large scale nuclear proteome of any protist species. During the past decade several intriguing features of nuclear biology in *P. falciparum *have been discovered, including the periodical behavior of gene transcription during the IDC [21,22], the discovery of the ApiAP2 family [40], the epigenetic control of genes related to virulence [29,30,33-35,124], or the fascinating dynamic surface distribution of nuclear pores throughout the IDC [12]. These examples highlight the existence of nuclear processes distinct from those in the host and underscore the importance of understanding the underlying mechanisms. For the first time, our study provides a comprehensive and unprecedented insight into the biology of the *P. falciparum *nucleus, and the nuclear proteome presented herein will assist greatly in dissecting conserved as well as evolutionary specialized nuclear processes in *Plasmodium *and related parasites.

## Materials and methods

### Parasite culture and transfection

*P. falciparum *3D7 parasites were cultured as described previously [125]. Growth synchronization was achieved by repeated sorbitol lysis [126]. Transfections were performed as described [127] and selected on 5 μg/mL blasticidin-S-HCl and/or 4 nM WR99210. Parasites transfected with pH_0442-GFP were cycled on/off WR three times to select for integration of the plasmid into the endogenous locus.

### Isolation of nuclei and nuclear fractionation

Nuclei from ring stages, trophozoites, and schizonts were isolated and fractionated as previously described with minor modifications [35]. A detailed protocol is provided in Additional file 1. For western blot analysis protein fractions were separated on 10% SDS-PAGE gels and transferred to nitrocellulose membranes (Schleicher&Schuell). Primary antibody dilutions were: anti-H4 (Abcam, ab10158) 1:10,000; anti-HSP60 (kind gift from Geoff McFadden, University of Melbourne) 1:2,000; mAb anti-PfGAPDH 1:2,000 [128]; rabbit anti-PfHP1 (kind gift from Mike Duffy, University of Melbourne) 1:2,000; and anti-GFP (Roche Diagnostics, 11814460001) 1:1000.

### Microscopic assessment of isolated nuclei

Nuclei were incubated with DAPI (10 μg/mL) in CLB for 10 min on ice and directly observed by light and fluorescence microscopy. Images were obtained using a Leica DM 5000B microscope with a Leica DFC 300 FX camera and acquired via the Leica IM 1000 software and processed and overlayed using Adobe Photoshop CS2. For TEM analysis isolated nuclei or whole asynchronous 3D7 cultures were fixed, prepared for microscopy and imaged as described [129]. For immunolabelling, nuclei were incubated with rabbit anti-H3K4Me3 (Abcam ab8580) diluted 1:300, then detected with 18 nm colloidal gold-conjugated goat anti-rabbit secondary antibody (Jackson ImmunoResearch, Baltimore, MD, USA) diluted 1:20.

### Sample preparation, MudPIT liquid chromatography tandem mass spectrometry, and protein identification

Detailed protocols for sample preparation, MudPIT analysis, and protein identification are provided in Additional file 1. All proteomics data are publicly available on PlasmoDB [68] and in Additional files 3, 4, and 5.

### Bioinformatic data analysis

Bioinformatic methods used to assess and analyze the nuclear proteome are presented in detail in Additional file 1.

### Transfection constructs

To express epitope-tagged candidate proteins in *P. falciparum*, full-length genes were amplified from gDNA or cDNA and cloned into pBcam-3xHA [35] using *BamH*I (or *Bgl*II) and *Nco*I (or *Nhe*I) restriction sites. The PFL0635c gene (NuProC6) was cloned into *BamH*I/*Nhe*I-digested pHcam-2xTy [46]. To generate the double transfectant co-expressing NuProC2-3xHA/NuProC3-2xTy the PF11_0250 gene (NuProC3) was cloned into *BamH*I/*Nco*I-digested pHcam-2xTy and transfected into BSD-resistant 3D7/NuProC2-3xHA parasites. All forward primers included five wild-type nucleotides directly upstream of the natural ATG start codon. pH_0442-GFP was obtained by cloning the 3' end of PF14_0442 (551 bp) into *Pst*I/*Not*I-digested pHcam-GFP [130]. Successful 3' replacement integration of pH_0442-GFP into the endogenous locus was verified by PCR and western blot (Additional file 21). Primer details are listed in Additional file 26.

### Indirect immunofluorescence assays

IFAs were performed with iRBCs fixed in 4% formaldehyde/0.01% glutaraldehyde as described elsewhere [131]. Primary antibody dilutions were: rat mAb anti-HA 3F10 (Roche Diagnostics) 1:100; mouse mAb anti-Ty BB2 1:2,000 (kind gift from Keith Gull); mouse mAb anti-GAPDH 1:500 [128]; rabbit anti-PfBIP 1:500 [132,133] (kind gift from Tim Gilberger); and mouse mAb anti-fibrillarin 1:500 [71] (kind gift from Michael Terns, University of Georgia). Secondary antibody dilutions were: Alexa-Fluor^® ^568-conjugated anti-rat IgG (Molecular Probes) 1:500; Alexa-Fluor^® ^488-conjugated anti-rabbit IgG (Molecular Probes) 1:500; and FITC-conjugated anti-mouse IgG (Kirkegaard Perry Laboratories) 1:250. Images were taken on a Leica DM 5000B microscope with a Leica DFC 300 FX camera and acquired via the Leica IM 1000 software and processed and overlayed using Adobe Photoshop CS2. Observed protein localizations have been deposited in ApiLoc [64].

## Competing interests

The authors declare that they have no competing interests.

## Authors' contributions

SO performed experiments related to nuclear isolation and fractionation, generation of transgenic cell lines, and IFA analysis, and participated in the design and coordination of the study and in writing the manuscript. BJW performed bioinformatic data analysis, and participated in the design of the study and in writing the manuscript. SM prepared and analyzed samples by MudPIT. JW, OD, and AP generated transgenic cell lines and performed IFA analyses. CD performed the electron microscopy experiments. PM participated in data analysis. CF, KW, NMBB, and IN performed experiments and participated in the coordination of this study. PJ and SAR participated in the design, coordination, and interpretation of the study, and critically revised the manuscript. TSV performed experiments related to nuclear isolation and fractionation, conceived of the study, participated in the design, coordination and interpretation of the study and in data analysis, and wrote the manuscript. All authors read and approved the final manuscript.

## Supplementary Material

Additional file 1 **Detailed protocols for all experimental and bioinformatic analyses performed in this study (1a to 1f)**. Click here for file

Additional file 2 **Summary table of tryptic peptides measured by MudPIT and Spearman rank correlations between replicates**. Click here for file

Additional file 3 **Raw SEQUEST output data for peptide tandem mass spectra and proteins detected in ring stage parasites**. Click here for file

Additional file 4 **Raw SEQUEST output data for peptide tandem mass spectra and proteins detected in trophozoites**. Click here for file

Additional file 5 **Raw SEQUEST output data for peptide tandem mass spectra and proteins detected in schizonts**. Click here for file

Additional file 6 **Extensive summary table for all proteins detected in this study**. Click here for file

Additional file 7 **Enrichment analyses for KEGG pathways and GO terms in proteins found only in the combined nuclear or cytoplasmic fractions**. Click here for file

Additional file 8 ***P. falciparum *protein localizations derived from published sources through homology**. Click here for file

Additional file 9 **Proteins removed from the bioinformatically filtered nuclear proteome (worksheet A)**. Proteins re-added to the nuclear proteome after they were removed by bioinformatic filtering (worksheet B). Proteins with recently added signal peptide annotation (worksheet C). Proteins with recently removed signal peptide annotation (worksheet D). Click here for file

Additional file 10 **Extensive summary table for all proteins in the core nuclear proteome**. Click here for file

Additional file 11 **List of proteins tagged and localized in this study**. Click here for file

Additional file 12 **Detailed IFA localization of NuProCs 1 to 22 during the IDC**. Click here for file

Additional file 13 **Detailed IFA localization of n-NuProCs 1 to 6 during the IDC**. Click here for file

Additional file 14 **Multiple sequence alignments in graphical format of: (1) the ACDC domains in apicomplexan proteins; (2) the apicomplexan proteins encoding the partial CSTF domain; (3) the *P. falciparum *proteins encoding the extended ELM2 domain; (4) the *P. falciparum *proteins encoding the MYND domain**. Click here for file

Additional file 15 **A multiple sequence alignment of the apicomplexan proteins encoding the ACDC domain is shown in aligned FASTA format**. This file can be viewed using JalView [135]. Click here for file

Additional file 16 **HMM used to query the *P. falciparum *and other alveolate whole proteomes to detect ACDC domains, in stockholm format**. Note that this is not an HMM built from all putative ACDC domains, but simply an intermediate file used during the methodology of this publication. This file can be viewed using LogoMat-P [136]. Click here for file

Additional file 17 **A multiple sequence alignment of the apicomplexan proteins encoding the partial CSTF domain in aligned FASTA format**. This file can be viewed using JalView [135]. Click here for file

Additional file 18 **A multiple sequence alignment of the apicomplexan proteins encoding the extended ELM2 domain in aligned FASTA format**. This file can be viewed using JalView [135]. Click here for file

Additional file 19 **A multiple sequence alignment of the apicomplexan proteins encoding the MYND domain in aligned FASTA format**. This file can be viewed using JalView [135]. Click here for file

Additional file 20 **Pairwise sequence alignment of the two putative FG-repeat proteins PFI0250c and PF14_0442**. Click here for file

Additional file 21 **PCR and western blot analysis to confirm C-terminal tagging of endogenous PF14_0442 by 3' replacement**. Click here for file

Additional file 22 **cNLSs prediction in *P. falciparum *nuclear proteins**. Click here for file

Additional file 23 **mRNA expression profiles and maximal hour of mRNA expression of proteins found stage-specifically in the nuclear proteome**. Click here for file

Additional file 24 **List of stage-specifically detected TFs**. Click here for file

Additional file 25 **Summary table for lineage-specific proteins in the core nuclear proteome**. Click here for file

Additional file 26 **List of all primers used in this study**. Click here for file
