# Supplementary material for: Organellar proteomics reveals hundreds of novel nuclear proteins in the malaria parasite Plasmodium falciparum
Source: Genome Biol. 2012 Nov 26;13(11):R108. doi: 10.1186/gb-2012-13-11-r108 (PMC4053738; doi:10.1186/gb-2012-13-11-r108)
Supplement: Additional file 1 — Detailed protocols for all experimental and bioinformatic analyses performed in this study (1a to 1f). [file gb-2012-13-11-r108-S1.PDF]

Additional file 1_Oehring et al.

Detailed protocols for all experimental and bioinformatic analyses performed in this study:

1a. Schematic of the experimental approach for the purification and fractionation of parasite nuclei and 1D-SDS PAGE analysis of all protein fractions visualised by silver staining (page 2).

1b. Detailed protocols used for nuclear isolation and fractionation, sample preparation for MudPIT analysis, tandem mass spectrometry and peptide identification (page 3).

1c. Detailed protocols used for bioinformatic analyses and validation of the core nuclear proteome (page 5).

1d. Rationales for acceptance or rejection of bioinformatics filters (page 9).

1e. Protocol for the identification of novel protein domains (page 11).

1f. Protocol for identification of lineage-specific proteins in the core nuclear proteome (page 15).

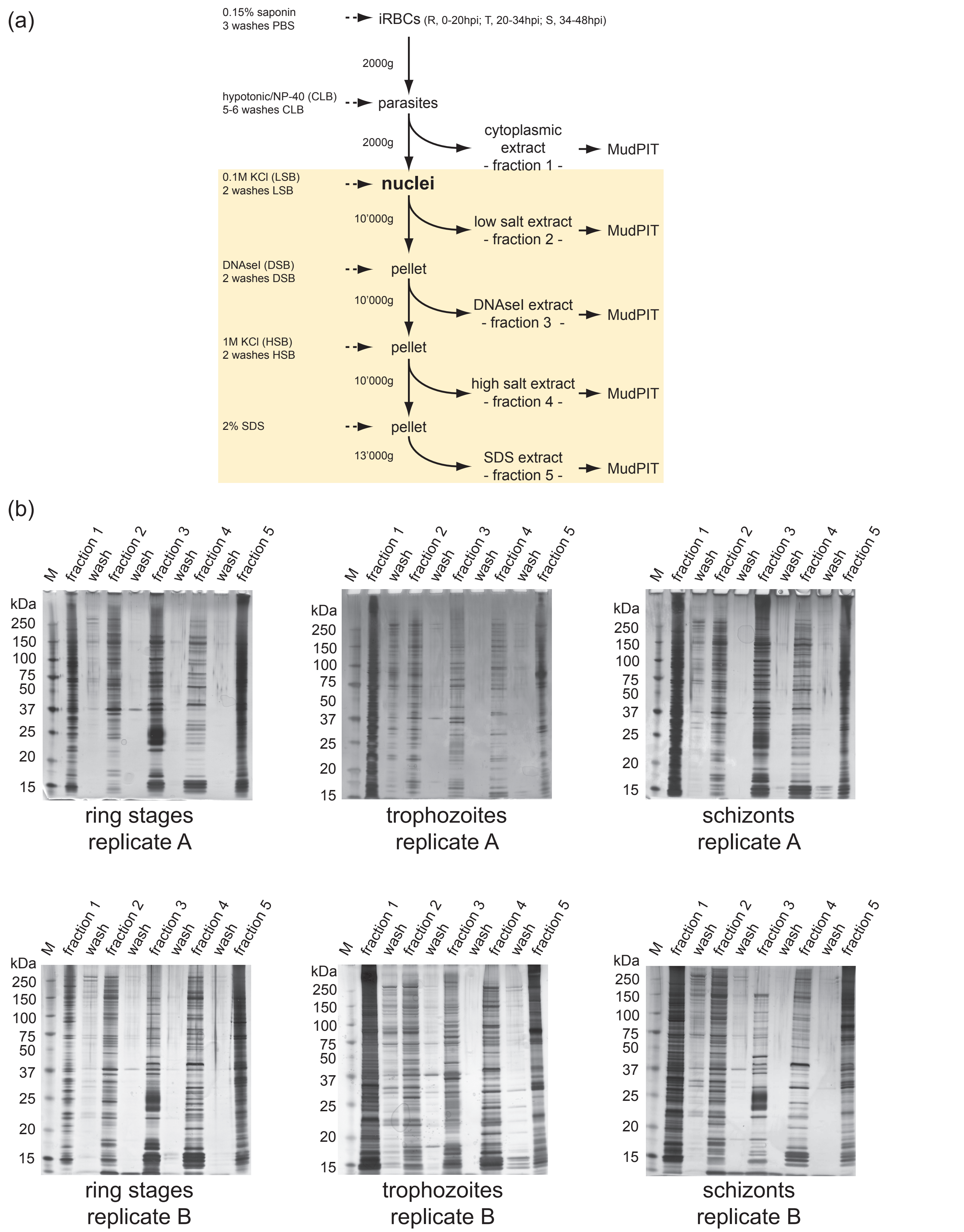

Additional file 1a. Cytoplasmic and nuclear protein fractions. (a) The flowchart illustrates the experimental approach for the purification and fractionation of parasite nuclei. Fraction 1 corresponds to the cytoplasmic extract. The shaded box highlights the differential extraction of nuclear proteins into fractions 2 to 5. Isolation and fractionation has been performed twice each for ring stages, trophozoites and schizonts. (b) 1D-SDS PAGE analysis of all protein fractions obtained from ring stages, trophozoites and schizonts (two replicates each), visualised by silver staining. Fractions 1 to 5 are labelled as in (A). The last wash fraction prior to each subsequent extraction is also shown.

1b. Detailed protocols used for nuclear isolation and fractionation, sample preparation for MudPIT analysis, tandem mass spectrometry and peptide identification.

Isolation of nuclei and nuclear fractionation.

Parasites ($2x10^{10}$ ring stages (2-20 hpi); 10^{10} trophozoites (20-34 hpi); $5x10^9$ schizonts (34-48 hpi) were released from RBCs by saponin lysis and washed three times in PBS. Parasites were lysed in a hypotonic cytoplasmic lysis buffer CLB (20mM HEPES (pH7.9), 10mM KCl, 1mM EDTA, 1mM EGTA, 0.65% NP-40, 1mM DTT, Complete TM protease inhibitors (Roche Diagnostics)) for 5 min on ice. Nuclei were pelleted at 2,000g for 5 min and the supernatant saved (cytoplasmic extract; fraction 1). After four to seven washes in CLB nuclei were extracted in 1.5ml (ring stages and trophozoites) or 3ml (schizonts) low salt buffer LSB (20mM HEPES (pH7.9), 0.1M KCl, 1mM EDTA, 1mM EGTA, 1mM DTT, protease inhibitors) for 20 min at 4°C under constant agitation. Insoluble material was pelleted for 3 min at 13,000rpm and the soluble fraction saved (low salt extract; fraction 2). Pellets were washed twice in 1.5ml (3ml) LSB and chromatin was solubilised in 1.5ml (3ml) DNAseI-digestion buffer DDB (20mM Tris-HCl (pH7.5); 15mM NaCl, 60mM KCl, 1mM $CaCl_2$, 5mM $MgCl_2$, 5mM $MnCl_2$, 300mM sucrose, 0.4% NP-40, 1mM DTT, protease inhibitors) containing 100-500U DNAseI for 20 min under constant agitation at RT and 37°C. Samples were centrifuged at 13,000rpm for 3 min and the soluble fraction was saved (DNAseI extract; fraction 3). After two washes in 1.5ml (3ml) DDB pellets were extracted with high salt buffer HSB (20mM HEPES (pH7.9), 1M KCl, 1mM EDTA, 1mM EGTA, 1mM DTT, protease inhibitors) for 20 min at 4°C under constant agitation. The soluble fraction was recovered by centrifugation at 13,000rpm for 3 min and saved (high salt extract; fraction 4). The insoluble nuclear matrix fraction was washed twice in 1.5ml (3ml) HSB, solubilised in 1.5ml (3ml) SDS extraction buffer SEB (2%SDS, 10mM Tris-HCl (pH 7.5)) for 20 min under constant agitation at room temperature, cleared by centrifugation at 13,000rpm for 15min and saved (SDS extract; fraction 5). The quality of all samples was analysed by SDS-PAGE and silver staining and by Western blot.

Sample preparation for MudPIT LC-MS/MS analysis

Within each set of corresponding fractions from each of the three different parasite stages we attempted to analyse equal amounts of total protein extracted. We therefore estimated the protein concentration in each fraction by inspection of silver-stained SDS-PAGE gels and precipitated adequate volumes in 10% TCA. Protein pellets were washed twice in 1ml ice-cold 100% acetone, air-dried and re-dissolved in 10µl 100mM Tris-HCl, pH 8.0 containing 6M urea and diluted with 20µl 100mM Tris-HCl, pH8.0 to lower the urea concentration to 2M. Digestion was done with 0.25µg endoproteinase LysC (ELC, Roche Diagnostics) for one hour at 37°C followed by a second aliquot of ELC and incubation at 37°C. Subsequently, trypsin digestion was done with 0.25µg trypsin (Promega) at 37°C for 2 hrs followed by a second digestion

with trypsin (0.25μg) and incubation for 18 hrs at 37°C. The digest was stopped by adding 3μl 10% trifluoro acetic acid (TFA). The digest was diluted with 100μl 0.1% TFA/1% acetonitrile and desalted on a MacroSpin column packed with Vydac C18 reverse-phase material (The Nest Group, Southborough, MA) according to the manufacturer's recommendations. The peptides were eluted with 300μl of 80% acetonitrile/0.1% TFA, dried in a speed vacuum system and re-dissolved in 100μl 0.1% formic acid/2% acetonitrile.

MudPIT liquid chromatography tandem mass spectrometry

The desalted peptides were analyzed by two-dimensional capillary liquid chromatography and tandem mass spectrometry using a Polysulfoethyl A ion-exchange column (0.15 x 50 mm, PolyLC, Columbia, MD) connected in series to a C18 trap column (Zorbax 300SB, 0.3 x 50 mm, Agilent Technologies) and a Magic C18 separation column (0.1 x 100 mm, Thermo Scientific). 10 μl of the digest were injected first onto the cation-exchange column. Unadsorbed peptides were trapped on the Zorbax column and eluted onto the separation column with a linear 75 min gradient from 2 to 75% B (0.1% acetic acid in 80% acetonitrile) in solvent A (0.1% acetic acid in 2% acetonitrile). Next, peptides retained by the cation-exchange column were sequentially eluted and trapped onto the C18 trap column with 10μL pulses of 25, 50, 100, 150, 200, 250 and 500mM ammonium acetate, pH 3.3. Peptides eluted by each individual salt pulse were separated by the acetonitrile gradient as described above. The flow was delivered with a Rheos 2200 HPLC system (Thermo Scientific) at 150μL/min. A pre-column splitter reduced the flow to approximately 500nl/min. The eluting peptides were ionized by a Finnigan nanospray ionization source (Thermo Scientific).

Mass spectrometric analysis was carried out on an LTQ Orbitrap hybrid instrument (Thermo Finnigan, San José, CA). The instrument was operated in a data-dependent mode. A survey scan was performed in the Orbitrap between *m/z* 400-1600 Da at 60,000 resolution. The five most abundant ions detected in the survey scan were fragmented and mass analysed in the LTQ part of the instrument. Singly charged ions were omitted from fragmentation. The normalized collision energy was set to 35%. For peptide identification, the MS/MS spectra from each individual salt step-off were searched against a combined *P. falciparum* (www.plasmoDB.org; release 5.5)/human (NCBI, version August 2008) database using the TurboSEQUEST software [1]. False discovery rates (FDR) were estimated by searching against the reversed merged database. The SEQUEST filter parameters were as follows: Xcorr versus charge state was 2.00 for doubly, and 2.50 for triply and 3.00 for quadruply charged ions, respectively; the ΔCN was 0.1, peptide probability was 0.5, and the protein probability was set to 0.01. Single peptide hits were allowed if they matched the above search criteria. The search results from each individual salt step-off were merged with the multiconsensus option of TurboSEQUEST (Additional files 3-5).

1c. Detailed protocols used for bioinformatic analyses and validation of the core nuclear proteome.

Over-Representation of Functional Classes

To examine for enrichments in functional classes associated with nuclear or other processes, we used the tools GOstat [2] and DAVID [3] to check for overrepresented GO terms or KEGG pathways in proteins found only in the combined nuclear fractions or only in the cytoplasmic fraction. Statistically over-represented GO terms (p-value$<10^{-5}$) and KEGG pathways (p-value<0.05) were inspected and manually curated to verify assignments.

Gold Standard Definition for Accuracy Appraisal

Two sources of information about protein localisation were first independently and then together used as a gold standard: ApiLoc version 2 (apiloc.biochem.unimelb.edu.au), and a literature-linked survey of protein localisations (Additional file 8). For each gold standard a protein was classified as (1) nuclear if it was nuclear-localised during any life cycle stage, even if it was dually localised (whether concurrently or not); (2) non-nuclear if it was localised to another location; and (3) unknown if no localisation information was recorded. Proteins annotated only with "cytoplasm" were considered non-nuclear. PF10_0155, PF10_0395, PFE0360c and PFE0285c were excluded due to conflicting information regarding localisation. PF10_0177b was treated as having unknown localisation since it is unclear whether it localises to the outer regions of the nucleus or immediately outside of the [4]. Enrichment (Figures 3A and 4A) and positive predictive value (Figures 3B and 4B) were calculated as per Equations 1 and 2, respectively.

Determination of Enrichment (Equation 1):

$$Enrichment=\frac{n/N}{t/T}$$

n refers to the number of nuclear proteins in the set being appraised. *N* refers to the number of nuclear proteins annotated in the gold standard, *t* refers to the total number of proteins annotated as nuclear or non-nuclear in the gold standard and *T* refers to the total number of proteins in the *P. falciparum* proteome (5446).

Determination of Positive Predictive Value (Equation 2):

$$PPV=\frac{n}{n+c}$$

PPV was the positive predictive value, *n* was the number of nuclear proteins in the set being appraised, and *c* was the number of non-nuclear proteins in the set being appraised. The proteins that were counted as nuclear, non-nuclear and excluded varied according to the gold standard being used.

Determination of Statistical Over-representation of Nuclear Proteins

To determine statistical significance of over-representation of nuclear proteins, Fisher's exact test was applied using R version 2.13.1 (http://r-project.org), with 37 positive, 130 negative, 18 excluded (those above as well as those classified as ER) and 1088 unknown proteins as classified using ApiLoc, and assuming that the positive predictive value was maintained in the proteins of unknown localisation that were detected here by mass spectrometry.
A similar result obtained using the literature survey was found using 136 positive, 55 negative, 7 excluded and 1075 unknown. All further calculations were carried out using the combined ApiLoc and literature review gold standards.

Bioinformatic Contaminant Removal Techniques
Exported proteins were predicted using ExportPred [5], taken from PlasmoDB v6.4 using the default cut-off of 4.3, and PfHP1-association [6]. SPs were firstly predicted using SignalP [7] using default settings at PlasmoDB v6.4. TM domains were predicted using a locally installed version of TMHMM 2.0c [8] and a wrapper script. The wrapper script was subsequently made into a Biogem (http://biogems.info), named bio-tm_hmm. To predict the percentage of *S. cerevisiae* nuclear TM domain proteins, proteins annotated with the GO term [9] "nucleus" (GO:0005634) were downloaded (Oct 11, 2010) from Amigo version 1.7 (www.geneontology.org). Corresponding protein sequences were taken from the SGD [10] on 11 Oct, 2011. Signal peptides were removed prior to TM domain prediction using a locally installed version of SignalP v3.0 and a custom BioRuby [11] wrapper script git version 22593c (github.com/wwood/bbbin/blob/master/signalp.rb) so proteins with a predicted signal peptide but no transmembrane domain predicted were not filtered out when only the TM filter was applied. To predict the number of proteins in the entire *S. cerevisiae* proteome that had TMs, the "orf_trans.fasta" file was downloaded from SGD on Jan 6, 2010 and underwent cleavage of signal peptides and transmembrane domain prediction as above. Proteins found in the food vacuole [12] and Maurer's cleft [13] proteomes were removed by taking the gene IDs from the supplementary data of each publication. Mitochondrial transit peptides were predicted using PlasMit [14], http://gecco.org.chemie.uni-frankfurt.de/plasmit/index.html, accessed June 10, 2010) using PlasmoDB v6.4.
The final core nuclear proteome was defined as the proteins that remained after applying the combined filter (exported; SP positive; TM positive; present in the Maurer's cleft proteome; present in the food vacuole proteome), with four amendments. Firstly, proteins predicted to be non-nuclear according to the ApiLoc or literature review gold standards were removed (Additional file 9). Secondly, proteins predicted as true nuclear proteins by the gold standards but ruled out by the combined bioinformatic filter were re-added (Additional file 9). Thirdly, two proteins recently demonstrated to be associated with the nucleus were added (PF14_0443 [15] and PF10_0268 [16]. Fourthly, proteins recently annotated as having a SP were removed from the core nuclear proteome (Additional file 9), and those where a

previous SP annotation was recently removed were re-added (Additional file 9) (PlasmoDB version 8.0).

Accuracy of the Core Nuclear Proteome

According to the gold standards, the core nuclear proteome contained 148 known nuclear proteins, 652 proteins of unknown location, and two with conflicting localisation information. Under the assumption that 70% of these unknown proteins are nuclear (since this was the estimate after the combined filter was applied), it was estimated that 473 of these unknown proteins were in fact nuclear-localised, giving an overall estimate of positive predictive value of 76% ([70% of 652 + 148] / 802).

Classical Nuclear Localisation Signal Predictors

PredictNLS [17] version 1.0.15 was used with default parameters after installing the debian package from http://rostlab.org/debian/pool/main/predictnls_1.0.15_all.deb. To predict from multiple genes, a custom BioPerl script was created, available from https://github.com/wwood/bbbin/blob/master/predictmnls, and git commit e4730033 was used. NLStradamus [18] version 1.4 was used with default parameters after download of the standalone program from http://www.moseslab.csb.utoronto.ca/NLStradamus. Prediction using cNLS was carried out by the the creation of a custom bioruby [11] plugin (https://github.com/helios/bioruby-gem) cnls_screenscraper (rubygem version 0.1.0, available from http://rubygems.org/gems/bio-cnls_screenscraper), which queried the cNLS [19] webpage (http://nls-mapper.iab.keio.ac.jp/) automatically for each protein in the *P. falciparum* proteome (PlasmoDB v7.0). Positive predictions cutoffs were taken as 8.0 and 7.0 for monopartite and bipartite signals, respectively. Predictions were obtained for 5452 proteins, with the remaining minority of proteins not predicted because they were too short (<19 residues), too long (>5000 residues), or contained 'X' characters in their protein sequence. Fisher's exact tests were applied using R 2.12.0.

Stage-Specific Patterns

3D7 glass slide microarray information was taken from a previous study [20] and linked to the proteomic data by direct matching of PlasmoDB gene IDs. In case of multiple matches, one was taken at random as representative. Plots were generated with ggplot2 [21]. Proteins found stage-specifically were defined as those found in only one of ring, trophozoite or schizont stages. Predicted TFs [22] were intersected with the core nuclear proteome list. The stages where they were found were tabulated and annotated with Affymetrix microarray annotation [23] using PlasmoDB version 6.4.

Similarity between *P. falciparum* and *S. cerevisiae* nuclear proteins

For each protein in the predicted *P. falciparum* proteome (PlasmoDB version 6.3, 5446 proteins), the best match in the *S. cerevisiae* proteome was determined with BLASTP

using default parameters but low-frequency filtering off [24]. *P. falciparum* proteins were classified as 'likely nuclear' if they had as a best match (E-value < 10^{-5}) a *S. cerevisiae* protein associated with the GO term nucleus in SGD and/or localised to the yeast nucleus [25].

1d. Rationales for acceptance or rejection of bioinformatics filters.

Accepted bioinformatic filters

Removal of proteins carrying putative SPs and/or predicted to be exported to the host cell removed many of the proteins directed to destinations in the endomembrane system and RBC [5,6,26]. These filters were consistent with the hypothesis that proteins with amino acid sequences encoding putative signals for trafficking to non-nuclear compartments were unlikely to represent true nuclear proteins. Proteins carrying predicted TM were considered less likely to represent nuclear proteins, despite the fact that integral membrane proteins are present in the nuclear envelope. Analysis of the *S. cerevisiae* proteome undertaken here showed that 17% (1014/5885) of all proteins contain a predicted TM compared to only 3% (57/2132) of proteins annotated as nuclear in the gene ontology [9]. Lastly, proteins found in proteomic studies of non-nuclear organelles were considered less likely to be nuclear-localised, either because they are truly associated with that organelle or because they may represent possible contaminants in organellar proteomics studies in general.

Rejected bioinformatics filters

In terms of numbers of distinct proteins, the largest subcellular destinations outside the endomembrane system apart from the nucleus are the cytosol and mitochondrion. While no commonly used predictors attempting to specifically predict cytosolic proteins in *P. falciparum* have yet emerged, *P. falciparum* mitochondrial proteins have been the target of at least four predictors [14,27-29]. Removal of proteins predicted to localsie to the mitochondrion using PlasMit [14] resulted in a very slight reduction of positive predictive value (0.1%) of the set, combined with a large loss in nuclear proteins (unfiltered set size reduced by 239 proteins or 19%). Thus, from this empirical point of view, choosing to exclude proteins based on PlasMit prediction would result in a substantially reduced set of proteins that is not further enriched in nuclear proteins.

However, the decision not to use a mitochondrial transit peptide predictor as bioinformatic filter leaves open the question of whether significant mitochondrial contamination is present in the core nuclear proteome. To address this, we looked specifically for proteins recorded as being mitochondrial in ApiLoc or as part of our literature review. 28 of these 110 proteins were found in the nuclear proteome, and 16 of these remained in the set after bioinformatic filtering. Based on this we predict 7% of the core nuclear proteome to be mitochondrial (55 proteins), and that approximately one-third of contaminating proteins are mitochondrial.

Other potential filters found to be of limited utility were based on the distribution of detected peptides across the five fractions, developmental stages, and biological replicates. Removal of proteins detected by a single peptide increased the positive predictive value by 1% only, while incurring a loss of 22% of proteins from the unfiltered set. Finally, removal of proteins that were also detected in the cytoplasmic

fraction gives an 8% increase in positive predictive value at a cost of 49% of the proteins in the set. Applying this criterion in addition to those already accepted leaves a set of 440 proteins with 81% being nuclear.

1e. Protocol for the identification of novel protein domains.

To identify novel domains in the core nuclear proteome, all proteins were assembled into a FASTA file taken from PlasmoDB v6.4. InterPro domains taken from the PlasmoDB InterPro annotation file (http://plasmodb.org/common/downloads/release6.4/Pfalciparum/PfalciparumInterpro_PlasmoDB-6.4.txt) were used to mask previously defined domains using a custom-built script (http://github.com/wwood/bbbin/blob/master/mask_eupathdb_domains.rb commit 0bbb3eb). NCBI BLAST+ 2.2.19 [30] was then used to find homology between these masked protein sequences, using the BLASTP program, a permissive E-value cutoff (1E-2) and without a low complexity filter. One-way BLAST clustering (i.e. proteins with a hit to another protein were clustered together) was implemented with a custom-built script (http://github.com/wwood/bbbin/blob/master/blast_to_blastclust.rb commit ef9278b), and inspected by interrogation of the pairwise BLAST alignment file. The majority of alignments appeared spurious, consisting mainly of the common amino acids asparagine, lysine, glutamine, glutamic acid, isoleucine and serine, and the others were inspected further. The amino acid sequences of these proteins were more rigorously confirmed not to encode any domains by using the InterProScan [31] website (http://www.ebi.ac.uk/Tools/InterProScan/) and PlasmoDB IDs at the EuPathDomains website (http://www.atgc-montpellier.fr/EuPathDomains/) that claims to be more sensitive at finding PFAM domains based on co-occurrence of particular domains [32]. However, no additional domains were found.

AP2-coincident domain (ACDC)

To further characterise the AP2-coincident domain, PSI-BLAST [24] at the NCBI website (http://blast.ncbi.nlm.nih.gov/) was used to query the nr database with default parameters starting with the conserved region found in PF11_0404 (residues 2543-2644, YYELLKTSIIICLNDILMNCIPQVFHLYKNINTSNDIKLEDILYTERKRKEQSLKYHIEYTQNSVGVSSLIPYLKLFSTEILNNVLPSAQSLEIQRLIIHSL). Successive iterations showed that the majority of hits were apicomplexan proteins annotated in the one line description as AP2 domain-containing proteins. A more rigourous workup of whether hits contained AP2 domains was carried out later in the analysis. Matching sequences were downloaded from the NCBI website in FASTA format. Sequences were then mapped to EuPathDB identifiers using a custom BioPerl [33] script (http://github.com/wwood/bbbin/blob/master/extract_from_fasta.pl commit fc1295e) by exact matching of amino acid sequences, and those not matching exactly (often rodent malaria proteins that have been the subject of recent genome-wide re-annotation) were included by using BLASTP and trusting the corresponding PlasmoDB sequence. A locally downloaded version of PSI-BLAST (2.2.24+) was then used to create a multiple sequence alignment of the domain, using residues 2668-

2869 of the *P. vivax* protein PVX_092570 (PlasmoDB version 7.0) (FAEDMFENAPDANNNGTTQVDSNREDSLGETKKIASENNSSFPMLNIDDKYYEL LKTAIIICLNDILMNSIPKLFHIYKDISTTTNVKMEDLLNDEKKRREQSVKYHIAYTQ NSIGVSSLIPYLRLFSMEILNNVLPSTQSLEIQRKIIYSLDLQAYNTSY) against the proteins downloaded from NCBI. Output was then parsed and hit sequences together with 20 amino acids either side were extracted using another custom BioPerl script (http://github.com/wwood/bbbin/blob/master/sequenceChop.pl commit 8c27d33). Hit sequences were aligned using MAFFT 6.704 using the MAFFT L-INS-i algorithm with default parameters. The resulting alignment was checked by manual inspection using JalView 2.4. The beginning and end of the alignment corresponding to likely spurious matching were removed by manual inspection. Highly similar sequences were removed since they corresponded to direct orthologs and their inclusion would bias the alignment toward these sequences unnecessarily. These highly similar sequences were from *Plasmodium, Babesia, Theileria* or *Cryptosporidium* spp. The alignment was saved in aligned FASTA format and converted to STOCKHOLM format using sreformat (part of the Ubuntu biosquid package version 1.9g+cvs20050121-2). An HMM was created using the HMMER (http://hmmer.org/) program hmmbuild version 3.0 with default parameters (Additional file 16). A protein sequence database comprising of amino acid sequences from all sequenced alveolate species was compiled. This included sequences from PlasmoDB 7.0 [34-37], ToxoDB 6.2 (strain ME49) [38], CryptoDB 4.3 [39,40], ParameciumDB v1.49 [41,42], TGD [43,44] Aug_2004 gene predictions, and *Perkinsus marinus* genome-derived GenBank [45] accession AAXJ00000000 proteins downloaded Nov 11 2010, as well as *Babesia bovis* [46], *Theileria annulata* [47] and *Theileria parva* [48] genome-derived RefSeq proteins downloaded May, 2008. The HMMER program hmmsearch was then used to search this database with default parameters, except using the -A flag to output a multiple sequence alignment that comprised of all the significant (E-value < 0.001) hits. The output STOCKHOLM format was first converted to MSF format using sreformat, and then aligned in FASTA format using JalView. This aligned FASTA format file is given as Additional file 15. To search for the AP2 domain in PF13_0114, the HMM file for PF00847 was downloaded from PFAM (v24) and searched against the PlasmoDB 7.1 version of the protein sequence using HMMER v3.0 with default parameters.

CSTF, ELM2 and MYND domains

Analysis of the other characterised protein domains was similar to the method used for the AP2-coincident domain, with slight differences. For partial CSTF, extended ELM2, and MYND domains, MAFFT version 6.833b, sequenceChop.pl commit 6a9ffda were used, except sequenceChop.pl commit 5abf5d was used for the MYND domain. For each of these three, highly similar sequences were not removed from the alignment prior to HMM creation.

PF10_0279 and PFI1600w, annotated as hypothetical and mRNA processing protein, respectively, were found to share a 56aa C terminal domain (Additional files 14 and 17). According to OrthoMCL v4 [49] PFI1600w is orthologous to the opisthokont cleavage stimulation factor (CSTF) 64kDa subunit, which is known to be involved in cleavage of 3′ untranslated regions (UTRs) [50]. PFI1600w was found in all sequenced Apicomplexans except *Theileria* spp. PF10_0279 lacks primary sequence similarity apart from the partial CSTF domain with proteins outside *Plasmodium* spp., suggesting it may be a genus-specific innovation. For the partial CSTF domain PSI-BLAST was initiated with the domain from PF10_0279, KDIPYAEEDLVKEIINEKSILQNILISKYVDMLNWTSEQVLRVLSIRKSLKRIGYNI.

A region of sequence similarity related to the ELM2 domain was found in PF11_0429 and PFE0995c (Additional files 14 and 18). ELM2 domains, first found in *Caenorhabditis elegans* [51] were subsequently found in a number of *Drosophila melanogaster* and vertebrate proteins involved in transcriptional repression through association with histone deacetylases and methyltransferases, and have been localised to the nucleus [52-54]. This domain had recently been annotated as an ELM2 domain in PlasmoDB version 7.0. However, the regions of similarity between the two proteins extended approx. six and 50 residues beyond the N and C terminal boundaries, respectively (PROSITE, domain PS51156) [55]. As such, we refer to this region of sequence similarity as an “extended ELM2 domain”. PF11_0429 and some apicomplexan homologues also carry annotated PHD-finger domains that are involved in chromatin remodelling [56]. For the extended ELM2 domain PSI-BLAST was initiated with the domain from PF11_0429, KKKDKVKQKSEGSKYTNQINVGENYQVSNVSTFFLNSHSEKYDETSKSELVYSPYL
LERMKENYLSEGQYELVIKNDYELAIFIKELAKNWKCQLGWHPFTPEYAFKILHH
VDYNPKKAIELLKSSEFNFLEICDPPIRKYENKWRPRDKRGQSDSPYPSSELLQSYLK
R, and 50 amino acids either side of the domain were aligned. The final alignment was created using MAFFT L-INS-i instead of hmmsearch.

The algorithm also found a domain conserved between PF14_0310 (conserved *Plasmodium* protein) and PF10_0150 (methionine aminopeptidase 1b, putative) (Additional files 14 and 19). Subsequent characterisation with PSI-BLAST [24] and the HMM-HMM comparison tool HHSearch [57] revealed homology to the MYND zinc finger domain (PFAM ID PF01753), which is found in many eukaryote proteomes. Further searching on full amino acid sequences (no longer masked by EuPathDB-annotated domains) from all *P. falciparum* proteins identified three additional proteins (PFF0350w, PFF0105w, PF13_0293) each of which carried a previously annotated MYND domain. PF13_0293 encodes a putative histone-lysine N-methyltransferase and has previously been localised to the nucleus [58]. For the MYND domain, PSI-BLAST was initiated with the domain from PF10_0150, LCSGCKKVLIKKLSCPICLKNKIFSYFCNQECFKGSWKEHQKIHENMNKENNEKE

DH. Newly defined protein domains will be submitted to PFAM upon this article's acceptance into a journal (Additional files 15 and 17-19).

In addition, our search also identified similarity between five alveolin proteins [59]. Two of these have been localised to the cytoskeleton [59,60] in *P. falciparum*, and all five have been localised to the parasite cytoskeleton in *T. gondii* [61]. It is possible that these proteins are peripherally involved in nuclear biology as part of the cytoskeleton, which has been implicated in the import of proteins into the nucleus [62], though it seems most probable that they are simply contaminants in the nuclear fractions.

1f. Protocol for identification of lineage-specific proteins in the core nuclear proteome.

Characterisation of Lineage-Specificity

To determine how many proteins in the core nuclear proteome were phylum- or genus-specific, OrthoMCL version 4 was used. The definition of phylum-specific was simply that the gene had no orthologue outside of Apicomplexa, where all genes in the OrthoMCL group were considered the complete set of orthologous genes. Likewise, genus-specific genes had no orthologues ouside *Plasmodium* spp. Specifically, a custom ruby script https://github.com/wwood/bbbin/blob/master/orthomcl_species_jumper.rb commit abf2c13 was used with the arguments "-i pfal -o pfal,pviv,bbov,pber,ncan,tann,tgon,pyoe,pcha,pkno,tpar,cmur,chom,cpar -v" and "-i pfal -o pfal,pviv,pber,pyoe,pcha,pkno -v" to search for orthologues outside the phylum and genus, respectively. Each line of the outputs were then truncated to 100 characters for computer performance reasons, and then pasted into a LibreOffice spreadsheet (http://www.libreoffice.org/), and saved in tab-separated values format. Taxon-specificity was then interrogated using spreadsheet formula, and summarised using GNU awk and "uniq -c" (http://www.gnu.org/).

References

1. Gatlin CL, Eng JK, Cross ST, Detter JC, Yates JR, III: **Automated identification of amino acid sequence variations in proteins by HPLC/microspray tandem mass spectrometry.** *Anal Chem* 2000, **72:** 757-763.
2. Beissbarth T, Speed TP: **GOstat: find statistically overrepresented Gene Ontologies within a group of genes.** *Bioinformatics* 2004, **20:** 1464-1465.
3. Huang da W, Sherman BT, Lempicki RA: **Systematic and integrative analysis of large gene lists using DAVID bioinformatics resources.** *Nat Protoc* 2009, **4:** 44-57.
4. van Ooij C., Tamez P, Bhattacharjee S, Hiller NL, Harrison T, Liolios K *et al*.: **The malaria secretome: from algorithms to essential function in blood stage infection.** *PLoS Pathog* 2008, **4:** e1000084.
5. Sargeant TJ, Marti M, Caler E, Carlton JM, Simpson K, Speed TP *et al*.: **Lineage-specific expansion of proteins exported to erythrocytes in malaria parasites.** *Genome Biol* 2006, **7:** R12.
6. Flueck C, Bartfai R, Volz J, Niederwieser I, Salcedo-Amaya AM, Alako BT *et al*.: **Plasmodium falciparum heterochromatin protein 1 marks genomic loci linked to phenotypic variation of exported virulence factors.** *PLoS Pathog* 2009, **5:** e1000569.
7. Bendtsen JDv, Nielsen H, von Heijne G, Brunak SÃ: **Improved prediction of signal peptides: SignalP 3.0.** *J Mol Biol* 2004, **340:** 783-795.
8. Krogh A, Larsson B, von Heijne G, Sonnhammer EL: **Predicting transmembrane protein topology with a hidden Markov model: application to complete genomes.** *J Mol Biol* 2001, **305:** 567-580.
9. Ashburner M, Ball CA, Blake JA, Botstein D, Butler H, Cherry JM *et al*.: **Gene ontology: tool for the unification of biology. The Gene Ontology Consortium.** *Nature genetics* 2000, **25:** 25-29.
10. Cherry JM, Adler C, Ball C, Chervitz SA, Dwight SS, Hester ET *et al*.: **SGD: Saccharomyces Genome Database.** *Nucleic Acids Res* 1998, **26:** 73-79.
11. Goto N, Prins P, Nakao M, Bonnal R, Aerts J, Katayama T: **BioRuby: bioinformatics software for the Ruby programming language.** *Bioinformatics* 2010, **26:** 2617-2619.
12. Lamarque M, Tastet C, Poncet J, Demettre E, Jouin P, Vial H *et al*.: **Food vacuole proteome of the malarial parasite Plasmodium falciparum.** *Proteomics Clin Appl* 2008, **2:** 1361-1374.
13. Vincensini L, Richert S, Blisnick T, Van DA, Leize-Wagner E, Rabilloud T *et al*.: **Proteomic analysis identifies novel proteins of the Maurer's clefts, a secretory compartment delivering Plasmodium falciparum proteins to the surface of its host cell.** *Mol Cell Proteomics* 2005, **4:** 582-593.
14. Bender A, van Dooren GG, Ralph SA, McFadden GI, Schneider G: **Properties and prediction of mitochondrial transit peptides from Plasmodium falciparum.** *Mol Biochem Parasitol* 2003, **132:** 59-66.
15. Mahajan B, Selvapandiyan A, Gerald NJ, Majam V, Zheng H, Wickramarachchi T *et al*.: **Centrins, cell cycle regulation proteins in human malaria parasite Plasmodium falciparum.** *J Biol Chem* 2008, **283:** 31871-31883.
16. Richard D, Bartfai R, Volz J, Ralph SA, Muller S, Stunnenberg HG *et al*.: **A genome-wide chromatin-associated nuclear peroxiredoxin from the malaria parasite Plasmodium falciparum.** *J Biol Chem* 2011, **286:** 11746-11755.
17. Cokol M, Nair R, Rost B: **Finding nuclear localization signals.** *EMBO Rep* 2000, **1:** 411-415.
18. Nguyen Ba AN, Pogoutse A, Provart N, Moses AM: **NLStradamus: a simple Hidden Markov Model for nuclear localization signal prediction.** *BMC Bioinformatics* 2009, **10:** 202.
19. Kosugi S, Hasebe M, Tomita M, Yanagawa H: **Systematic identification of cell cycle-dependent yeast nucleocytoplasmic shuttling proteins by prediction of composite motifs.** *Proc Natl Acad Sci U S A* 2009, **106:** 10171-10176.

20. Llinas M, Bozdech Z, Wong ED, Adai AT, DeRisi JL: **Comparative whole genome transcriptome analysis of three Plasmodium falciparum strains.** *Nucleic Acids Res* 2006, **34:** 1166-1173.
21. Wickham H: *ggplot2: elegant graphics for data analysis*. Springer-Verlag New York Inc; 2009.
22. Bischoff E, Vaquero C: **In silico and biological survey of transcription-associated proteins implicated in the transcriptional machinery during the erythrocytic development of Plasmodium falciparum.** *BMC Genomics* 2010, **15:** 34.
23. Le Roch KG, Zhou Y, Blair PL, Grainger M, Moch JK, Haynes JD *et al*.: **Discovery of gene function by expression profiling of the malaria parasite life cycle.** *Science* 2003, **301:** 1503-1508.
24. Altschul SF, Madden TL, Schaffer AA, Zhang J, Zhang Z, Miller W *et al*.: **Gapped BLAST and PSI-BLAST: a new generation of protein database search programs.** *Nucleic Acids Res* 1997, **25:** 3389-3402.
25. Huh WK, Falvo JV, Gerke LC, Carroll AS, Howson RW, Weissman JS *et al*.: **Global analysis of protein localization in budding yeast.** *Nature* 2003, **425:** 686-691.
26. Tonkin CJ, Pearce JA, McFadden GI, Cowman AF: **Protein targeting to destinations of the secretory pathway in the malaria parasite Plasmodium falciparum.** *Curr Opin Microbiol* 2006, **9:** 381-387.
27. Verma R, Varshney GC, Raghava GP: **Prediction of mitochondrial proteins of malaria parasite using split amino acid composition and PSSM profile.** *Amino Acids* 2010, **39:** 101-110.
28. Chen YL, Li QZ, Zhang LQ: **Using increment of diversity to predict mitochondrial proteins of malaria parasite: integrating pseudo-amino acid composition and structural alphabet.** *Amino Acids* 2010, [epub ahead of print].
29. Jia C, Liu T, Chang AK, Zhai Y: **Prediction of mitochondrial proteins of malaria parasite using bi-profile Bayes feature extraction.** *Biochimie* 2011, **93:** 778-782.
30. Camacho C, Coulouris G, Avagyan V, Ma N, Papadopoulos J, Bealer K *et al*.: **BLAST+: architecture and applications.** *BMC Bioinformatics* 2009, **10:** 421.
31. Quevillon E, Silventoinen V, Pillai S, Harte N, Mulder N, Apweiler R *et al*.: **InterProScan: protein domains identifier.** *Nucleic Acids Res* 2005, **33:** W116.
32. Ghouila A, Terrapon N, Gascuel O, Guerfali FZ, Laouini D, Marechal E *et al*.: **EuPathDomains: The divergent domain database for eukaryotic pathogens.** *Infect Genet Evol* 2010, **11:** 698-707.
33. Stajich JE, Block D, Boulez K, Brenner SE, Chervitz SA, Dagdigian C *et al*.: **The Bioperl toolkit: Perl modules for the life sciences.** *Genome Res* 2002, **12:** 1611-1618.
34. Carlton JM, Adams JH, Silva JC, Bidwell SL, Lorenzi H, Caler E *et al*.: **Comparative genomics of the neglected human malaria parasite Plasmodium vivax.** *Nature* 2008, **455:** 757-763.
35. Carlton JM, Angiuoli SV, Suh BB, Kooij TW, Pertea M, Silva JC *et al*.: **Genome sequence and comparative analysis of the model rodent malaria parasite Plasmodium yoelii yoelii.** *Nature* 2002, **419:** 512-519.
36. Hall N, Karras M, Raine JD, Carlton JM, Kooij TW, Berriman M *et al*.: **A comprehensive survey of the Plasmodium life cycle by genomic, transcriptomic, and proteomic analyses.** *Science* 2005, **307:** 82-86.
37. Pain A, Bohme U, Berry AE, Mungall K, Finn RD, Jackson AP *et al*.: **The genome of the simian and human malaria parasite Plasmodium knowlesi.** *Nature* 2008, **455:** 799-803.
38. Kissinger JC, Gajria B, Li L, Paulsen IT, Roos DS: **ToxoDB: accessing the Toxoplasma gondii genome.** *Nucleic Acids Res* 2003, **31:** 234-236.
39. Heiges M, Wang H, Robinson E, Aurrecoechea C, Gao X, Kaluskar N *et al*.: **CryptoDB: a Cryptosporidium bioinformatics resource update.** *Nucleic Acids Res* 2006, **34:** D419.
40. Abrahamsen MS, Templeton TJ, Enomoto S, Abrahante JE, Zhu G, Lancto CA *et al*.: **Complete genome sequence of the apicomplexan, Cryptosporidium parvum.** *Science* 2004, **304:** 441-445.
41. Arnaiz O, Cain S, Cohen J, Sperling L: **ParameciumDB: a community resource that integrates the Paramecium tetraurelia genome sequence with genetic data.** *Nucleic Acids Res* 2006, **35:** D439.

42. Aury JM, Jaillon O, Duret L, Noel B, Jubin C, Porcel BM *et al*.: **Global trends of whole-genome duplications revealed by the ciliate Paramecium tetraurelia.** *Nature* 2006, **444:** 171-178.
43. Stover NA, Krieger CJ, Binkley G, Dong Q, Fisk DG, Nash R *et al*.: **Tetrahymena Genome Database (TGD): a new genomic resource for Tetrahymena thermophila research.** *Nucleic Acids Res* 2006, **34:** D500.
44. Eisen JA, Coyne RS, Wu M, Wu D, Thiagarajan M, Wortman JR *et al*.: **Macronuclear genome sequence of the ciliate Tetrahymena thermophila, a model eukaryote.** *PLoS Biol* 2006, **4:** e286.
45. Benson DA, Boguski MS, Lipman DJ, Ostell J, Ouellette BF: **GenBank.** *Nucleic Acids Res* 1998, **26:** 1.
46. Brayton KA, Lau AO, Herndon DR, Hannick L, Kappmeyer LS, Berens SJ *et al*.: **Genome sequence of Babesia bovis and comparative analysis of apicomplexan hemoprotozoa.** *PLoS pathogens* 2007, **3:** 1401-1413.
47. Pain A, Renauld H, Berriman M, Murphy L, Yeats CA, Weir W *et al*.: **Genome of the host-cell transforming parasite Theileria annulata compared with T. parva.** *Science* 2005, **309:** 131-133.
48. Gardner MJ, Bishop R, Shah T, De Villiers EP, Carlton JM, Hall N *et al*.: **Genome sequence of Theileria parva, a bovine pathogen that transforms lymphocytes.** *Science* 2005, **309:** 134-137.
49. Li L, Stoeckert CJ, Jr., Roos DS: **OrthoMCL: identification of ortholog groups for eukaryotic genomes.** *Genome Res* 2003, **13:** 2178-2189.
50. Takagaki Y, Manley JL, MacDonald CC, Wilusz J, Shenk T: **A multisubunit factor, CstF, is required for polyadenylation of mammalian pre-mRNAs.** *Genes Dev* 1990, **4:** 2112-2120.
51. Solari F, Bateman A, Ahringer J: **The Caenorhabditis elegans genes egl-27 and egr-1 are similar to MTA1, a member of a chromatin regulatory complex, and are redundantly required for embryonic patterning.** *Development* 1999, **126:** 2483-2494.
52. Wang L, Charroux B, Kerridge S, Tsai CC: **Atrophin recruits HDAC1/2 and G9a to modify histone H3K9 and to determine cell fates.** *EMBO Rep* 2008, **9:** 555-562.
53. Wang L, Rajan H, Pitman JL, McKeown M, Tsai CC: **Histone deacetylase-associating Atrophin proteins are nuclear receptor corepressors.** *Genes Dev* 2006, **20:** 525-530.
54. Ding Z, Gillespie LL, Paterno GD: **Human MI-ER1 alpha and beta function as transcriptional repressors by recruitment of histone deacetylase 1 to their conserved ELM2 domain.** *Mol Cell Biol* 2003, **23:** 250-258.
55. Hulo N, Bairoch A, Bulliard V, Cerutti L, Cuche BA, de Castro E *et al*.: **The 20 years of PROSITE.** *Nucleic Acids Res* 2008, **36:** D245-D249.
56. Musselman CA, Kutateladze TG: **PHD fingers: epigenetic effectors and potential drug targets.** *Mol Interv* 2009, **9:** 314-323.
57. Soding J, Biegert A, Lupas AN: **The HHpred interactive server for protein homology detection and structure prediction.** *Nucleic Acids Res* 2005, **33:** W244-W248.
58. Volz J, Carvalho TG, Ralph SA, Gilson P, Thompson J, Tonkin CJ *et al*.: **Potential epigenetic regulatory proteins localise to distinct nuclear sub-compartments in Plasmodium falciparum.** *Int J Parasitol* 2010, **40:** 109-121.
59. Gould SB, Tham WH, Cowman AF, McFadden GI, Waller RF: **Alveolins, a new family of cortical proteins that define the protist infrakingdom Alveolata.** 2008, **25:** 1219.
60. Hu G, Cabrera A, Kono M, Mok S, Chaal BK, Haase S *et al*.: **Transcriptional profiling of growth perturbations of the human malaria parasite Plasmodium falciparum.** *Nat Biotechnol* 2010, **28:** 91-98.
61. Anderson-White BR, Ivey FD, Cheng K, Szatanek T, Lorestani A, Beckers CJ *et al*.: **A family of intermediate filament-like proteins is sequentially assembled into the cytoskeleton of Toxoplasma gondii.** *Cell Microbiol* 2011, **13:** 18-31.
62. Wagstaff KM, Jans DA: **Importins and beyond: non-conventional nuclear transport mechanisms.** *Traffic* 2009, **10:** 1188-1198.
