# Supplementary material for: Organellar proteomics reveals hundreds of novel nuclear proteins in the malaria parasite Plasmodium falciparum
Source: Genome Biol. 2012 Nov 26;13(11):R108. doi: 10.1186/gb-2012-13-11-r108 (PMC4053738; doi:10.1186/gb-2012-13-11-r108)
Supplement: Additional file 2 — Summary table of tryptic peptides measured by MudPIT and Spearman rank correlations between replicates. [file gb-2012-13-11-r108-S2.PDF]

Additional file 2_Oehring et al.

(a)

A	B	C	D	E	F	G	H	I	J
stage	compartment	fraction	replicate	# unique peptides	FDR (%)	# proteins	total # proteins	# unique proteins	# proteins in nuclear fractions
**rings**	cytoplasm	1	A	1797	0.11	303	1050	181	896
	cytoplasm	1	B	3852	0.13	538			
	nuclear	2	A	649	0.15	167			
		2	B	1003	0.10	164			
		3	A	679	0.59	117			
		3	B	456	0.00	49			
		4	A	657	0.30	63			
		4	B	2042	0.29	253			
		5	A	1816	0.28	318			
		5	B	3421	0.06	565			
**trophozoites**	cytoplasm	1	A	2024	0.05	347	1017	159	901
	cytoplasm	1	B	3611	0.00	493			
	nuclear	2	A	1416	0.71	315			
		2	B	2093	0.19	309			
		3	A	811	0.86	156			
		3	B	2821	0.18	309			
		4	A	1198	0.42	125			
		4	B	2583	0.12	227			
		5	A	1620	0.19	149			
		5	B	3590	0.14	350			
**schizonts**	cytoplasm	1	A	2331	0.47	401	1092	186	948
	cytoplasm	1	B	3675	0.22	513			
	nuclear	2	A	576	0.00	162			
		2	B	406	0.25	117			
		3	A	865	0.12	176			
		3	B	1480	0.27	197			
		4	A	931	0.86	91			
		4	B	1497	0.47	199			
		5	A	1811	0.39	267			
		5	B	2945	0.37	435			
**TOTAL**							**1518**	**526**	**1273**

(b)

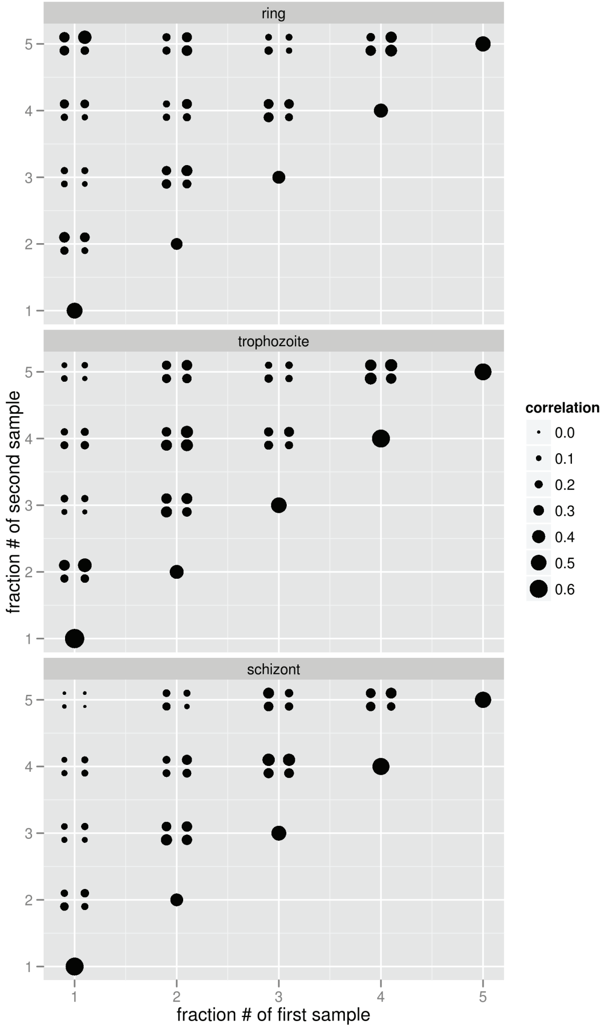

Additional file 2. (a) Summary table of tryptic peptides measured by MudPIT. Column A: parasite stage. Column B: cellular compartment. Column C: protein fractions 1 to 5. Column D replicates A and B. Column E: number of unique peptides detected. Column F: false discovery rate. Column G: number of different proteins detected. Column H: total number of proteins detected per developmental stage. Column I: total number of proteins detected exclusively in one developmental stage. Column J: total number of proteins detected in the nuclear fractions 2 to 5 per developmental stage. (b) Spearman rank correlations between replicates and across fractions. The size of each dot represents the Spearman coefficient between two samples. Dots on the diagonal depict the correlation between biological replicates, and off-diagonal dots represent the correlations between two samples taken from different fractions.
