# Supplementary material for: Organellar proteomics reveals hundreds of novel nuclear proteins in the malaria parasite Plasmodium falciparum
Source: Genome Biol. 2012 Nov 26;13(11):R108. doi: 10.1186/gb-2012-13-11-r108 (PMC4053738; doi:10.1186/gb-2012-13-11-r108)
Supplement: Additional file 7 — Enrichment analyses for KEGG pathways and GO terms in proteins found only in the combined nuclear or cytoplasmic fractions. [file gb-2012-13-11-r108-S7.PDF]

Additional file 7_Oehring et al.

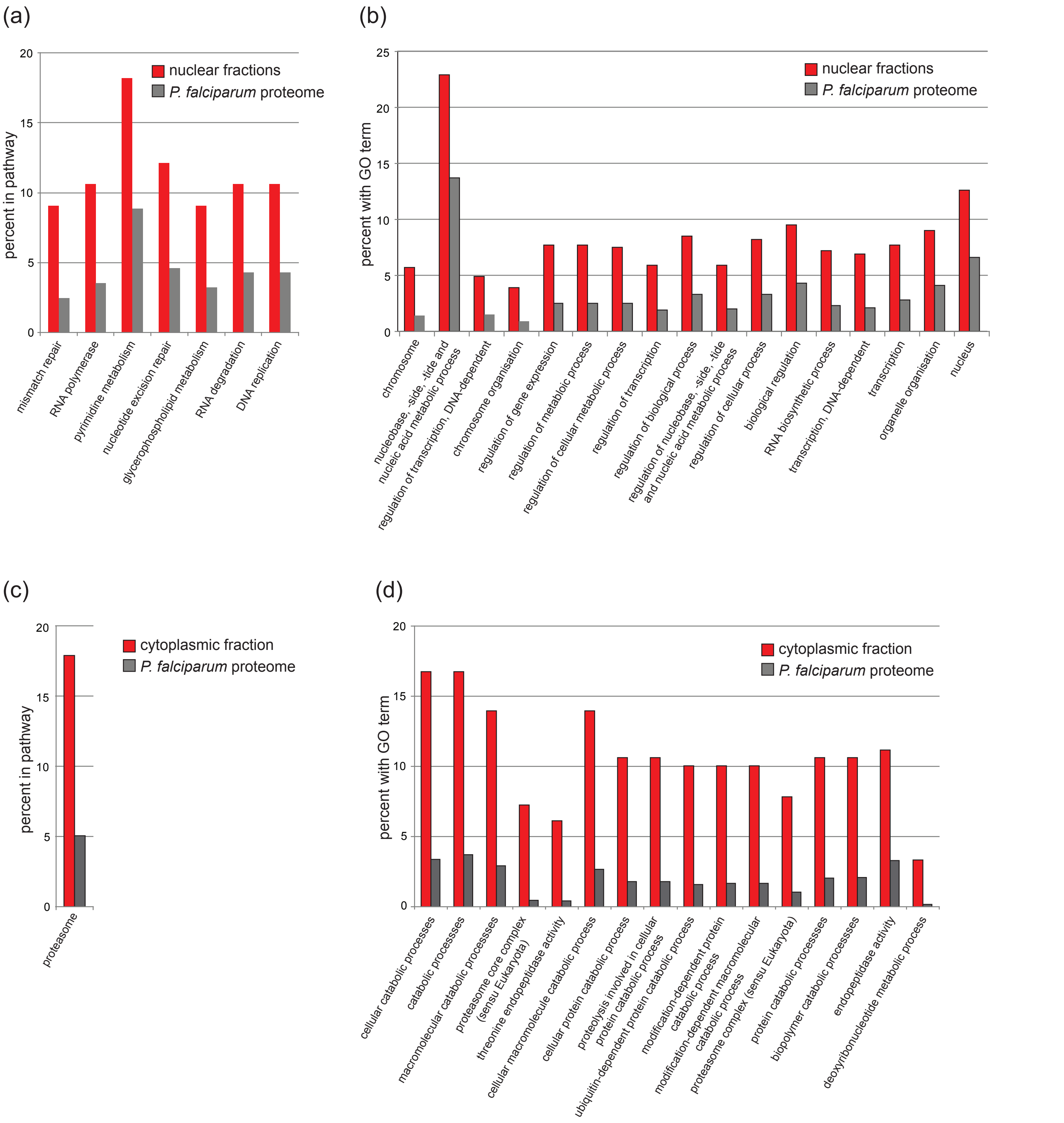

Additional file 7. Enrichment analyses for KEGG pathways and GO terms. Proteins found only in the nuclear preparation (fractions 2 to 5) or only in the cytoplasmic fraction were subject to enrichment analyses. (a) DAVID [1] was used to test for KEGG (Kyoto Encyclopedia of Genes and Genomes) pathway enrichment ($p<0.05$) in the nuclear fractions. Enriched metabolic pathways included DNA replication, mismatch repair, RNA polymerase, pyrimidine metabolism, and glycerophospholipid metabolism. (b) GOstat [2] was used to test for GO term enrichment (p<10-5) in the nuclear fractions. Enriched GO terms included nucleus, chromosome, and transcription. Percentages of nuclear proteins with relevant pathways and terms are shown in red, the percentages of the whole proteome with the corresponding pathways and terms are shown in grey. (c) Enrichment in KEGG annotations for proteins in the cytoplasmic fraction compared to the background frequency in the entire proteome ($p<0.05$). (d) Enrichment in GO terms for proteins in the cytoplasmic fraction compared to the background frequency in the entire proteome (p<10-5). Percentages of cytoplasmic proteins with relevant pathways and terms are shown in red, the percentages of the whole proteome with the corresponding pathways and terms are shown in grey.

1. Huang da W, Sherman BT, Lempicki RA: Systematic and integrative analysis of large gene lists using DAVID bioinformatics resources. Nat Protoc 2009, 4: 44-57.
2. Beissbarth T, Speed TP: GOstat: find statistically overrepresented Gene Ontologies within a group of genes. Bioinformatics 2004, 20: 1464-1465.
