# Supplementary material for: Organellar proteomics reveals hundreds of novel nuclear proteins in the malaria parasite Plasmodium falciparum
Source: Genome Biol. 2012 Nov 26;13(11):R108. doi: 10.1186/gb-2012-13-11-r108 (PMC4053738; doi:10.1186/gb-2012-13-11-r108)
Supplement: Additional file 11 — List of proteins tagged and localized in this study. [file gb-2012-13-11-r108-S11.PDF]

Additional file 11. List of proteins tagged and localised in this study. Columns J to X list the number of unique tryptic peptides (derived from either replicate A or B, whichever displayed the higher number of unique peptides) measured for each protein in each IDC stage. White/grey cells, 1 to 2 peptides detected; beige cells, 3 to 5 peptides detected; orange cells, 6 to 9 peptides detected; dark orange cells, greater than 10 peptides detected; R, ring stages; T, trophozoites; S, schizonts.

A	B	C	D	E	F	G	H	I	J	K	L	M	N	O	P	Q	R	S	T	U	V	W	X
									fraction 1			fraction 2			fraction 3			fraction 4			fraction 5		
**Acronym**	**Gene ID**	**Product annotation at time of selection**	**PlasmoDB release**	**Product annotation PlasmoDB version8.0**	**MW (kDa)**	**TM**	**SP**	**PEXEL**	**R**	**T**	**S**	**R**	**T**	**S**	**R**	**T**	**S**	**R**	**T**	**S**	**R**	**T**	**S**
NuProC1	MAL7P1.38	regulator of chromosome condensation, putative	5.5	regulator of chromosome condensation, putative	79	-	-	-					2		4	28	8	5	15	7	1		7
NuProC2	PF10_0278	BRIX domain containing protein, putative	5.5	nucleolar preribosomal assembly protein, putative	57.1	-	-	-					2					1					
NuProC3	PF11_0250	high mobility group-like protein NHP2, putative	5.4	splicing factor, putative	16	-	-	-	1	2	1							2	4	3	1		
NuProC4	PF11_0293	multiprotein bridging factor type 1, putative	5.0	multiprotein bridging factor type 1, putative	15.5	-	-	-		1		10	7	3		2	6						
NuProC5	PF13_0042	fork head domain protein, putative	5.4	fork head domain protein, putative	68.3	-	-	-											2				
NuProC6	PFL0635c	bromodomain protein, putative	5.5	bromodomain protein, putative	125.5	-	-	-								1	1	3	10	4	1	7	5
NuProc7	PF14_0393	structure-specific recognition protein, putative	5.5	structure specific recognition protein, putative	58.8	-	-	-		1	2							10	20	11	3		6
NuProC8	PF08_0083	hypothetical protein, conserved	5.5	conserved Plasmodium protein, unknown function	70.7	-	-	-											1		1	1	1
NuProC9	PF10_0091	hypothetical protein	5.4	zinc finger protein, putative	41.3	-	-	-									2						
NuProC10	PF10_0328	hypothetical protein	5.0	bromodomain protein, putative	56	-	-	-						2		4	2	3	11	2	4	7	4
NuProC11	PF11_0254	hypothetical protein	5.0	conserved Plasmodium protein, unknown function	62.3	-	-	-										1	7	1		5	3
NuProC12	PF11_0332	hypothetical protein	6.4	nucleic acid binding protein, putative	31.7	-	-	-		1					1	3		5					
NuProC13	PF13_0099	hypothetical protein	5.0	conserved Plasmodium protein, unknown function	23.2	-	-	-							1	5	5		1	1			
NuProC14	PF14_0176	hypothetical protein	6.4	conserved Plasmodium protein, unknown function	26.7	-	-	-								8	4		2				1
NuProC15	PF14_0433	hypothetical protein	5.5	erythrocyte membrane-like protein	69.4	-	-	-		1			1			2							
NuProC16	PFC0126c	hypothetical protein	5.0	PFMNL-1 CISD1-like iron-sulfur protein, putative	18.4	-	-	-									2						
NuProC17	PFC0130c	hypothetical protein, conserved	5.5	conserved Plasmodium protein, unknown function	48.1	-	-	-	1							3					1	1	
NuProC18	PFC0690c	hypothetical protein, conserved	5.5	conserved Plasmodium protein, unknown function	42	-	-	-					2			3	2						
NuProC19	PFI0610w	hypothetical protein	5.0	conserved Plasmodium protein, unknown function	33.2	-	-	-				1			4	15	10			4			
NuProC20	PFI1355w	hypothetical protein, conserved	5.5	conserved Plasmodium protein, unknown function	70	-	-	-				2	3			6	11			2	1		
NuProC21	PFL0185c	hypothetical protein, conserved	5.5	nucleosome assembly protein	40.5	-	-	-	9	8	9		5			4		10	7	7	3		
NuProC22	PFL0450c	hypothetical protein	6.4	conserved Plasmodium protein, unknown function	20	-	-	-								1	2						
n-NuProc1	PF11_0099	heat shock protein DNAj homologue Pfj2	5.5	heat shock protein DnaJ homologue Pfj2	62.4	1	1	1	1	4	5				1	6	1	2	5	1	2		5
n-NuProc2	MAL7P1.77	hypothetical protein, conserved	5.4	conserved Plasmodium protein, unknown function	60.6	-	1	-			1		4								3	1	3
n-NuProc3	PF07_0007	hypothetical protein, conserved	5.5	conserved Plasmodium protein, unknown function	14.2	1	-	-	1		1		1		1	2	1						
n-NuProc4	PF10_0100	hypothetical protein	5.5	conserved Plasmodium protein, unknown function	13.7	2	-	-					2		2	1		1	1		3	2	1
n-NuProc5	PF11_0179	hypothetical protein	5.5	conserved Plasmodium protein, unknown function	15.3	3	1	-					2		2	2					2	2	1
n-NuProc6	PFB0395w	hypothetical protein	5.5	conserved Plasmodium protein, unknown function	28.2	1	1	-							2	1	1				3		
