# Supplementary material for: Organellar proteomics reveals hundreds of novel nuclear proteins in the malaria parasite Plasmodium falciparum
Source: Genome Biol. 2012 Nov 26;13(11):R108. doi: 10.1186/gb-2012-13-11-r108 (PMC4053738; doi:10.1186/gb-2012-13-11-r108)
Supplement: Additional file 12 — Detailed IFA localization of NuProCs 1 to 22 during the IDC. [file gb-2012-13-11-r108-S12.PDF]

Additional file 12_Oehring et al.
Detailed IFA analysis of NuProCs 1-22 during the IDC.

NuProC1 (MAL7P1.38)

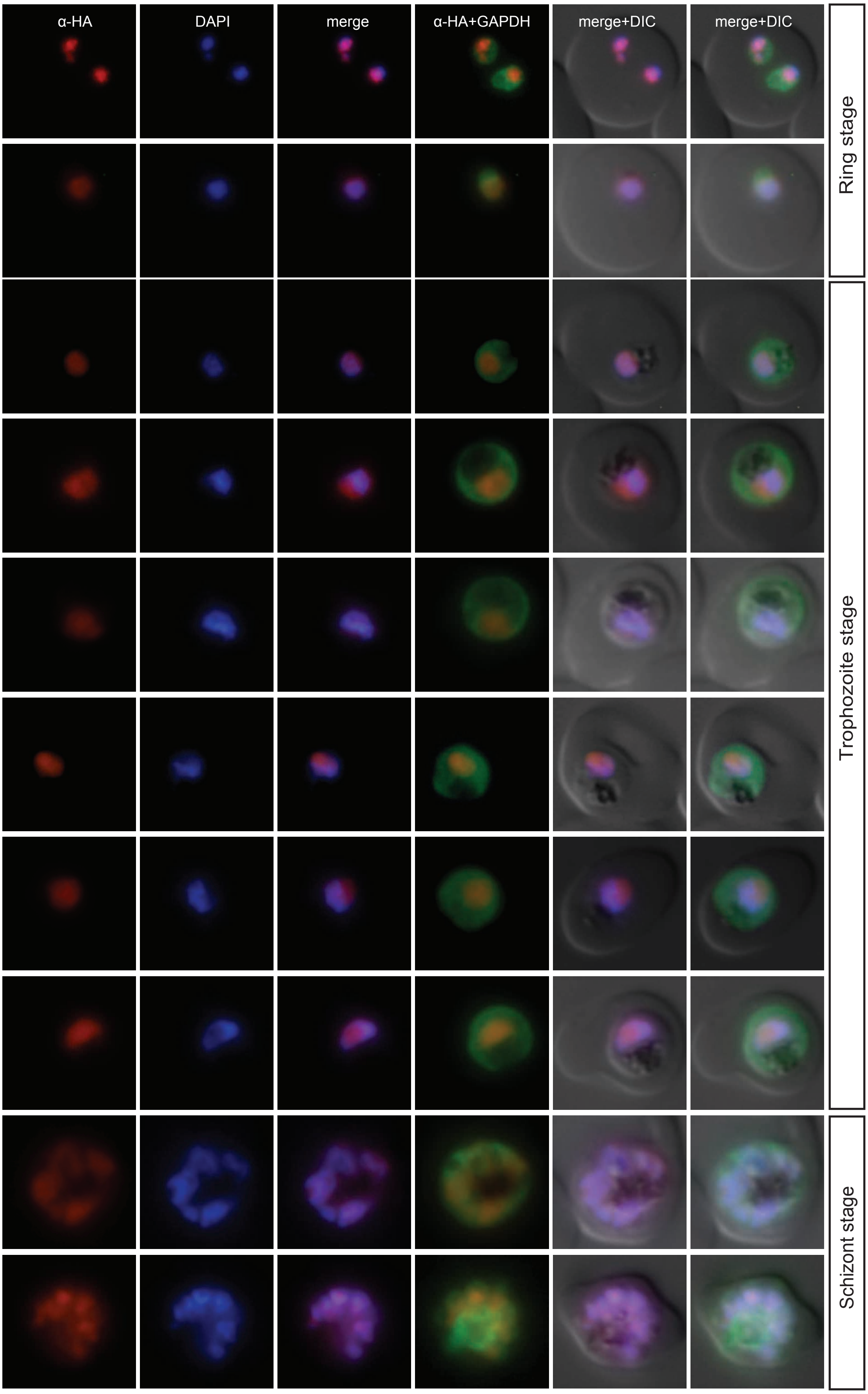

Localisation of NuProC1-3xHA (MAL7P1.38) during the IDC. Localisation of the tagged protein was visualised using anti-HA antibodies (red). Antibodies against GAPDH were used to visualise the cytosolic compartment. DAPI was used to visualise the nucleus. DIC images are shown as reference.

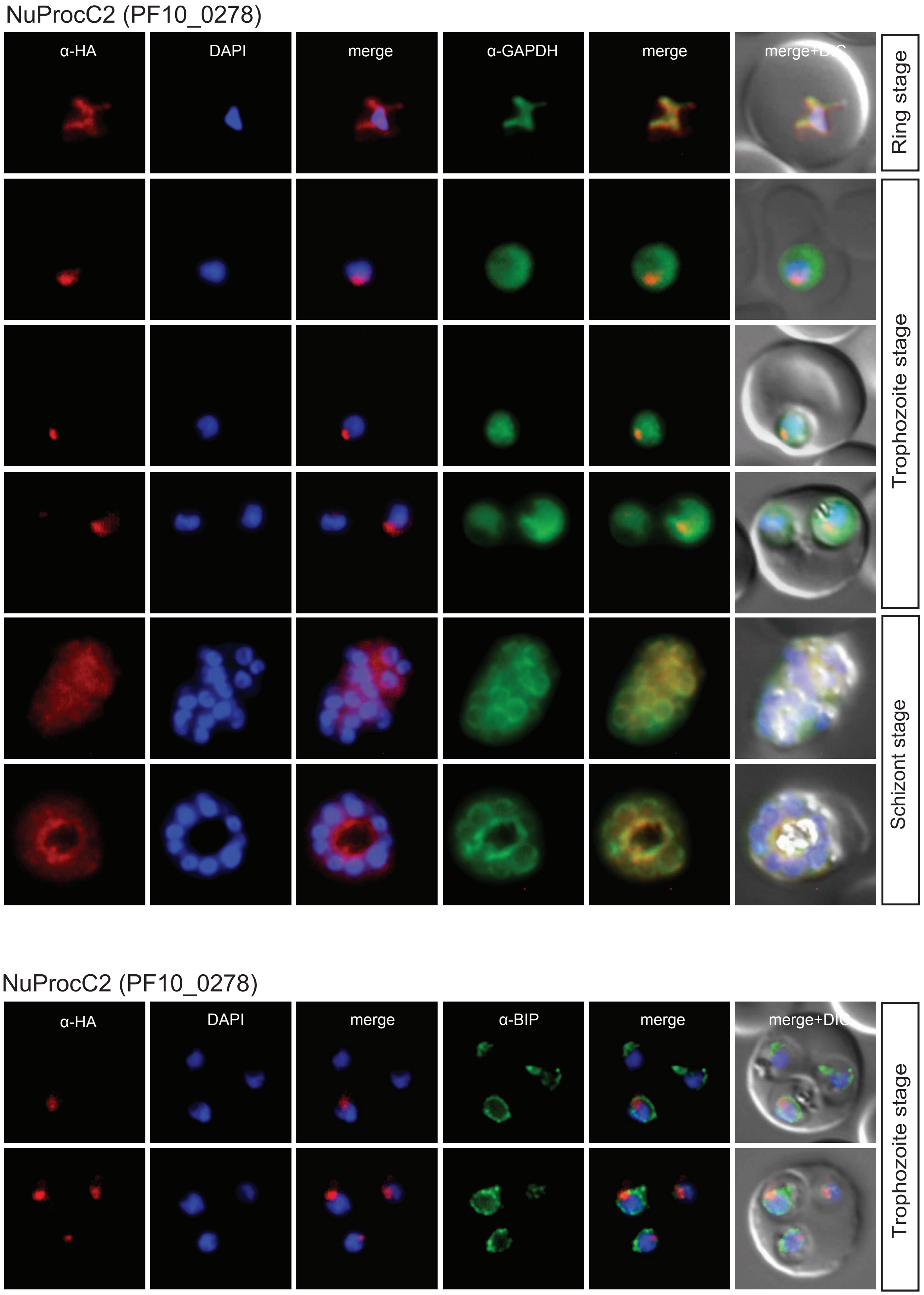

Localisation of NuProC2-3xHA (PF10_0278) during the IDC. Localisation of the tagged protein was visualised using anti-HA antibodies (red). Antibodies against GAPDH and PfBIP were used to visualise the cytosolic (top panel) and ER (bottom panel) compartments, respectively. DAPI was used to visualise the nucleus. DIC images are shown as reference.

NuProC3 (PF11_0250)

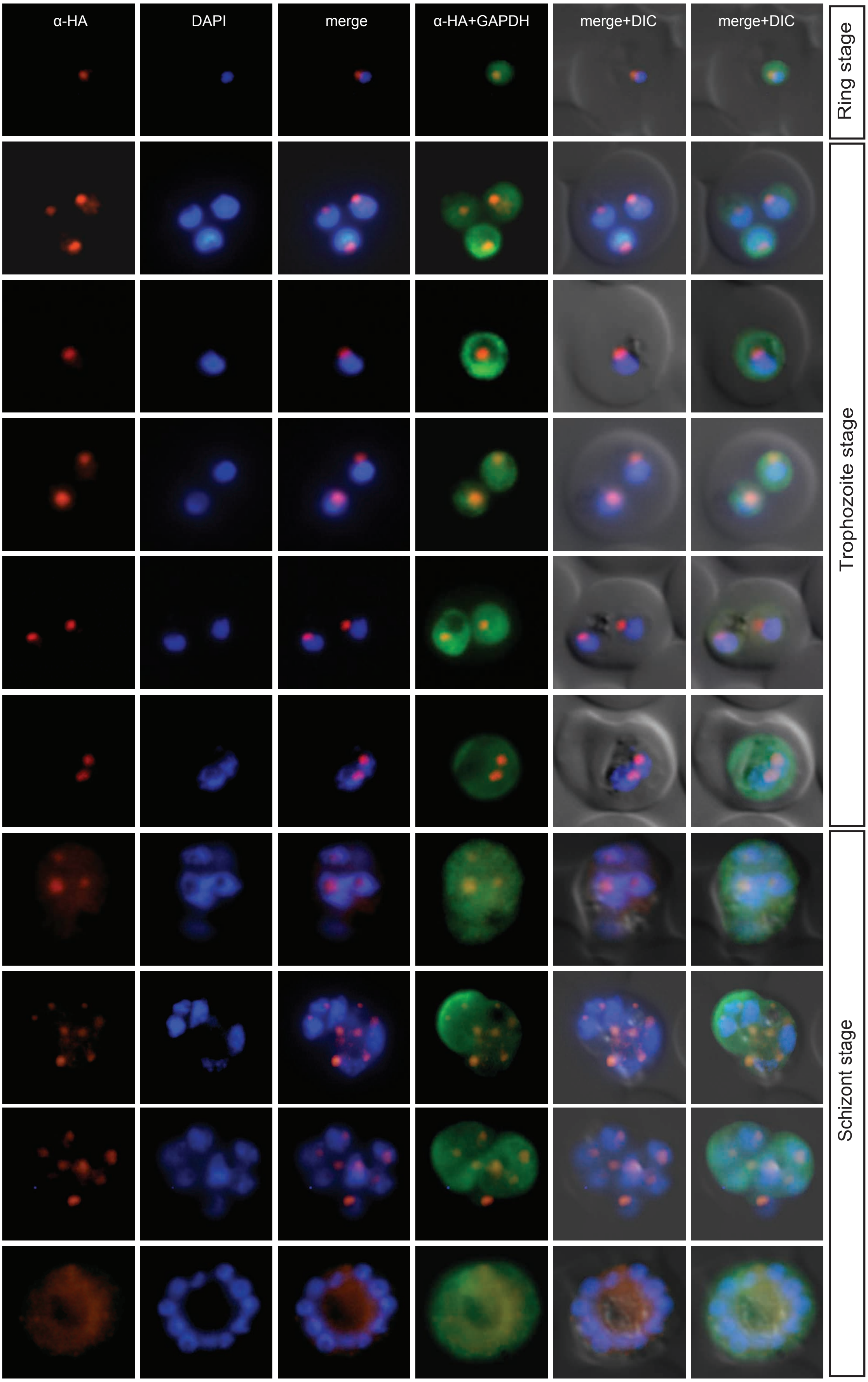

Localisation of NuProC3-3xHA (PF11_0250) during the IDC. Localisation of the tagged protein was visualised using anti-HA antibodies (red). Antibodies against GAPDH were used to visualise the cytosolic compartment. DAPI was used to visualise the nucleus. DIC images are shown as reference.

NuProC4 (PF11_0293)

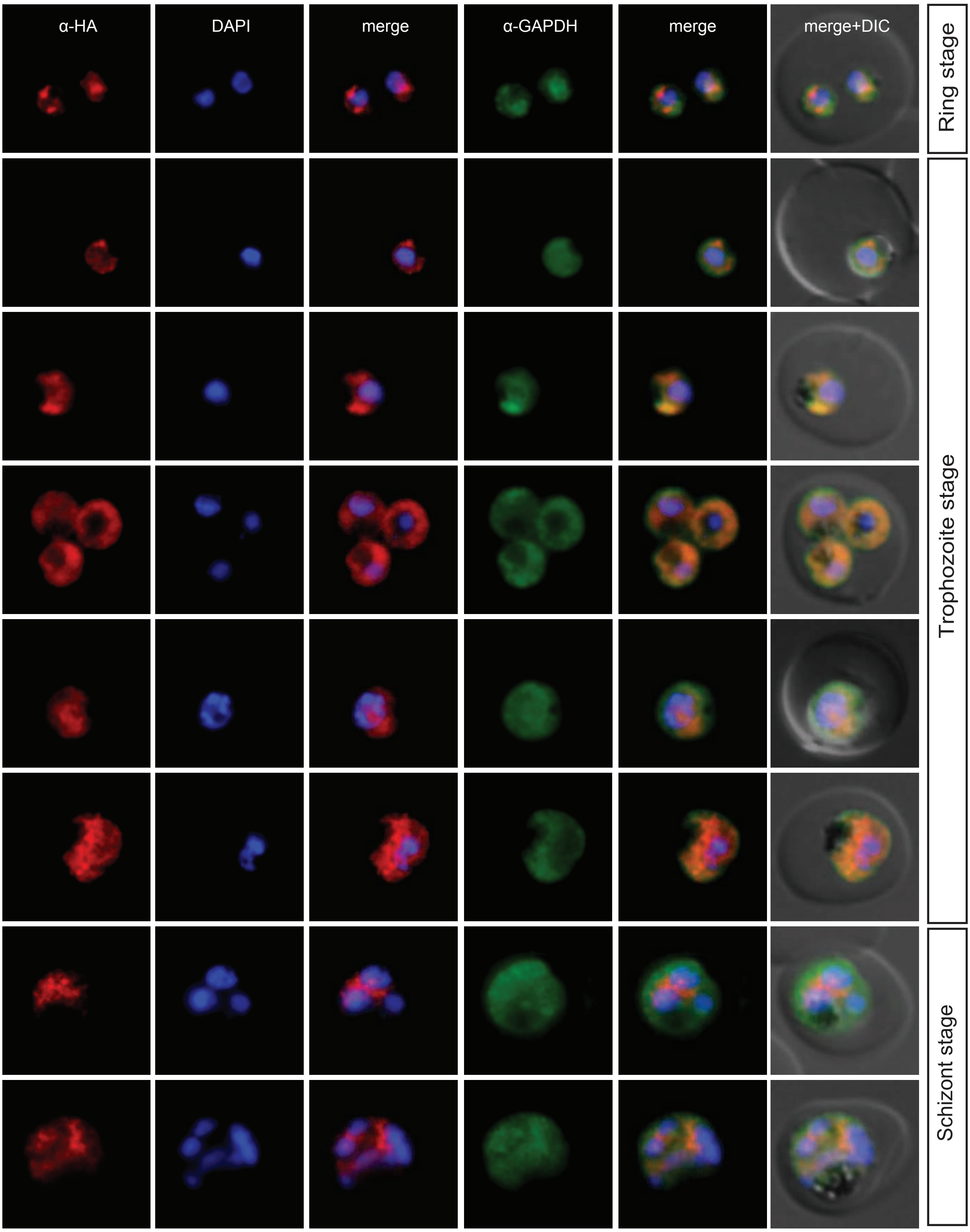

Localisation of NuProC4-3xHA (PF11_0293) during the IDC. Localisation of the tagged protein was visualised using anti-HA antibodies (red). Antibodies against GAPDH were used to visualise the cytosolic compartment. DAPI was used to visualise the nucleus. DIC images are shown as reference.

NuProC5 (PF13_0042)

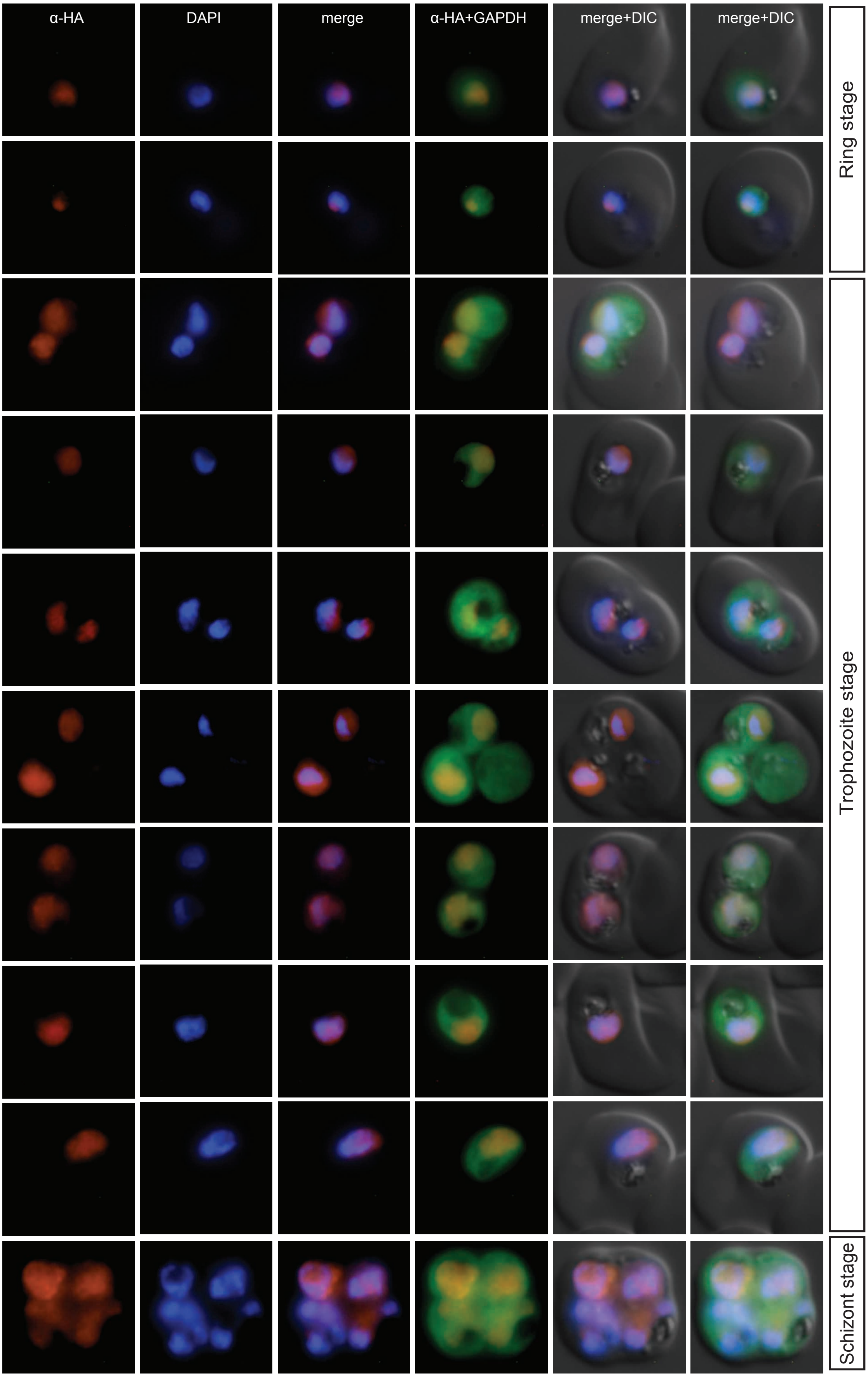

Localisation of NuProC5-3xHA (PF13_0042) during the IDC. Localisation of the tagged protein was visualised using anti-HA antibodies (red). Antibodies against GAPDH were used to visualise the cytosolic compartment. DAPI was used to visualise the nucleus. DIC images are shown as reference.

NuProC6 (PFL0635c)

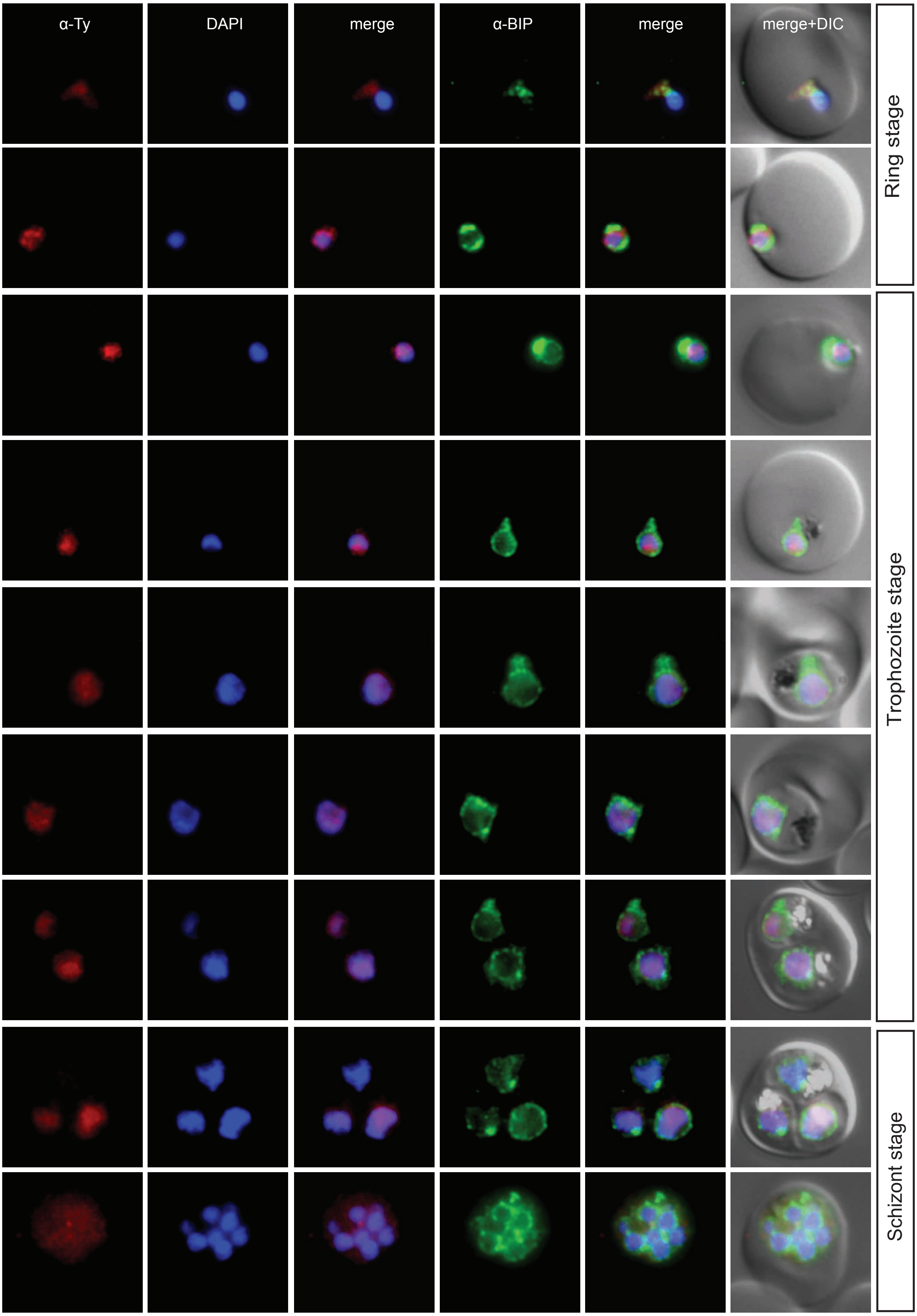

Localisation of NuProC6-2xTy (PFL0625c) during the IDC. Localisation of the tagged protein was visualised using anti-Ty antibodies (red). Antibodies against PfBIP were used to visualise the ER. DAPI was used to visualise the nucleus. DIC images are shown as reference.

NuProc7 (PF14_0393)

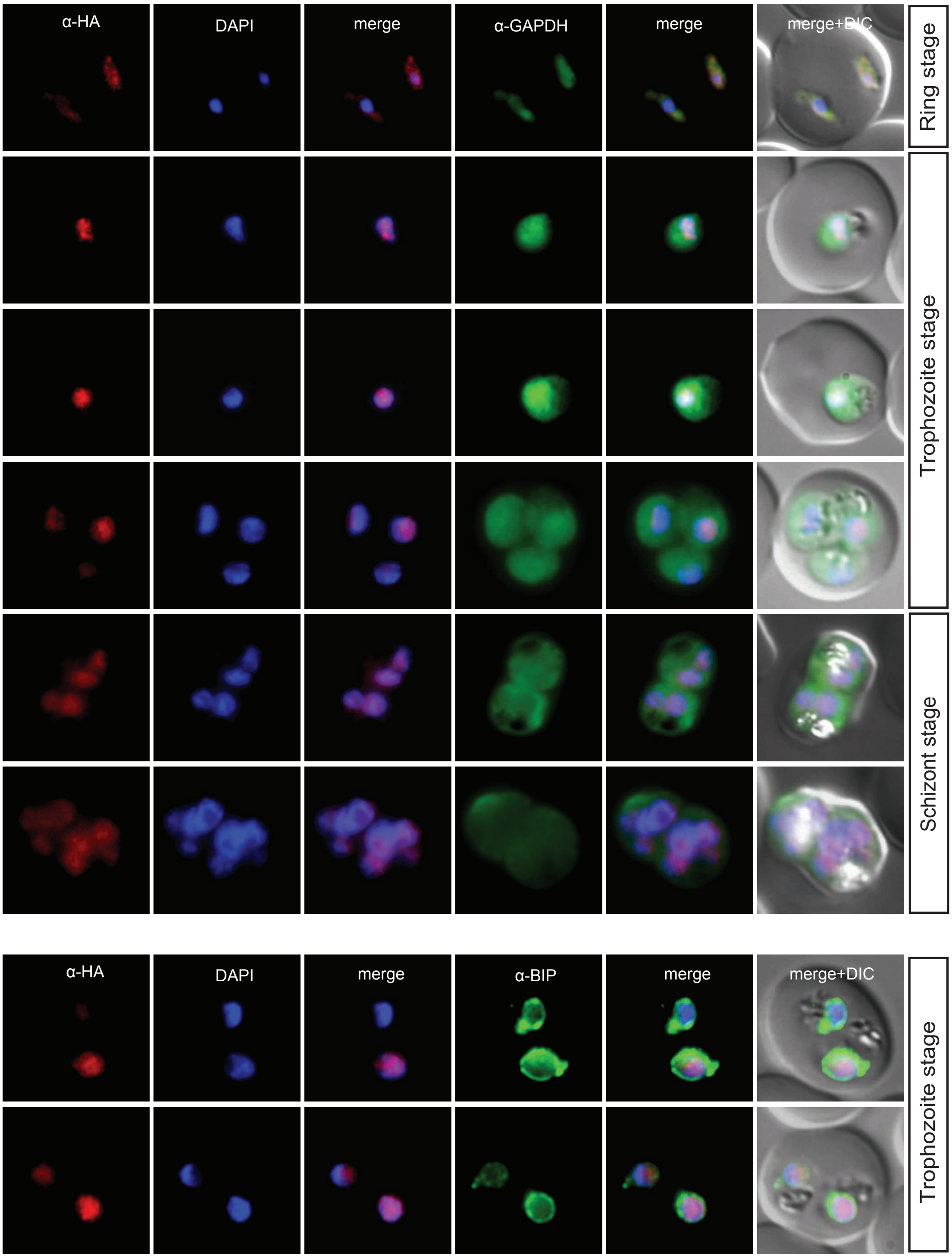

Localisation of NuProC7-3xHA (PF14_0393) during the IDC. Localisation of the tagged protein was visualised using anti-HA antibodies (red). Antibodies against GAPDH were used to visualise the cytosolic compartment. Antibodies against PfBIP were used to visualise the ER (bottom). DAPI was used to visualise the nucleus. DIC images are shown as reference.

NuProC8 (PF08_0083)

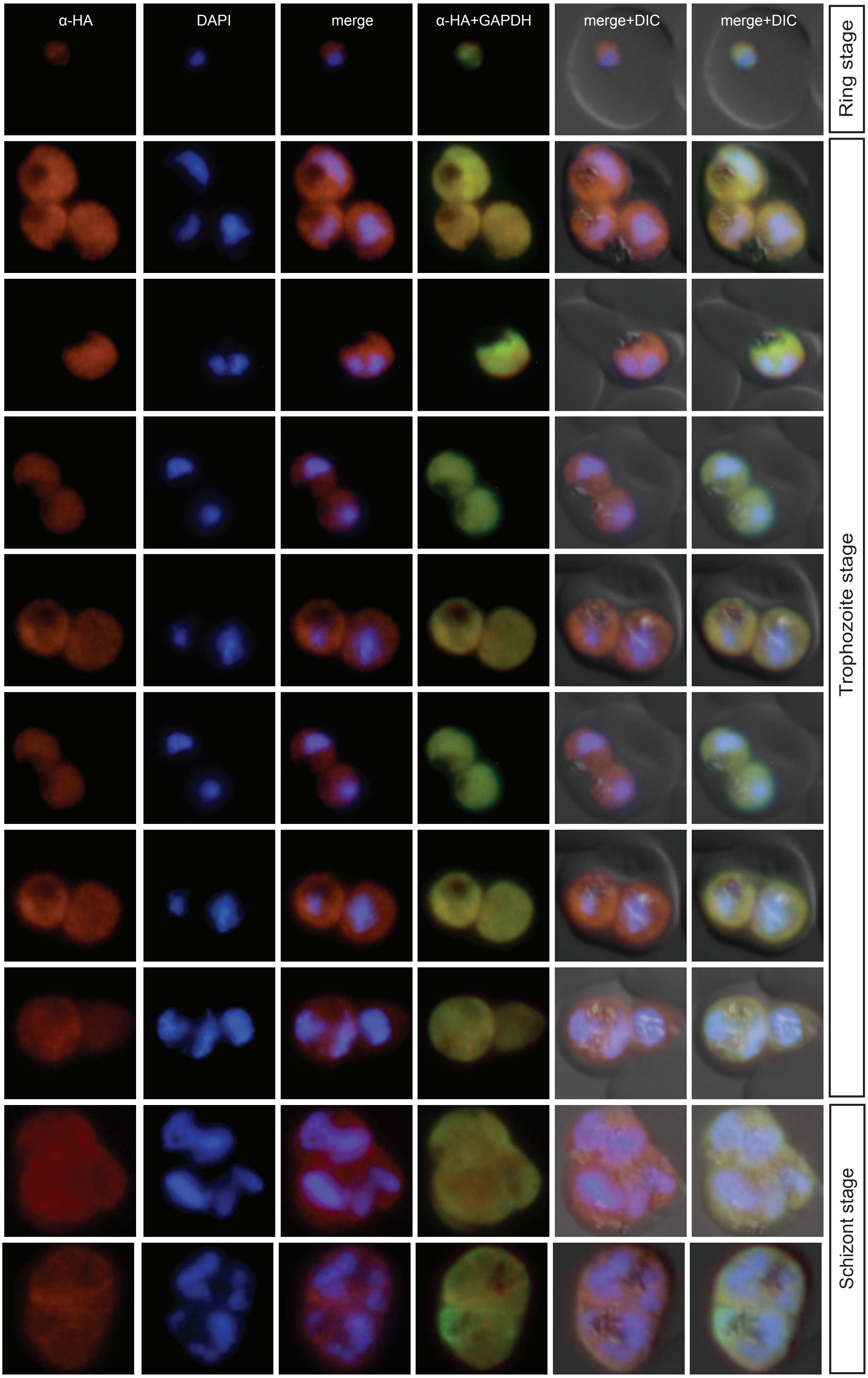

Localisation of NuProC8-3xHA (PF08_0083) during the IDC. Localisation of the tagged protein was visualised using anti-HA antibodies (red). Antibodies against GAPDH were used to visualise the cytosolic compartment. DAPI was used to visualise the nucleus. DIC images are shown as reference.

NuProC9 (PF10_0091)

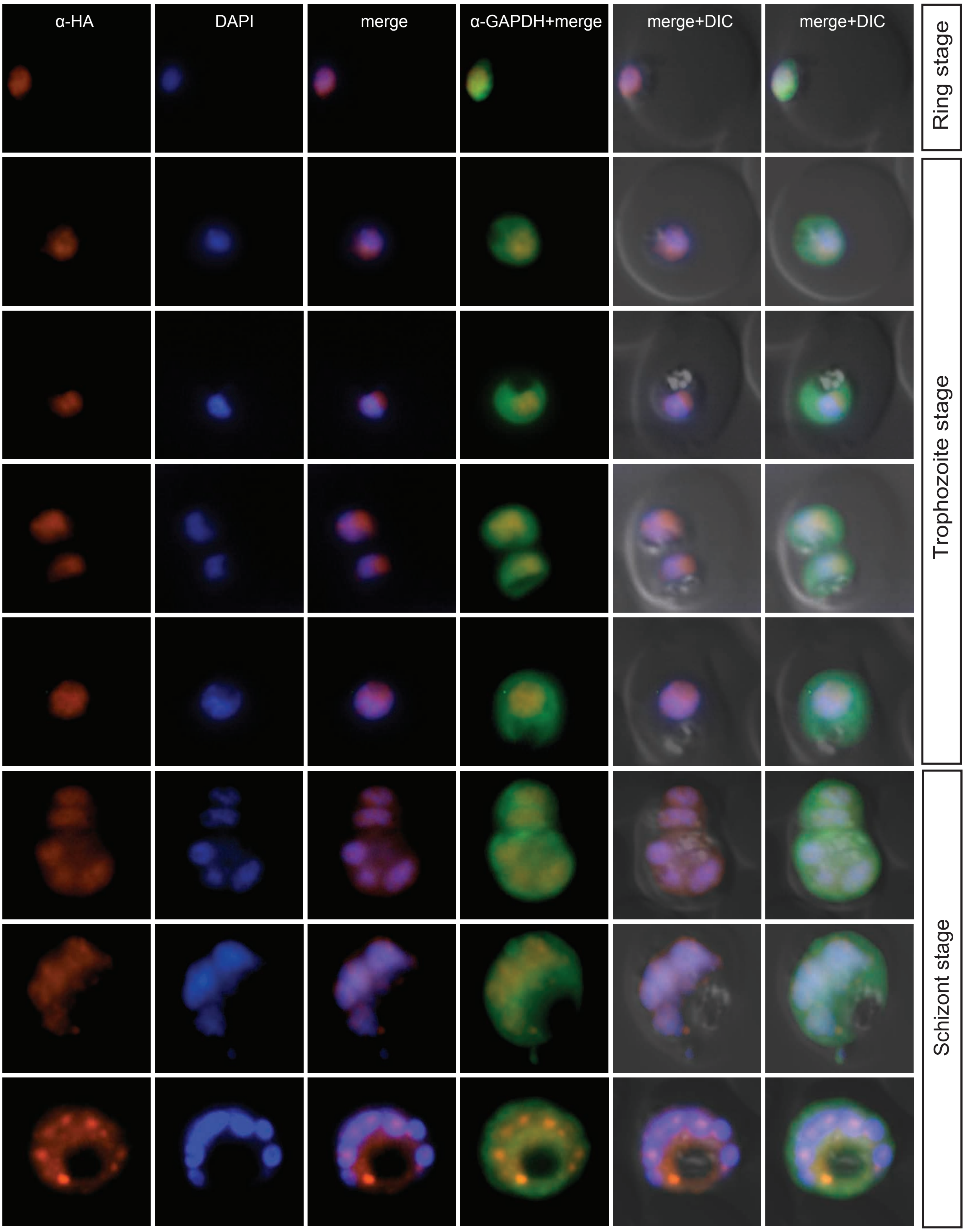

Localisation of NuProC9-3xHA (PF10_0091) during the IDC. Localisation of the tagged protein was visualised using anti-HA antibodies (red). Antibodies against GAPDH were used to visualise the cytosolic ompartment. DAPI was used to visualise the nucleus. DIC images are shown as reference.

NuProC10 (PF10_0328)

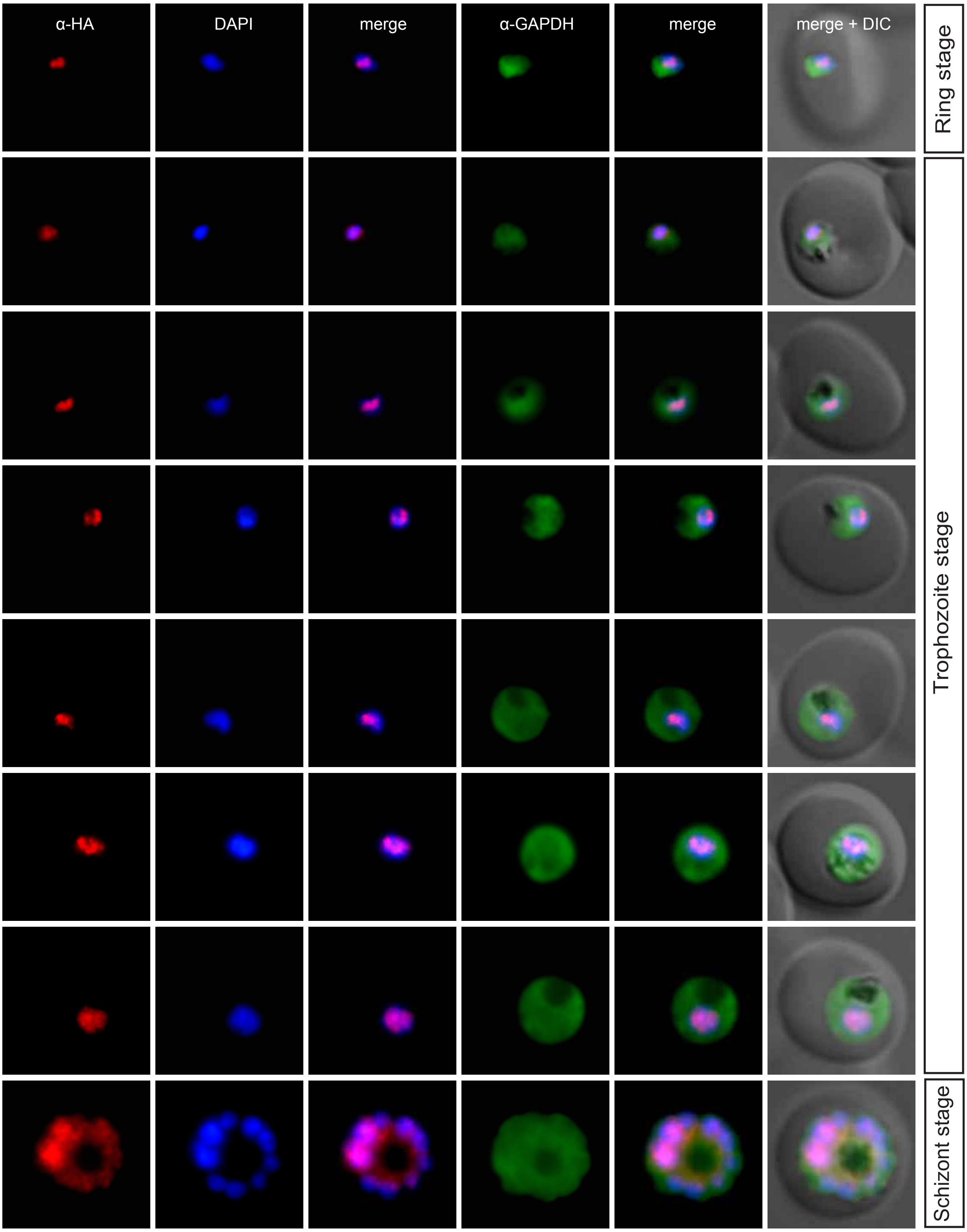

Localisation of NuProC10-3xHA (PF10_0328) during the IDC. Localisation of the tagged protein was visualised using anti-HA antibodies (red). Antibodies against GAPDH were used to visualise the cytosolic compartment. DAPI was used to visualise the nucleus. DIC images are shown as reference.

NuProC11 (PF11_0254)

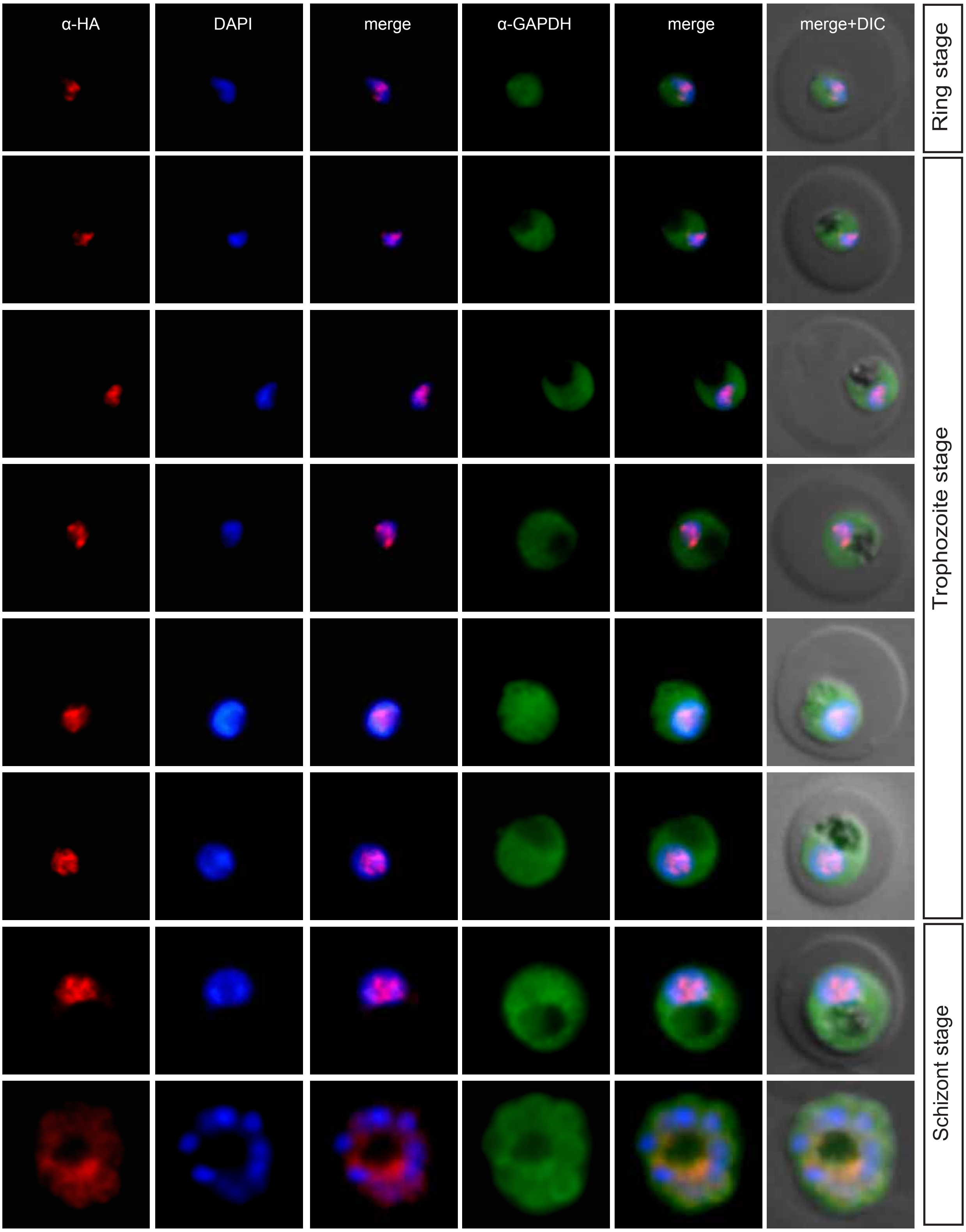

Localisation of NuProC11-3xHA (PF11_0254) during the IDC. Localisation of the tagged protein was visualised using anti-HA antibodies (red). Antibodies against GAPDH were used to visualise the cytosolic compartment. DAPI was used to visualise the nucleus. DIC images are shown as reference.

NuProC 12 (PF11_0332)

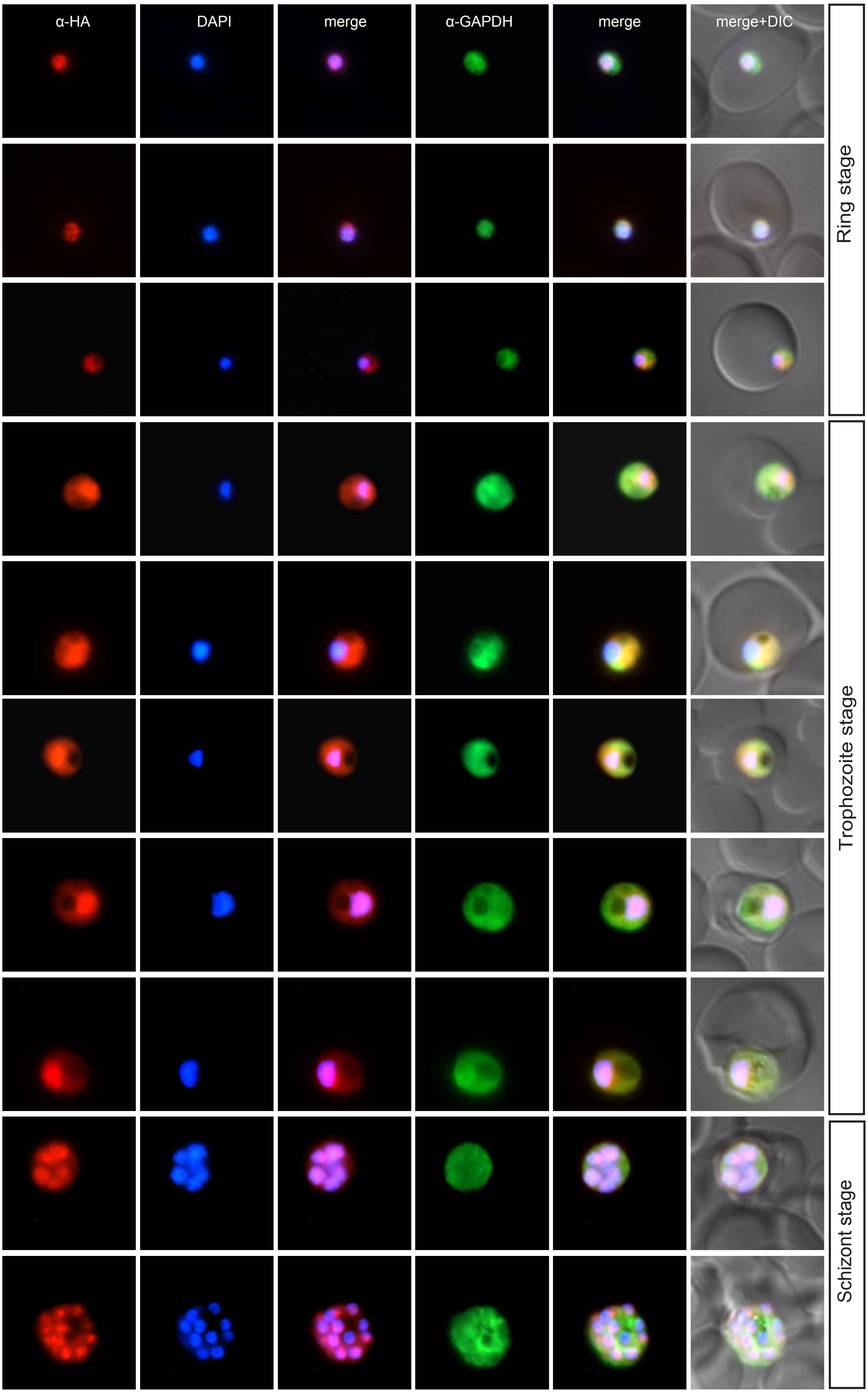

Localisation of NuProC12-3xHA (PF11_0332) during the IDC. Localisation of the tagged protein was visualised using anti-HA antibodies (red). Antibodies against GAPDH were used to visualise the cytosolic compartment. DAPI was used to visualise the nucleus. DIC images are shown as reference.

NuProC13 (PF13_0099)

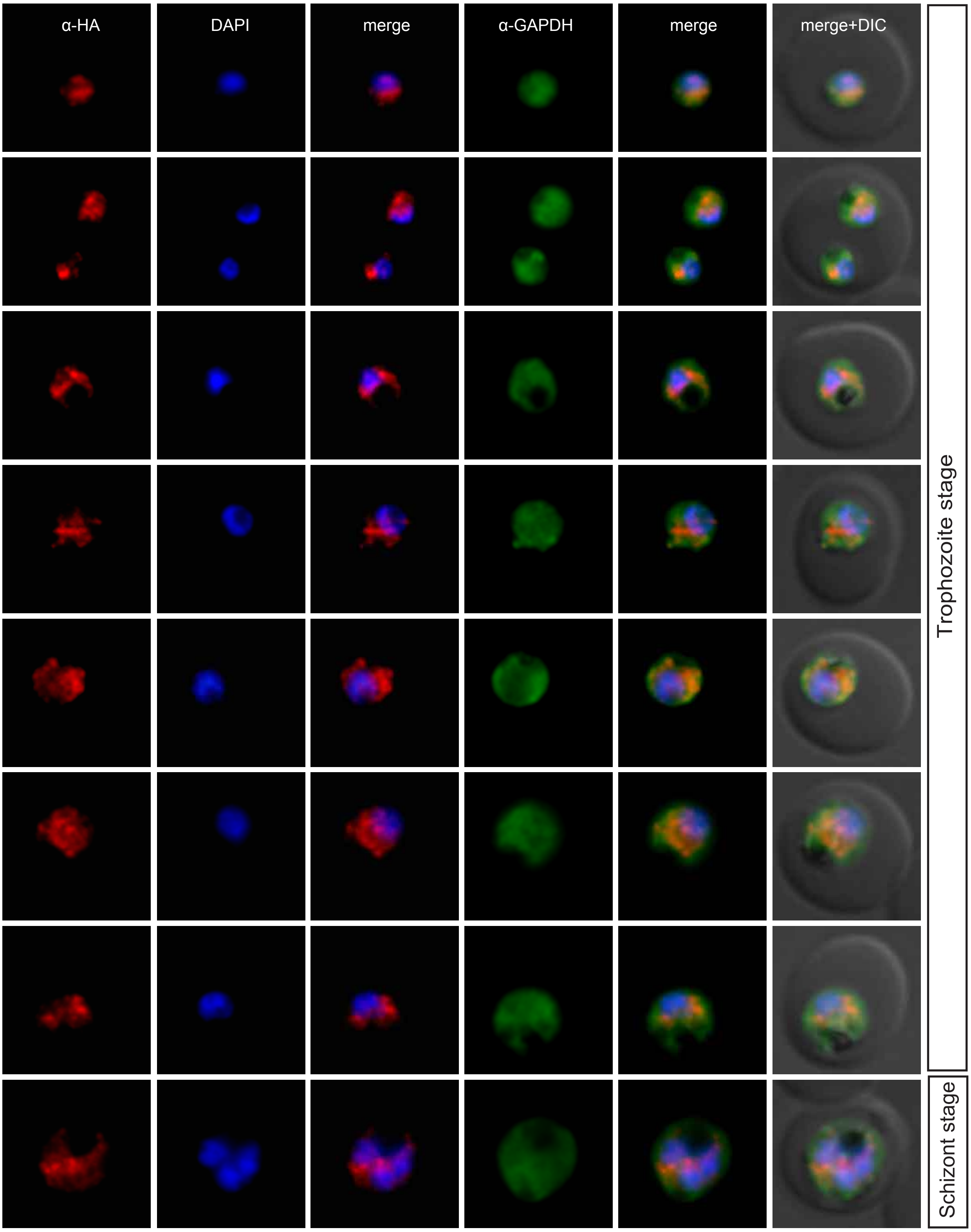

Localisation of NuProC13-3xHA (PF13_0099) during the IDC. Localisation of the tagged protein was visualised using anti-HA antibodies (red). Antibodies against GAPDH were used to visualise the cytosolic compartment. DAPI was used to visualise the nucleus. DIC images are shown as reference.

NuProC 14 (PF14_0176)

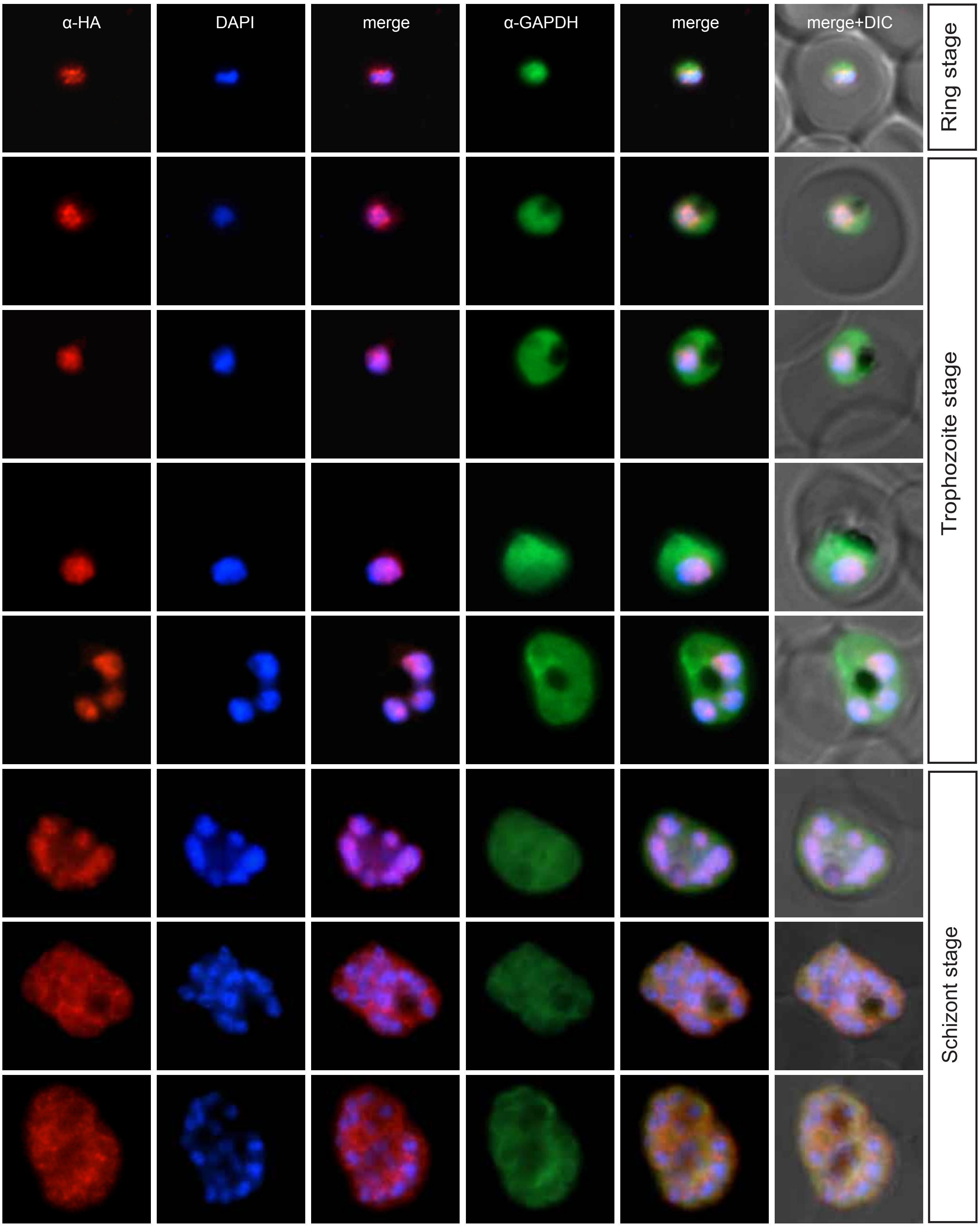

Localisation of NuProC14-3xHA (PF14_0176) during the IDC. Localisation of the tagged protein was visualised using anti-HA antibodies (red). Antibodies against GAPDH were used to visualise the cytosolic compartment. DAPI was used to visualise the nucleus. DIC images are shown as reference.

NuProC15 (PF14_0433)

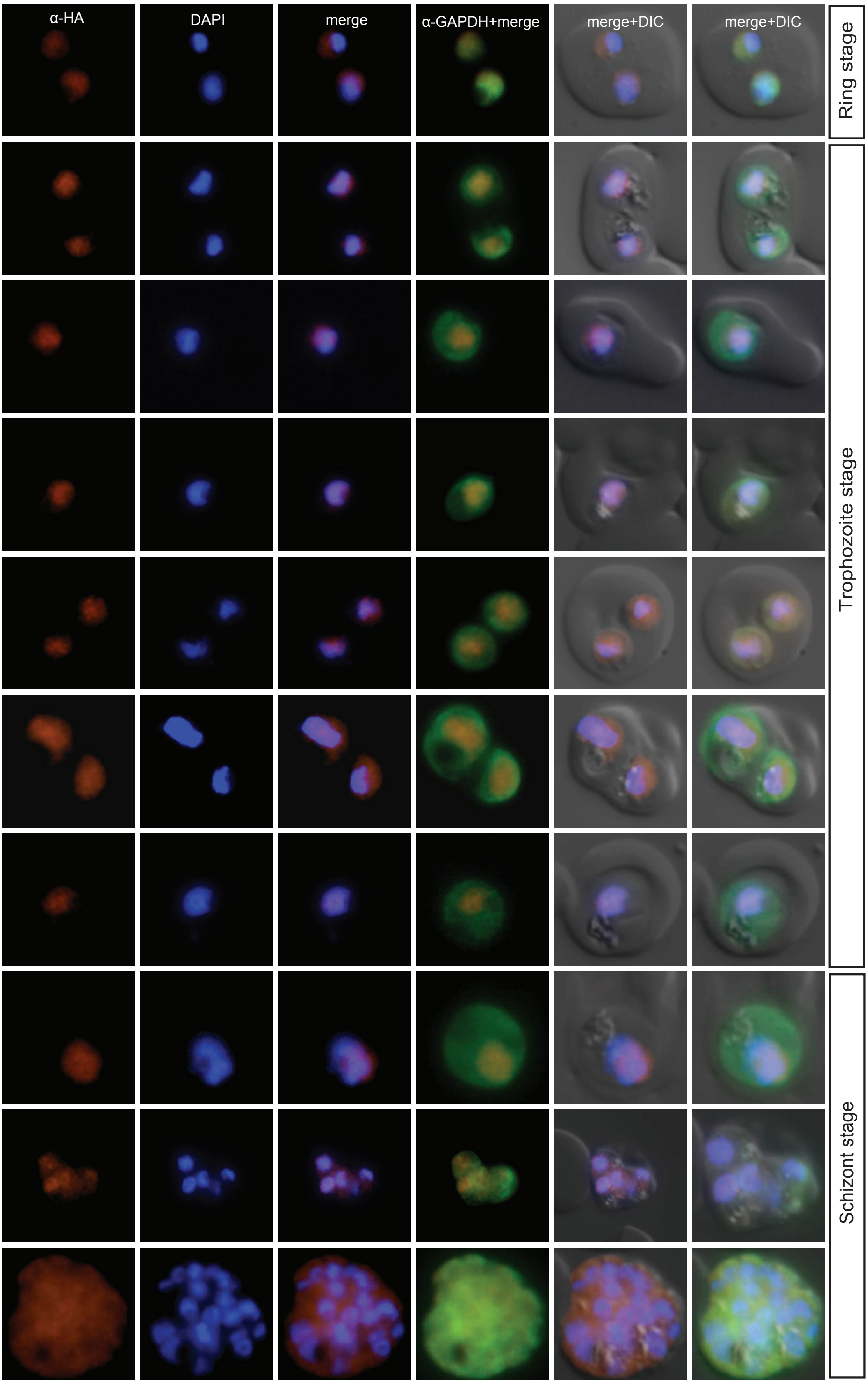

Localisation of NuProC15-3xHA (PF14_0433) during the IDC. Localisation of the tagged protein was visualised using anti-HA antibodies (red). Antibodies against GAPDH were used to visualise the cytosolic compartment. DAPI was used to visualise the nucleus. DIC images are shown as reference.

NuProC16 (PFC0126c)

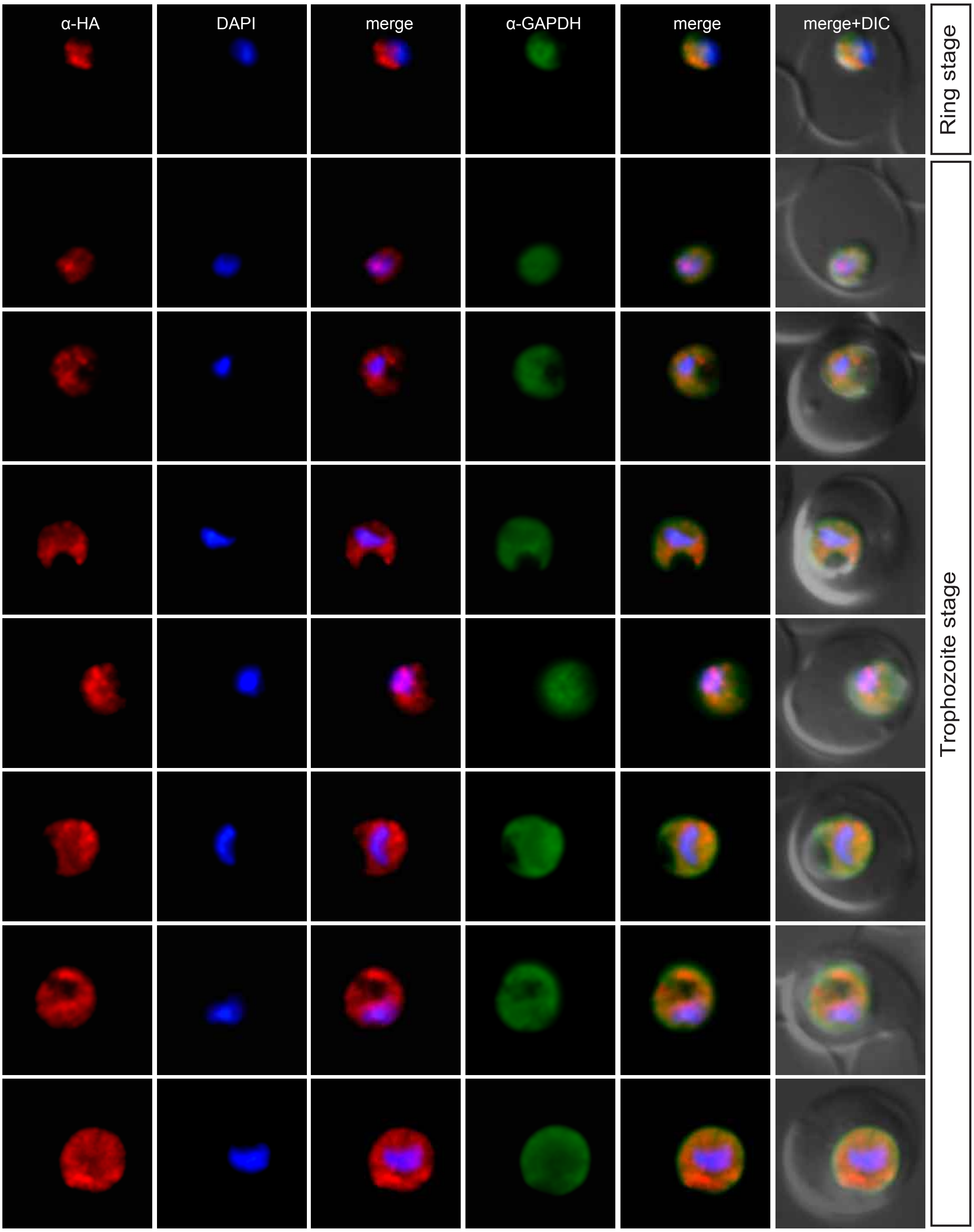

Localisation of NuProC16-3xHA (PFC0126c) during the IDC. Localisation of the tagged protein was visualised using anti-HA antibodies (red). Antibodies against GAPDH were used to visualise the cytosolic compartment. DAPI was used to visualise the nucleus. DIC images are shown as reference.

NuProC17 (PFC0130c)

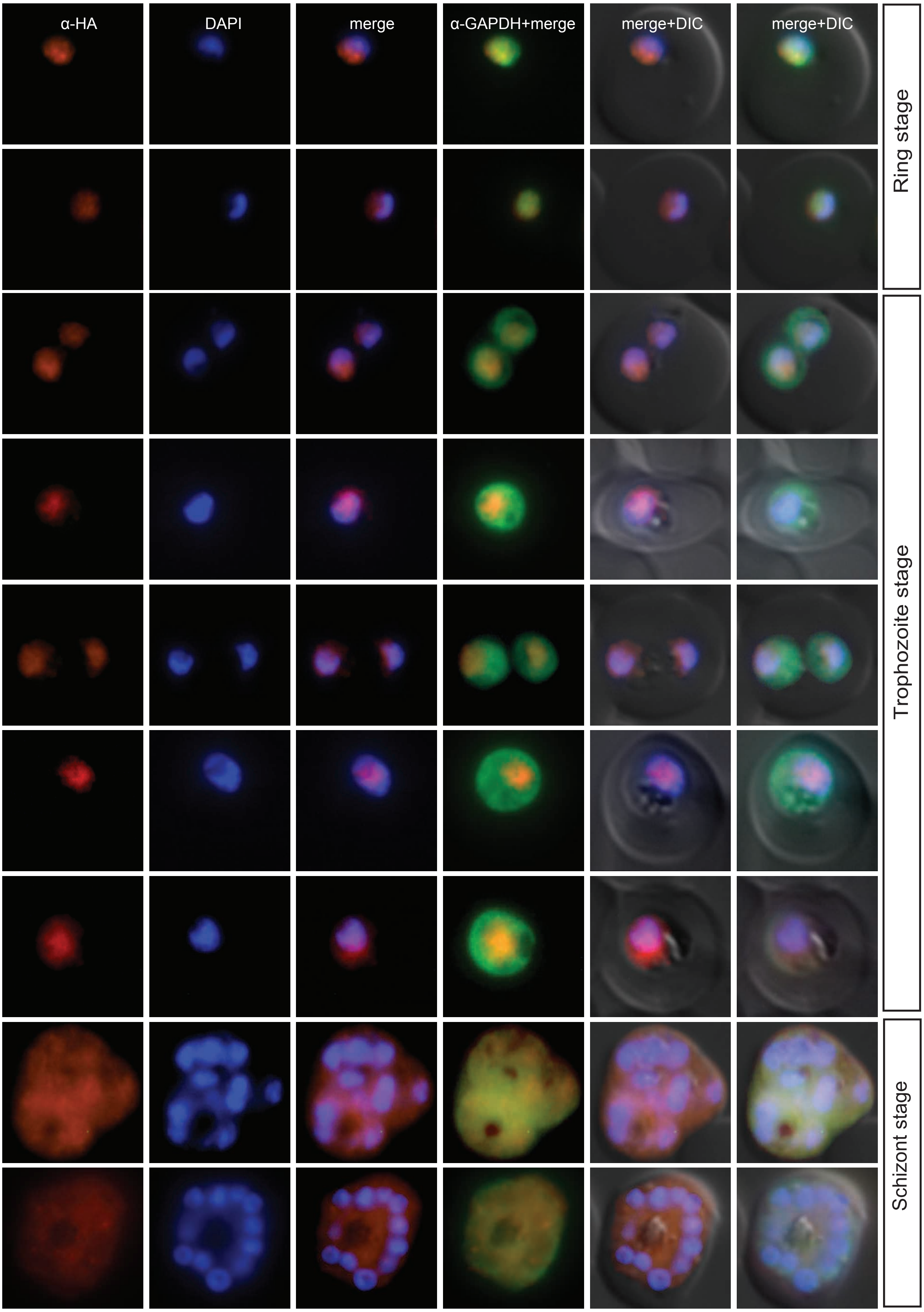

Localisation of NuProC17-3xHA (PFC0130c) during the IDC. Localisation of the tagged protein was visualised using anti-HA antibodies (red). Antibodies against GAPDH were used to visualise the cytosolic compartment. DAPI was used to visualise the nucleus. DIC images are shown as reference.

NuProC18 (PFC0690c)

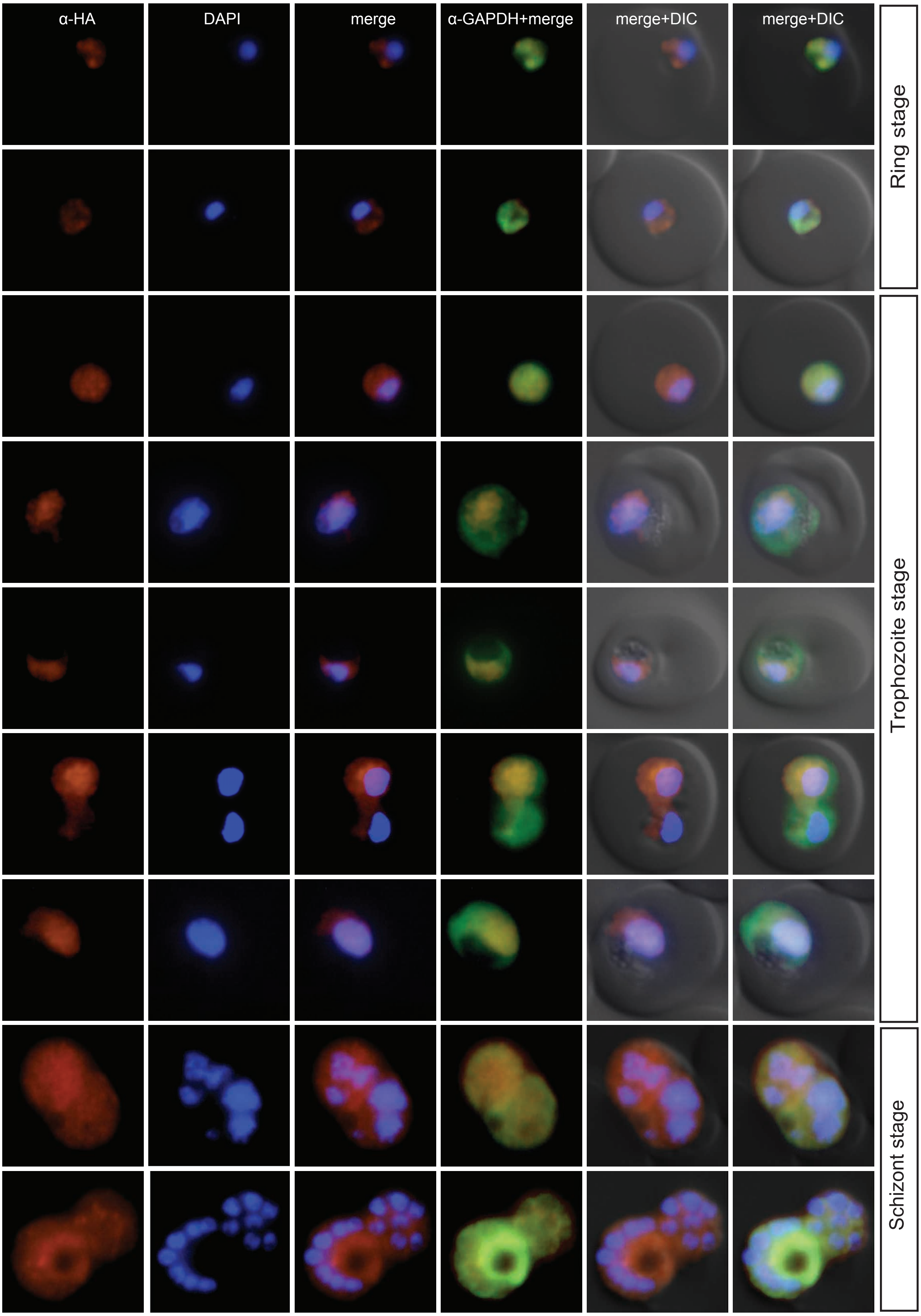

Localisation of NuProC18-3xHA (PFC0690c) during the IDC. Localisation of the tagged protein was visualised using anti-HA antibodies (red). Antibodies against GAPDH were used to visualise the cytosolic compartment. DAPI was used to visualise the nucleus. DIC images are shown as reference.

NuProC19 (PFI0610w)

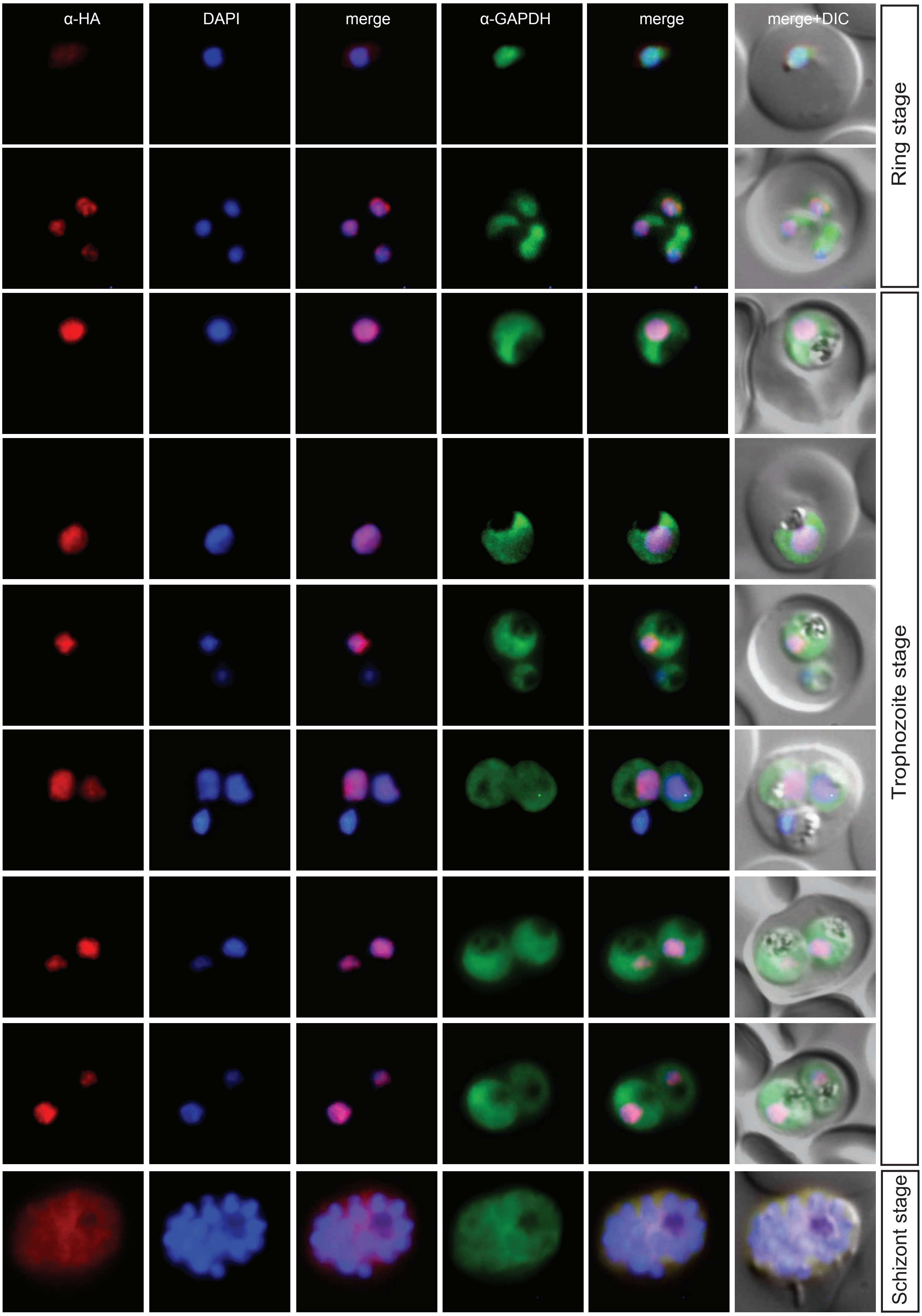

Localisation of NuProC19-3xHA (PFI0610w) during the IDC. Localisation of the tagged protein was visualised using anti-HA antibodies (red). Antibodies against GAPDH were used to visualise the cytosolic compartment. DAPI was used to visualise the nucleus. DIC images are shown as reference.

NuProC20 (PFI1355w)

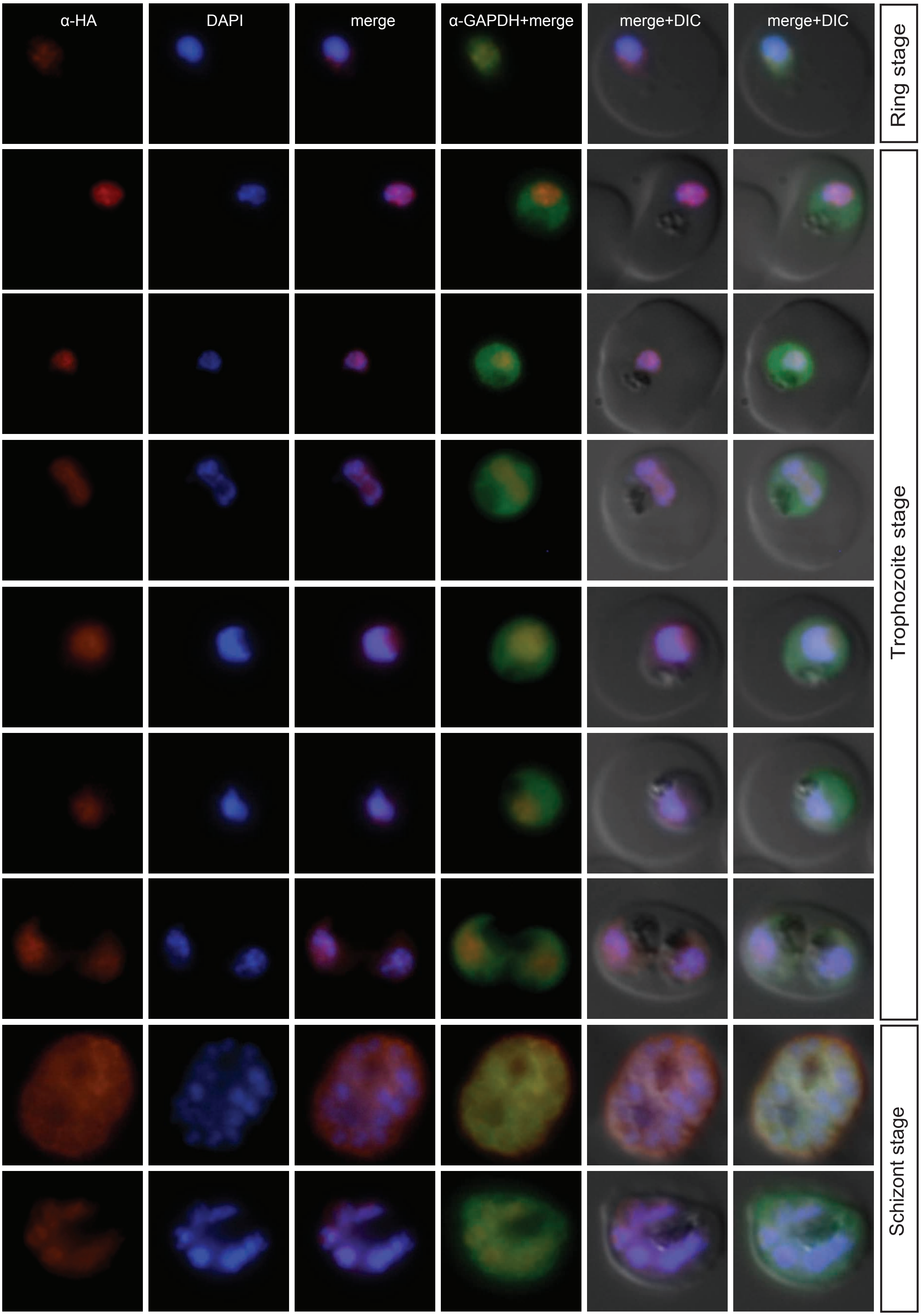

Localisation of NuProC20-3xHA (PFI1355w) during the IDC. Localisation of the tagged protein was visualised using anti-HA antibodies (red). Antibodies against GAPDH were used to visualise the cytosolic compartment. DAPI was used to visualise the nucleus. DIC images are shown as reference.

NuProC21 (PFL0185c)

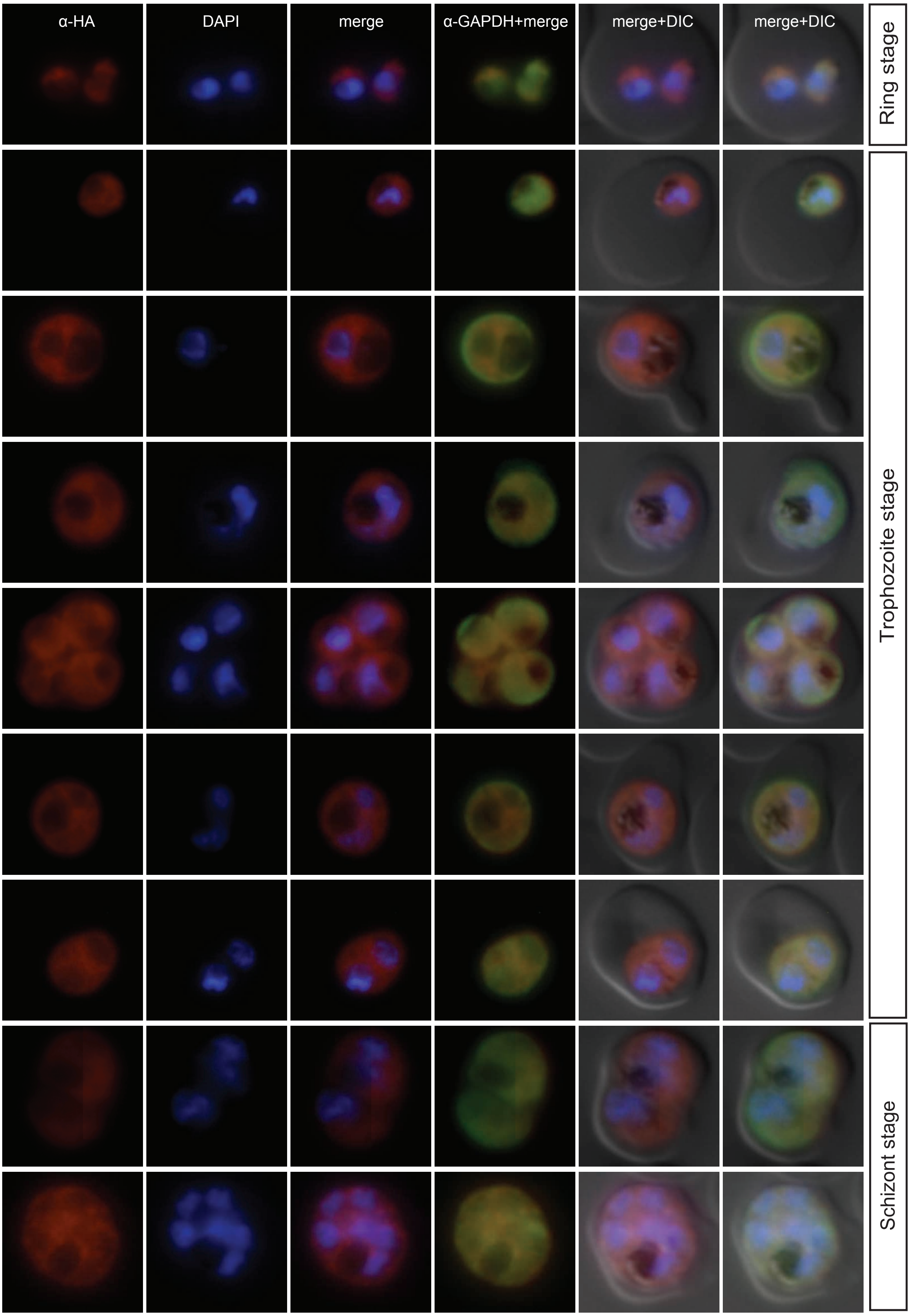

Localisation of NuProC21-3xHA (PFL0185c) during the IDC. Localisation of the tagged protein was visualised using anti-HA antibodies (red). Antibodies against GAPDH were used to visualise the cytosolic compartment. DAPI was used to visualise the nucleus. DIC images are shown as reference.

NuProC 22 (PFL0450c)

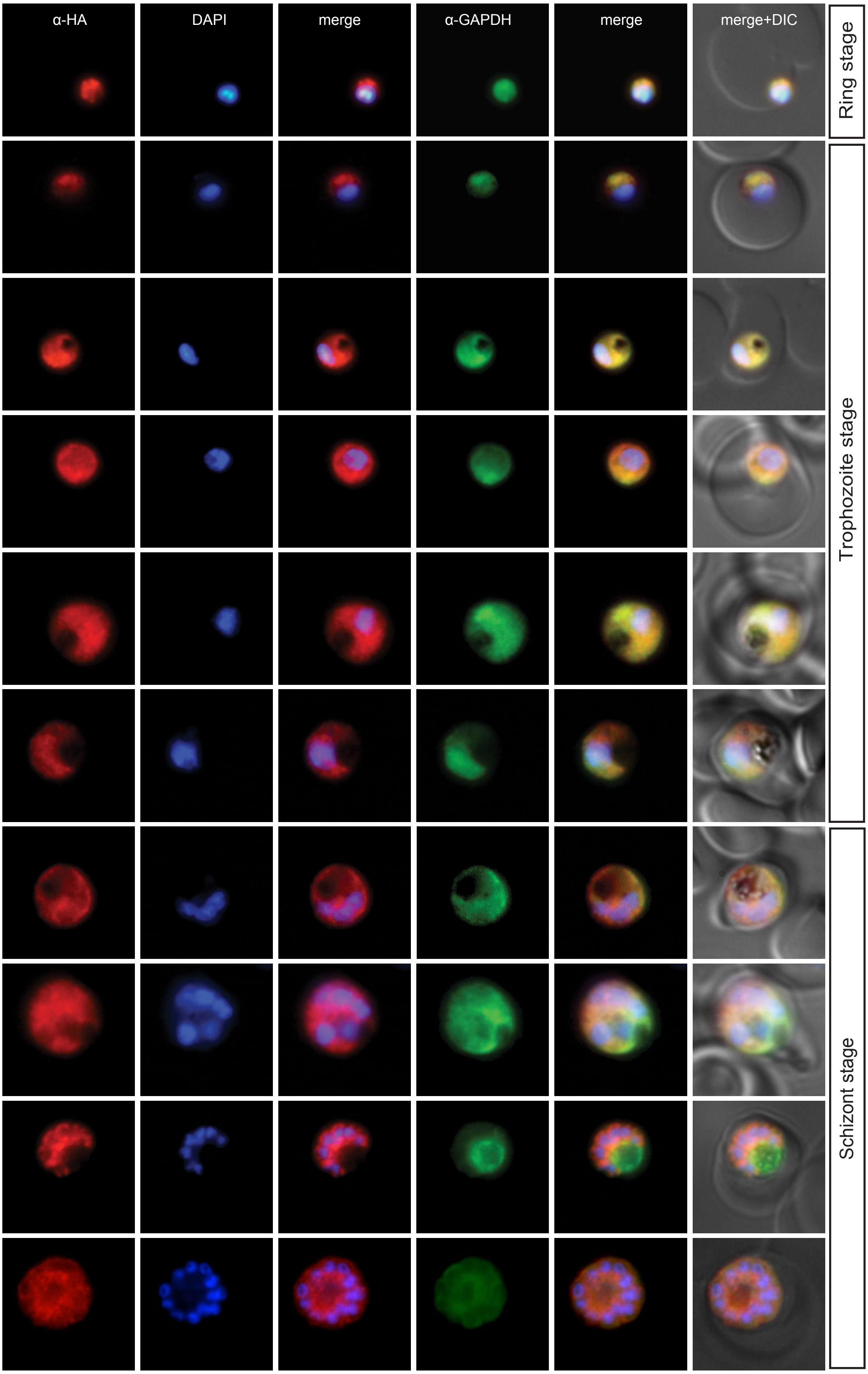

Localisation of NuProC22-3xHA (PFL0450c) during the IDC. Localisation of the tagged protein was visualised using anti-HA antibodies (red). Antibodies against GAPDH were used to visualise the cytosolic compartment. DAPI was used to visualise the nucleus. DIC images are shown as reference.
