# Supplementary material for: Organellar proteomics reveals hundreds of novel nuclear proteins in the malaria parasite Plasmodium falciparum
Source: Genome Biol. 2012 Nov 26;13(11):R108. doi: 10.1186/gb-2012-13-11-r108 (PMC4053738; doi:10.1186/gb-2012-13-11-r108)
Supplement: Additional file 13 — Detailed IFA localization of n-NuProCs 1 to 6 during the IDC. [file gb-2012-13-11-r108-S13.PDF]

Additional file 13_Oehring et al.
Detailed IFA analyses of n-NuProCs 1-6 during the IDC.

n-NuProC1 (PF11_0099)

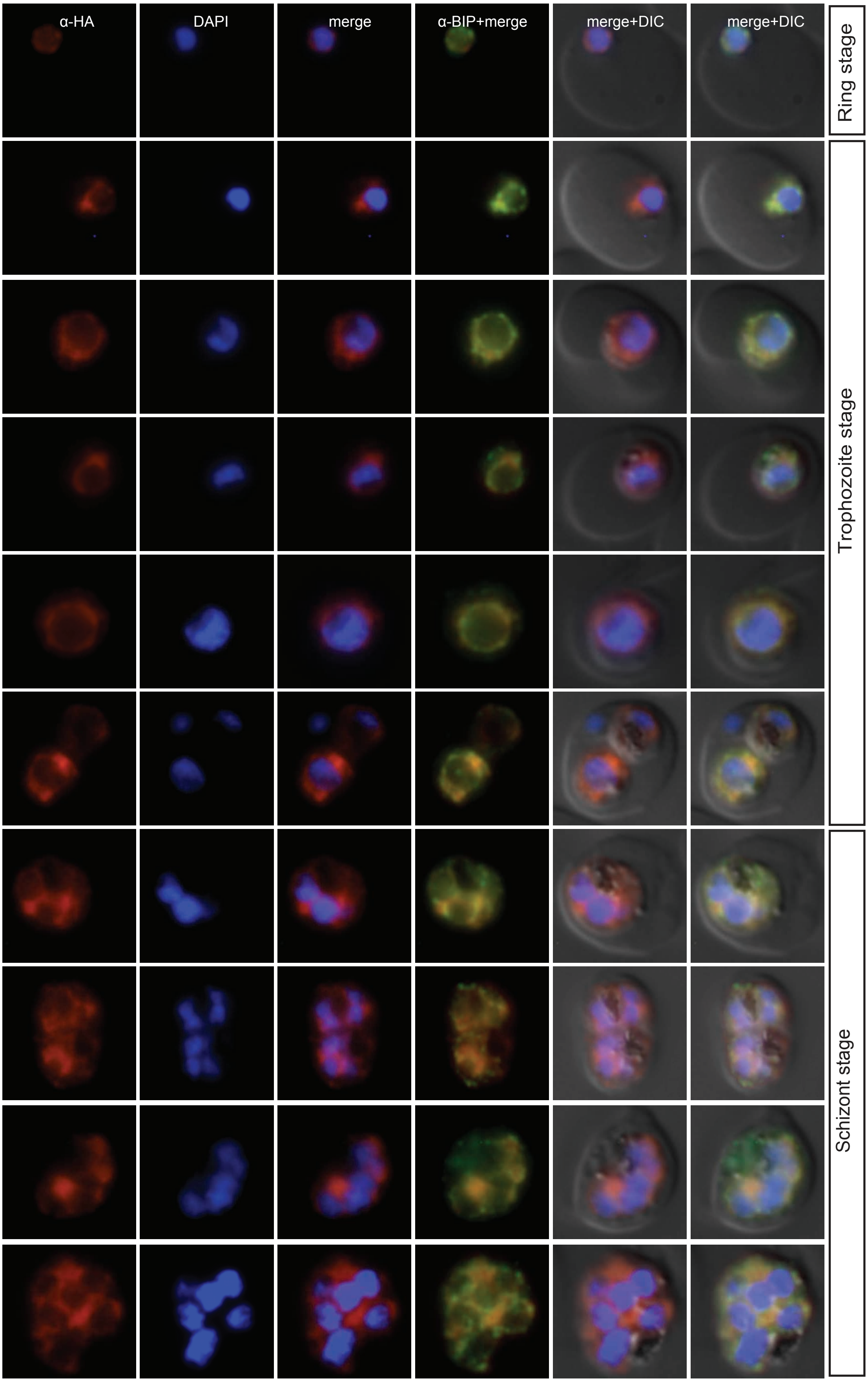

Localisation of n-NuProC1-3xHA (PF11_0099) during the IDC. Localisation of the tagged protein was visualised using anti-HA antibodies (red). Antibodies against PfBIP were used to visualise the ER. DAPI was used to visualise the nucleus. DIC images are shown as reference.

n-NuProC2 (MAL7P1.77)

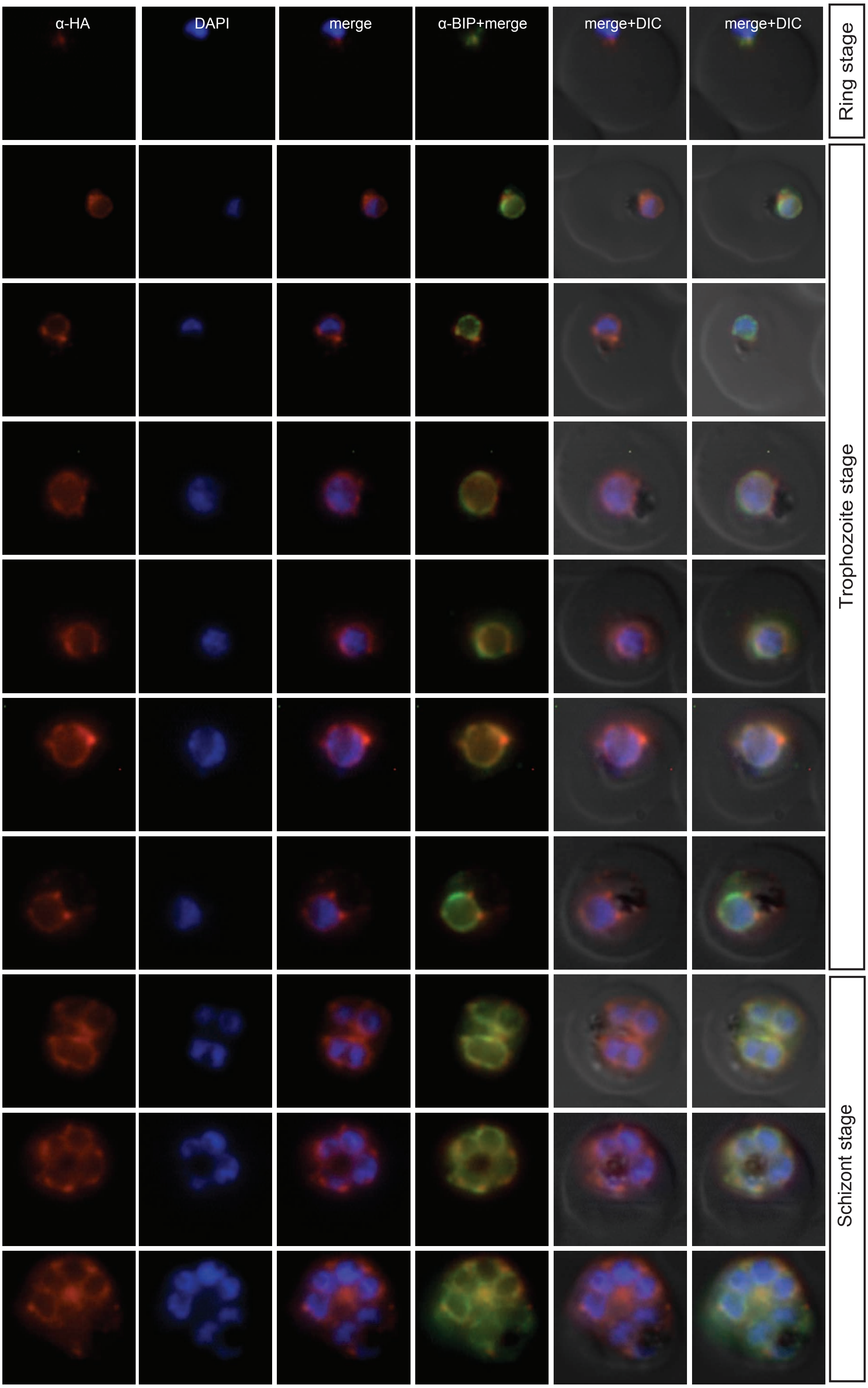

Localisation of n-NuProC2-3xHA (MAL7P1.77) during the IDC. Localisation of the tagged protein was visualised using anti-HA antibodies (red). Antibodies against PfBIP were used to visualise the ER. DAPI was used to visualise the nucleus. DIC images are shown as reference.

n-NuProC3 (PF07_0007)

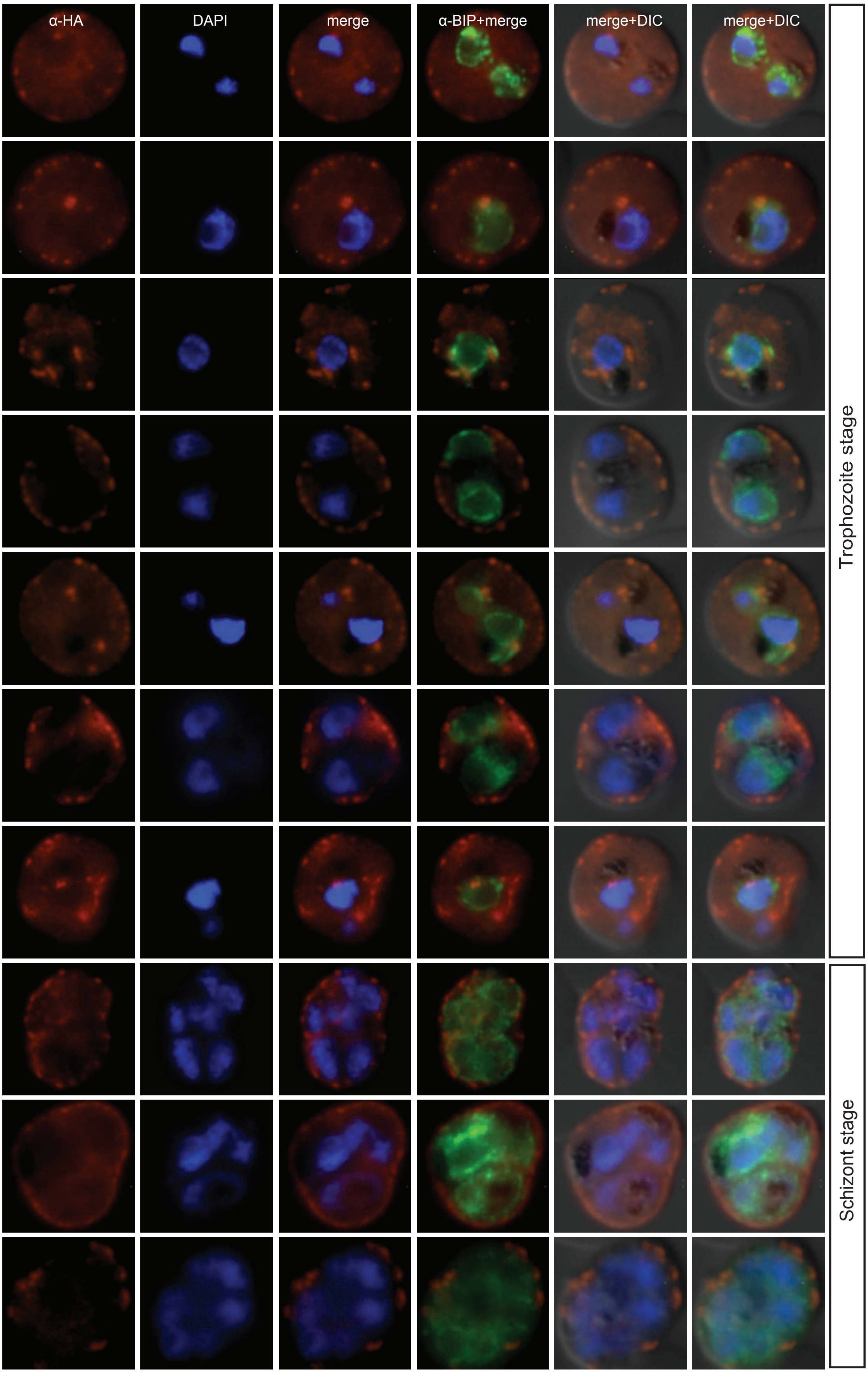

Localisation of n-NuProC3-3xHA (PF07_0007) during the IDC. Localisation of the tagged protein was visualised using anti-HA antibodies (red). Antibodies against PfBIP were used to visualise the ER. DAPI was used to visualise the nucleus. DIC images are shown as reference.

n-NuProC4 (PF10_0100)

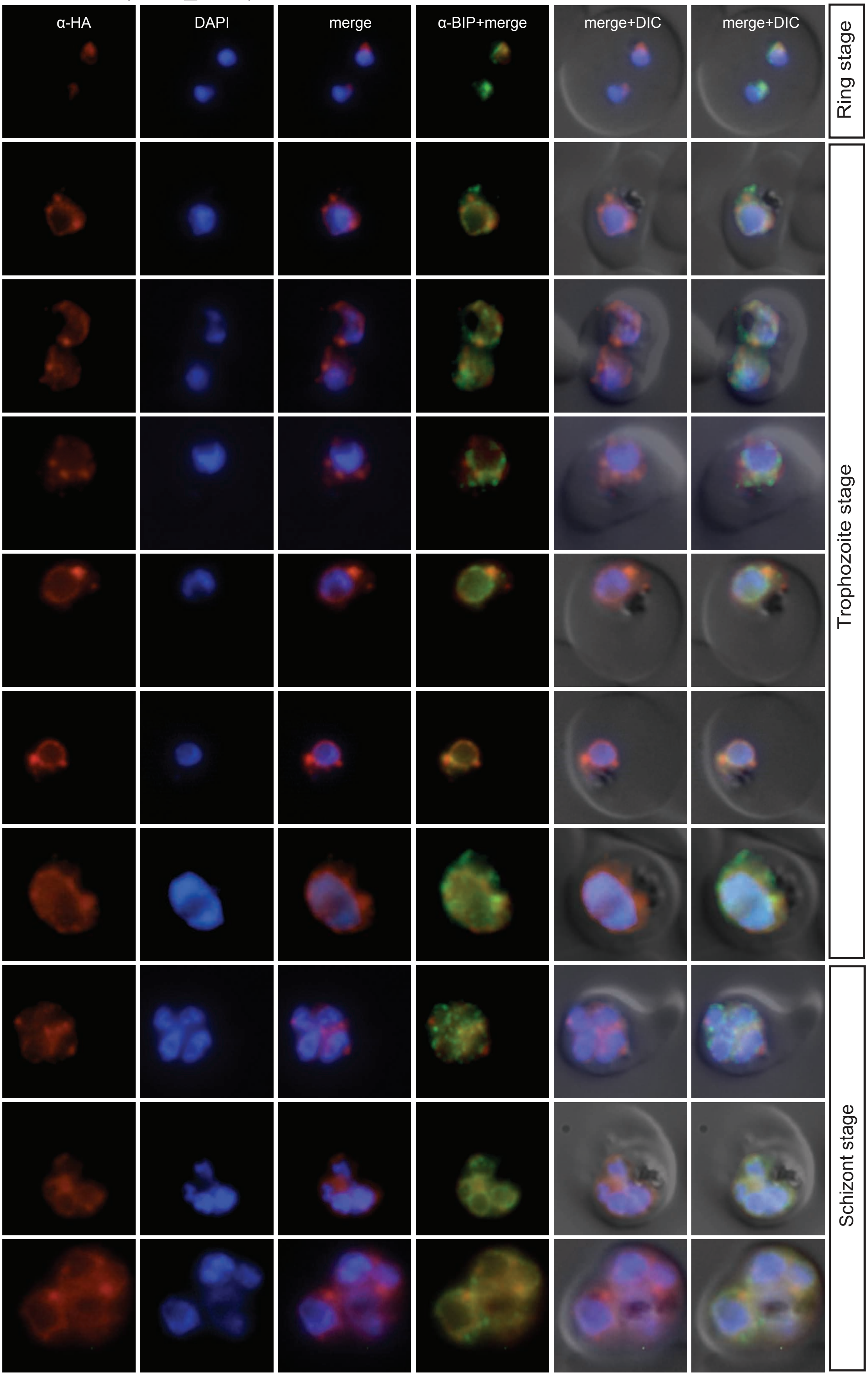

Localisation of n-NuProC4-3xHA (PF10_0100) during the IDC. Localisation of the tagged protein was visualised using anti-HA antibodies (red). Antibodies against PfBIP were used to visualise the ER. DAPI was used to visualise the nucleus. DIC images are shown as reference.

n-NuProC5 (PF11_0179)

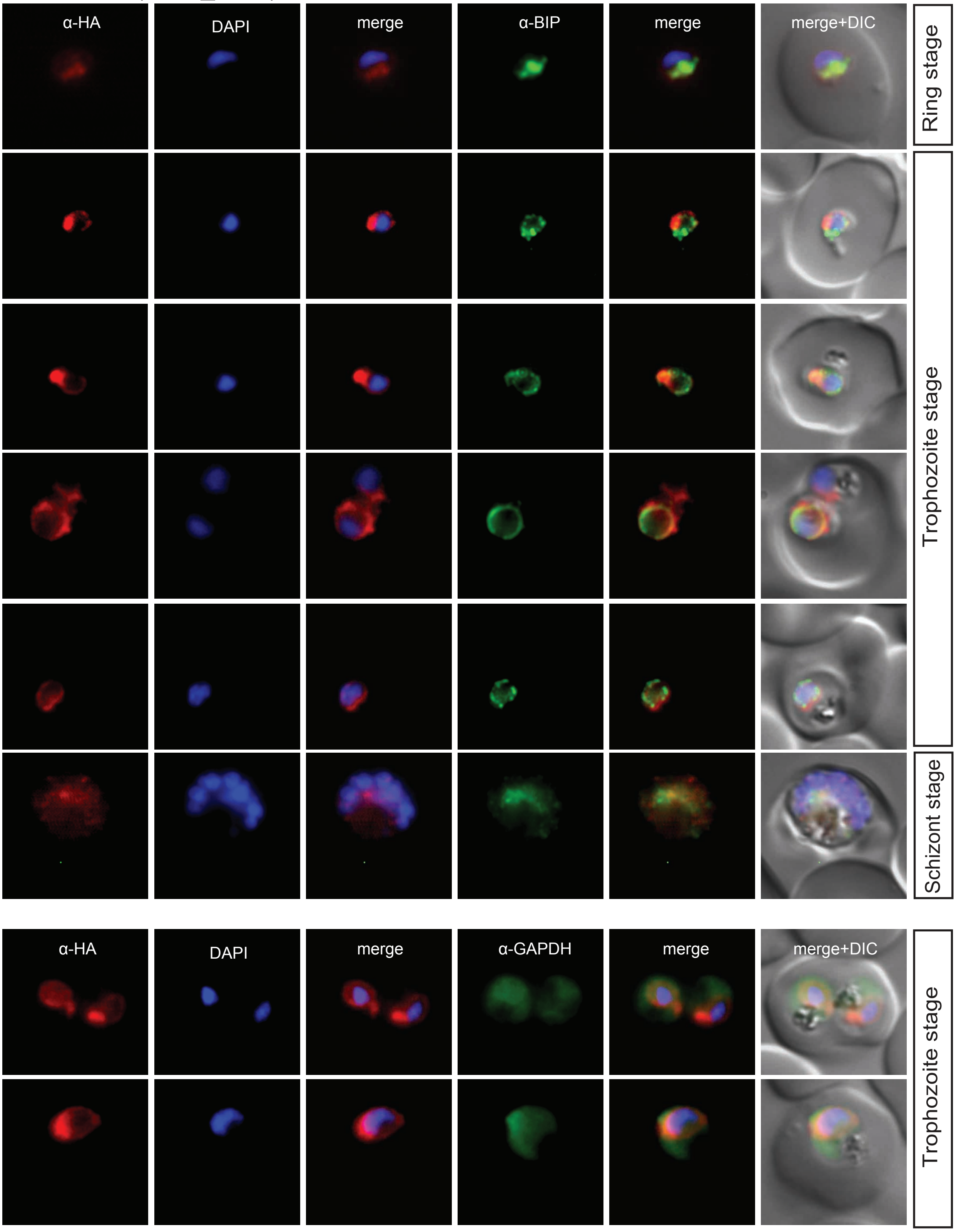

Localisation of n-NuProC5-3xHA (PF11_0179) during the IDC. Localisation of the tagged protein was visualised using anti-HA antibodies (red). Antibodies against PfBIP were used to visualise the ER. Antibodies against GAPDH were used to visualise the cytosolic compartment (bottom). DAPI was used to visualise the nucleus. DIC images are shown as reference.

n-NuProC6 (PFB0395w)

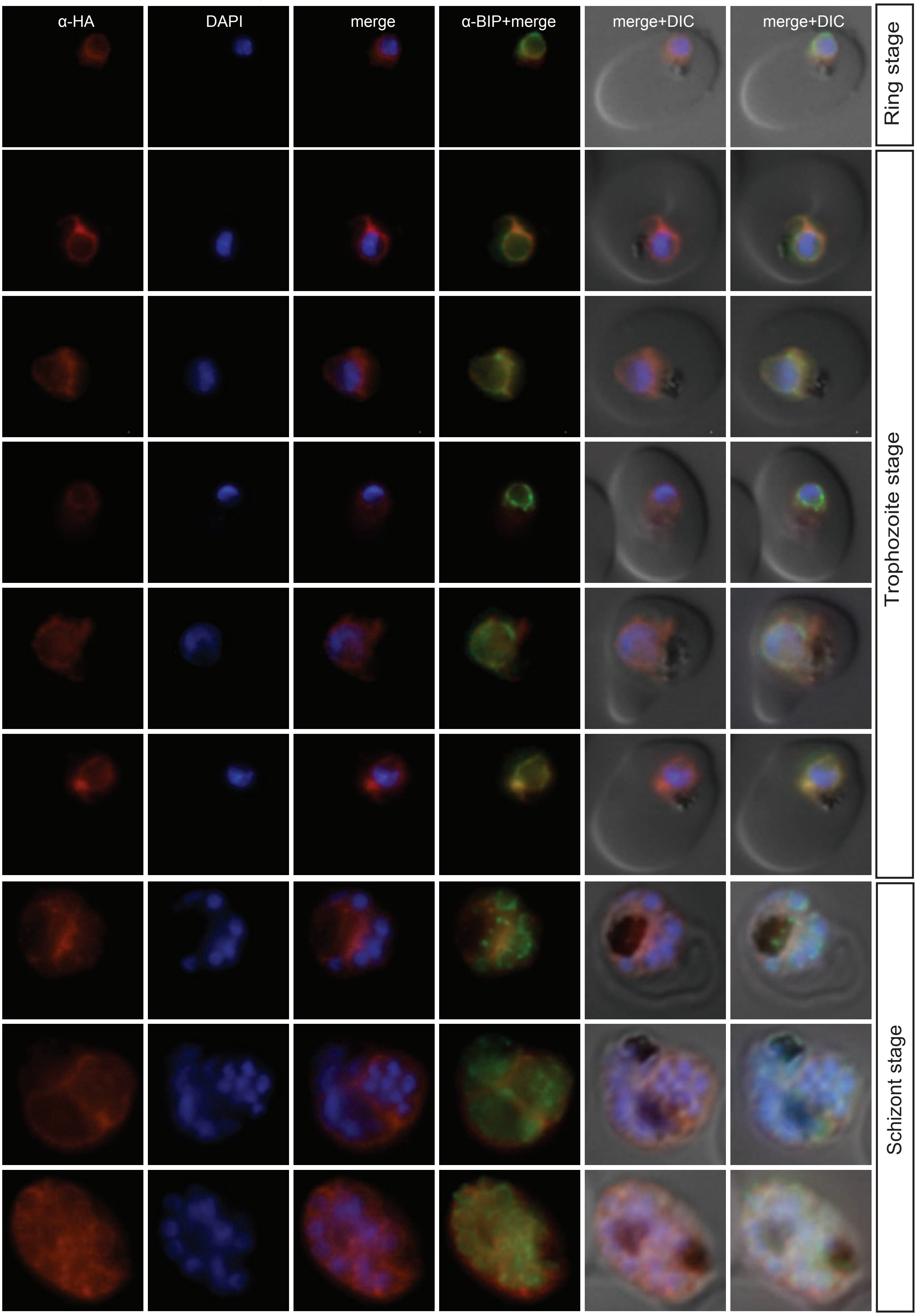

Localisation of n-NuProC6-3xHA (PFB0395w) during the IDC. Localisation of the tagged protein was visualised using anti-HA antibodies (red). Antibodies against PfBIP were used to visualise the ER. DAPI was used to visualise the nucleus. DIC images are shown as reference.
