# Supplementary material for: Organellar proteomics reveals hundreds of novel nuclear proteins in the malaria parasite Plasmodium falciparum
Source: Genome Biol. 2012 Nov 26;13(11):R108. doi: 10.1186/gb-2012-13-11-r108 (PMC4053738; doi:10.1186/gb-2012-13-11-r108)
Supplement: Additional file 14 — Multiple sequence alignments in graphical format of: (1) the ACDC domains in apicomplexan proteins; (2) the apicomplexan proteins encoding the partial CSTF domain; (3) the P. falciparum proteins encoding the extended ELM2 domain; (4) the P. falciparum proteins encoding the MYND domain. [file gb-2012-13-11-r108-S14.PDF]

Additional file 14_Oehring et al.

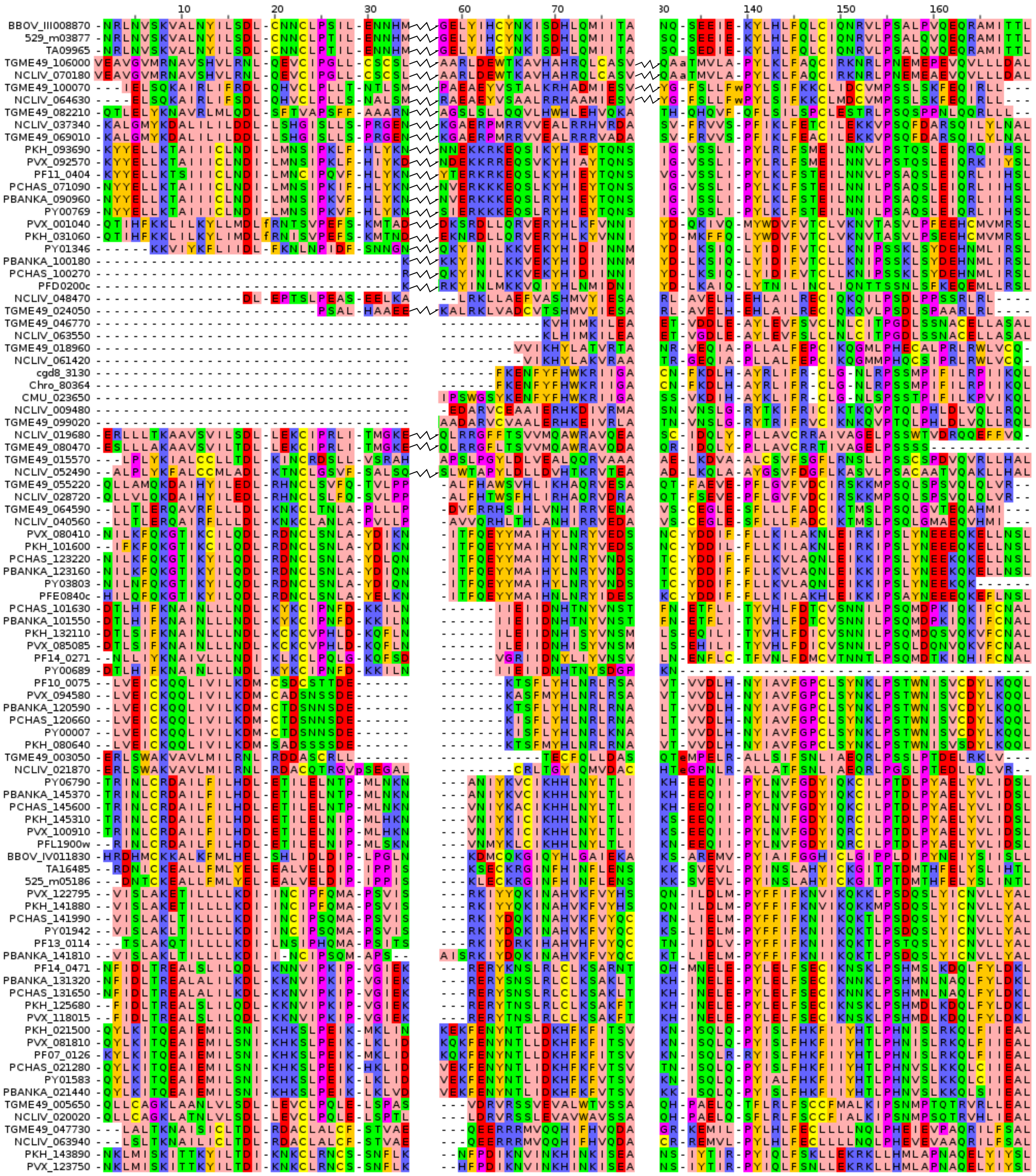

ACDC domain alignment graphic. A multiple sequence alignment of the ACDC domains in apicomplexan proteins. The raw data used to generate this graphic and the location of the domain in each protein's sequence can be found in Additional file 15. Sequence identifiers refer to EuPathDB gene identifiers. Jagged lines indicate unconserved insertions in the domain.

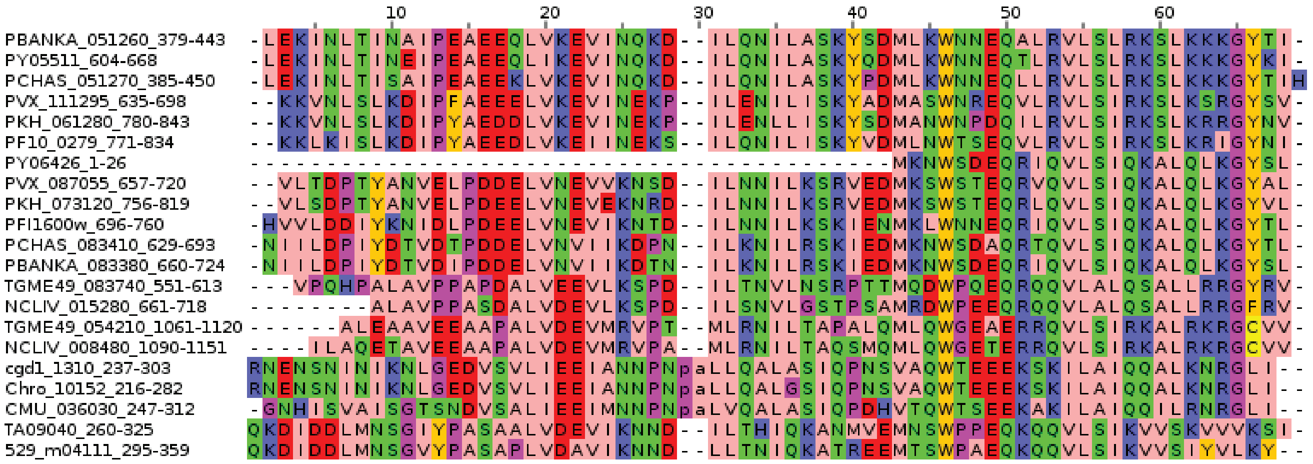

Partial CSTF domain alignment graphic. A multiple sequence alignment of the apicomplexan proteins encoding the domain is shown in graphical format. The raw data used to generate this graphic and the location of the domain in each protein's sequence can be found in Additional file 21. Sequence identifiers refer to EuPathDB gene identifiers.

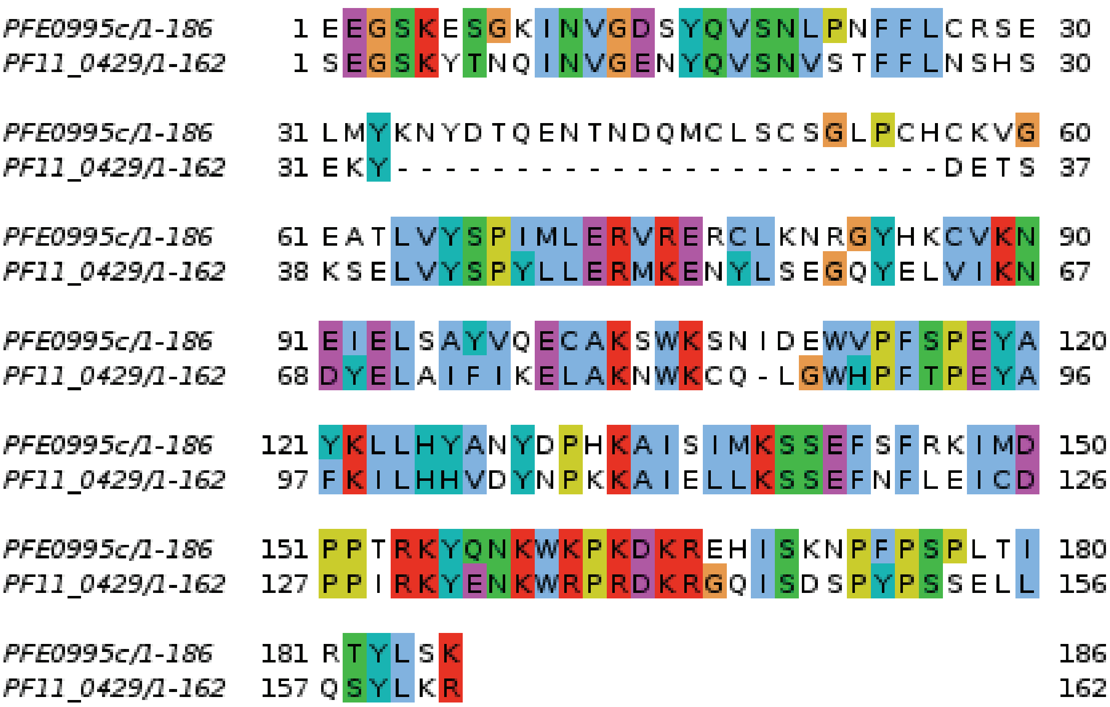

Extended ELM2 domain alignment graphic. A pairwise sequence alignment of the P. falciparum proteins encoding the domain is shown in graphical format. The raw data used to generate this graphic and the location of the domain in each protein's sequence can be found in Additional file 22.

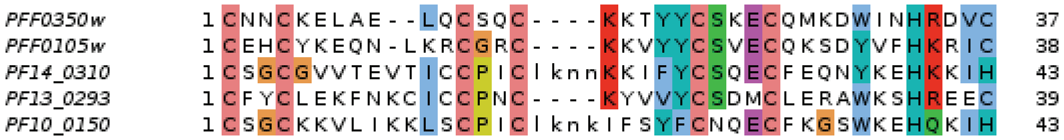

MYND domain alignment graphic. A multiple sequence alignment of P. falciparum proteins encoding the domain is shown in graphical format. The raw data used to generate this graphic and the location of the domain in each protein's sequence can be found in Additional file 23.
