# Supplementary material for: Organellar proteomics reveals hundreds of novel nuclear proteins in the malaria parasite Plasmodium falciparum
Source: Genome Biol. 2012 Nov 26;13(11):R108. doi: 10.1186/gb-2012-13-11-r108 (PMC4053738; doi:10.1186/gb-2012-13-11-r108)
Supplement: Additional file 20 — Pairwise sequence alignment of the two putative FG-repeat proteins PFI0250c and PF14_0442. [file gb-2012-13-11-r108-S20.PDF]

Additional file 20_Oehring et al.

```
 Score = 62.0 bits (149),  Expect = 1e-12, Method: Compositional matrix adjust.
 Identities = 71/283 (26%), Positives = 115/283 (41%), Gaps = 38/283 (13%)


PFI0250c   218    NSIFGGLGTSTNQSTGGGLFGNTGATSQNKTGGIFGGLSSTNQASTSSTSMFGGLSSNQA  277
                  +S+FG    + +  +   LFG+T          +FG L   N+      S+FG  S+
PF14_0442  1328   DSLFGS-SINNDDKSKINLFGSTMNDGDKNKTNLFGSL---NKDDKDKPSIFGSPSNKDD  1383


PFI0250c   278    KPTNSLFGGLSS----------GATSNTGTQQSGNLFGSASGIGQSKTVGGIFGNLSSTN  327
                  K   ++FG  S+          G +SN   +    +FGS S     K    IFG  S +N
PF14_0442  1384   KDKATIFGSPSNKDDKDKATIFGFSSNKDDKDKAPIFGSPSN-KDDKDKATIFG--SPSN  1440


PFI0250c   328    QASTSSSNMFGGLSSNQAKPTSSLFGGLSS----------GTTTNTSTQQSGNLFGSATG  377
                  +     + +FG  S+   K  + +FG  S+          G+ +N   +    +FGS +
PF14_0442  1441   KDDKDKATIFGFSSNKDDKDKAPIFGFSSNKDDKDKAPIFGSPSNKDDKDKAPIFGSPSN  1500


PFI0250c   378    LGQNKTGGGIFGTLPSANQTSTTSSNMFGGLSTNQAKPTSSLFGGMSSGT-------TGI  430
                     +K    IFG+ PS N+     + +FG  S    K  +++FGG + G         GI
PF14_0442  1501   -KDDKDKTPIFGS-PS-NKDDKDKTPIFGSPSNKDDKDKTAIFGGSTFGNNKSPMFGQGI  1557


PFI0250c   431    TTNTTAQSGNLFGGTGTSQNKTGNLFGALPG-ANQTSTTSNIF  472
                         S  +FG T TS +   +      G  ++  T  N+F
PF14_0442  1558   LNKGDNVSVPVFGNTVTSTDNDKSKLKLFNGNKDKEETKENVF  1600
```

Additional file 20. FG-repeat alignment graphic. A pairwise alignment of the two putative FG-repeat proteins, PFI0250c (PfNUP100) and PF14_0442, is shown. The display is modified from the output of a bl2seq alignment between the two protein sequences. While the alignment of specific FG pairs is unlikely to be the evolutionarily-motivated true alignment because of the repetition in these proteins, the number of FG motifs and detection of both proteins in the nuclear proteome suggest that these proteins are functional FG-repeat proteins. Amino acid positions refer to the respective PlasmoDB version 7.0 protein sequences.
