# Supplementary material for: Organellar proteomics reveals hundreds of novel nuclear proteins in the malaria parasite Plasmodium falciparum
Source: Genome Biol. 2012 Nov 26;13(11):R108. doi: 10.1186/gb-2012-13-11-r108 (PMC4053738; doi:10.1186/gb-2012-13-11-r108)
Supplement: Additional file 21 — PCR and western blot analysis to confirm C-terminal tagging of endogenous PF14_0442 by 3' replacement. [file gb-2012-13-11-r108-S21.PDF]

Additional file 21_Oehring et al.

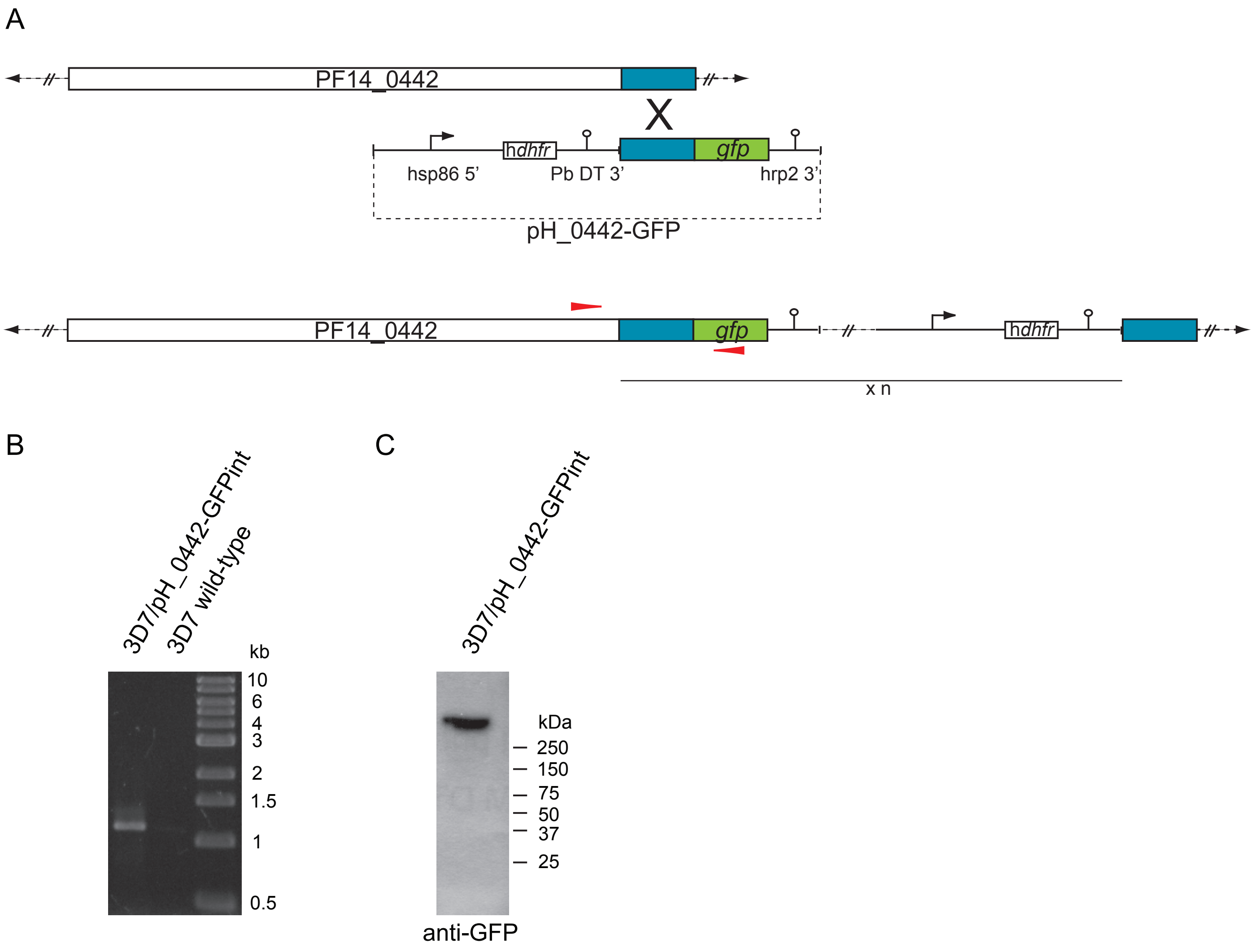

Additional file 21. C-terminal tagging of endogenous PF14_0442 by 3' replacement. (A) The schematic illustrates integration of pH_0442-GFP into the endogenous PF14_0442 locus by single cross-over recombination. Red arrowheads indicate the position of PCR primers used to verify the single cross-over event. (B) PCR using primers on gDNA isolated from 3D7/pH0442_GFPint and 3D7 wild-type parasites. (C) Western blot using anti-GFP antibodies on a nuclear extract derived from 3D7/pH0442-GFPint parasites.
