# Supplementary material for: Organellar proteomics reveals hundreds of novel nuclear proteins in the malaria parasite Plasmodium falciparum
Source: Genome Biol. 2012 Nov 26;13(11):R108. doi: 10.1186/gb-2012-13-11-r108 (PMC4053738; doi:10.1186/gb-2012-13-11-r108)
Supplement: Additional file 22 — cNLSs prediction in P. falciparum nuclear proteins. [file gb-2012-13-11-r108-S22.PDF]

Additional file 22. Prediction of cNLSs using three different prediction algorithms. Column A: set of proteins tested. Column B. Prediction algorithm used. Column C: Number of proteins with predicted NLS. Column D: number of proteins lacking a predicted NLS. Column E: percentage of proteins with predicted NLS in each set. Column F: p-value.

Protein set	NLS predictor	Positive predictions	Negative predictions	% positive	P-value (one sided Fisher's exact test; relative to all P. falciparum proteins)
All P. falciparum proteins	NLStradamus	2449	3042	45%	
	PredictNLS	1713	3778	31%	
	cNLS predictor	904	4548	17%	
Core nuclear proteome: 1 or more peptides	NLStradamus	409	386	51%	0.00828
	PredictNLS	296	499	37%	0.00647
	cNLS predictor	170	617	22%	0.00612
Core nuclear proteome: 2 or more peptides	NLStradamus	298	275	52%	0.007665
	PredictNLS	204	368	36%	0.0585
	cNLS predictor	121	445	21%	0.02433
Core nuclear proteome: nuclear fractions only	NLStradamus	238	185	56%	0.0004767
	PredictNLS	190	233	45%	0.0000263
	cNLS predictor	109	309	26%	0.0004754
