# Supplementary material for: Organellar proteomics reveals hundreds of novel nuclear proteins in the malaria parasite Plasmodium falciparum
Source: Genome Biol. 2012 Nov 26;13(11):R108. doi: 10.1186/gb-2012-13-11-r108 (PMC4053738; doi:10.1186/gb-2012-13-11-r108)
Supplement: Additional file 23 — mRNA expression profiles and maximal hour of mRNA expression of proteins found stage-specifically in the nuclear proteome. [file gb-2012-13-11-r108-S23.PDF]

Additional file 23_Oehring et al.

(a) (b)

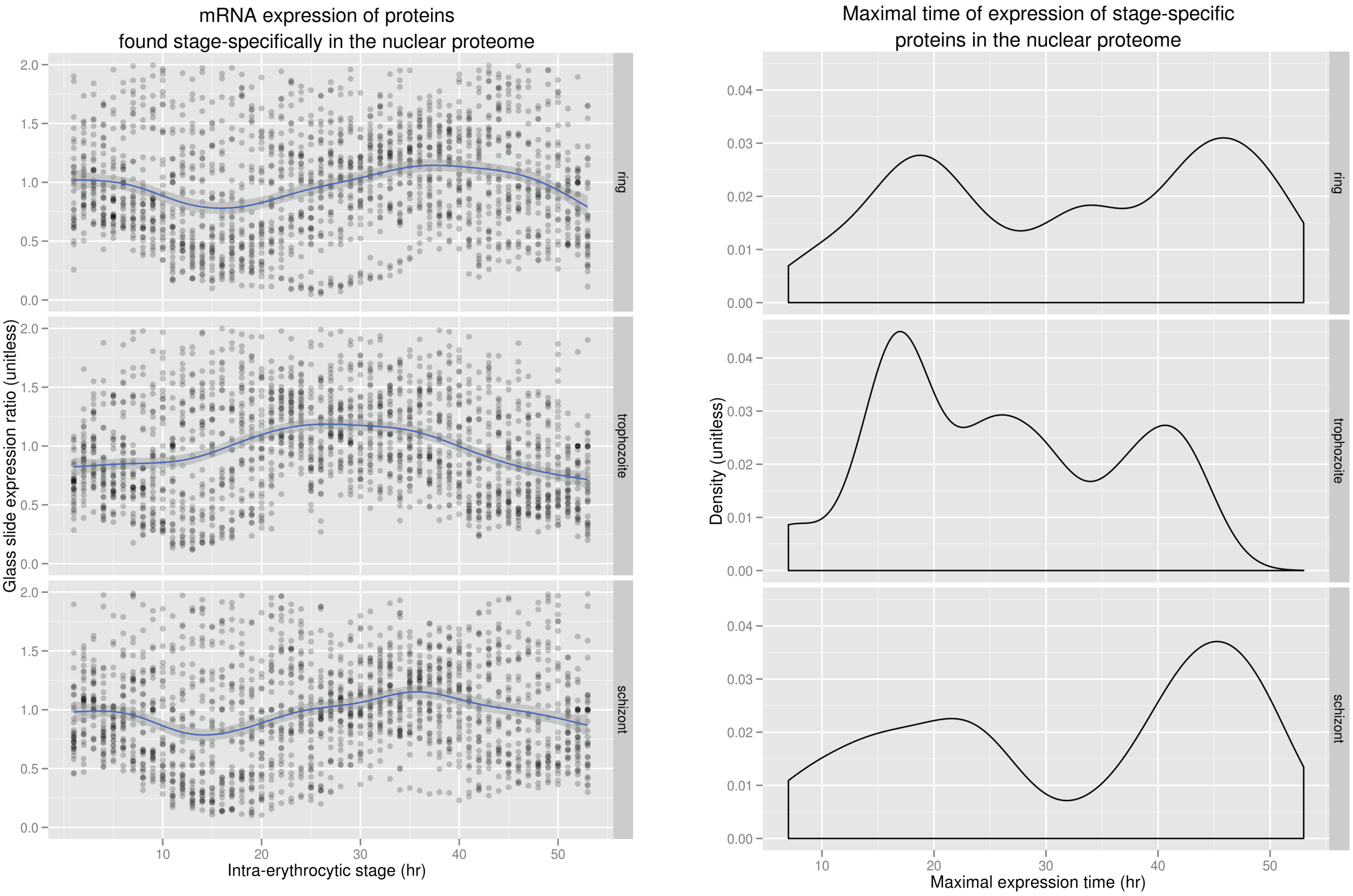

Additional file 23. (a) mRNA expression profiles of proteins found stage-specifically in the nuclear proteome. Each dot represents the expression profile of a stage-specific protein at a particular hour post-invasion during the P. falciparum 3D7 IDC. The x-axis refers to the hour during the IDC post-invasion. The y-axis represents relative expression of the transcript at that timepoint compared to an average over all timepoints [1]. Each protein was assigned a single dot in each timepoint, where that data was available. The dots are semi-transparent such that five overlayed dots appear as a olid black, and a single dot one fifth as dark. A minority of dots had expression ratios greater than two, and hence are not shown in the plot. (b) Maximal hour of mRNA expression of stage-specific proteins in the nuclear proteome.
First, the single hour at which maximal mRNA expression occured [1] was tallied for each protein found stage-specifically in the core nuclear proteome. Then these hours of maximal expression were put through a density estimation filter at 0.6 times the default of the density function in R, since this value appeared to be a good balance between being too noisy and being too board as to not see any patterns. Hence, a higher density represents a larger number of proteins being maximally expressed during that time period.

1. Llinas M, Bozdech Z, Wong ED, Adai AT, DeRisi JL: Comparative whole genome transcriptome analysis of three Plasmodium falciparum strains. Nucleic Acids Res 2006, 34: 1166-1173.
