# Supplementary material for: Organellar proteomics reveals hundreds of novel nuclear proteins in the malaria parasite Plasmodium falciparum
Source: Genome Biol. 2012 Nov 26;13(11):R108. doi: 10.1186/gb-2012-13-11-r108 (PMC4053738; doi:10.1186/gb-2012-13-11-r108)
Supplement: Additional file 26 — List of all primers used in this study. [file gb-2012-13-11-r108-S26.PDF]

Additional file 26. Primer sequences used in this study (restriction sites are in bold)

NuProc1 F	CAGT**GGATCC**TAAAATGAAAAAATTAAAAAATACAC
NuProc1 R	CAGT**GCTAGC**TTCTCTTTTTAAATTATCCTTTTTGG
NuProc2 F	GATC**GGATCC**TTAATATGGATGAACTAAATAAAG
NuProc2 R	GATC**CCATGG**TGTGTTGAATTTATTTTTTTTAAC
NuProc3 F	GATC**GGATCC**AAAAAATGTCCAATACATTAATTTC
NuProc3 R	GATC**CCATGG**TATTAACATCTGTTCAATTAAATC
NuProc4 F	CAGT**GGATCC**TTATCAATGGATCACCAAGATCTG
NuProc4 R	CAGT**CCATGG**TTTTTTCTTGGGTGAAGGTAAATTG
NuProc5 F	GATC**GGATCC**TTAAAATGAACACCGAAGAAAAAATTAATAC
NuProc5 R	GATC**CCATGG**ATCAGCTTGGCAAGTATCG
NuProc6 F	GATC**GGATCC**TAAATATGAATGATTATAATAATATAAATAATC
NuProc6 R	GATC**GC TAGC**TATTTTAGCGTCATAACCTTCG
NuProc7 F	CAGT**GGATCC**AAAAATGGGAGATAATAACACATCC
NuProc7 R	CAGT**CCATGG**TTTATCATCATCTTCTTCCTC
NuProc8 F	GATC**GGATCC**TAAAAATGTCGTCAGCAACAC
NuProc8 R	GATC**CCATGG**AAATTTTTTGTTTGAATCAAAAAC
NuProc9 F	GATC**GGATCC** TAC TTA TGG GTA GAA AGA AG
NuProc9 R	GATC**GCTAGC**TTCACAATTTTGTGTATG
NuProc10 F	CAGT**GGATCC**TGACCAATGATGCTTGAAG
NuProc10 R	CAGT**CCATGG**ATCTTTTTTTGAACTATCCTC
NuProc11 F	CAGT**GGATCC**AAAATATGGCTGATAATAACCTAG
NuProc11 R	CAGT**CCATGG**GTAAGAATCCTTTGATGAATCG
NuProc12 F	CAGT**GGATCC**AAAAATGAGTAATTACCAAAATTTGAAG
NuProc12 R	CAGT**CCATGG**TTGTGTTATCCCAAATCTAAGAG
NuProc13 F	CAGT**GGATCC**TTTAATATGGCTAATACATGGAGATG
NuProc13 R	CAGT**CCATGG**TGTTTTGTAATTAATTTTTTTTTTTGTAC
NuProc14 F	GATC**GGATCC**AAAAAATGAATGTTGAAGACATAAC
NuProc14 R	GATC**CCATGG**ATCCCATTGTTTGCTTCC
NuProc15 F	GATC**GGATCC**AATAAATGACCGATATACAAATAC
NuProc15 R	GATC**CCATGG**CTTTTTGACAAGATATGCTGC
NuProc16 F	CAGT**AGATCT**TAAATAATGAAGGATCCATTAG
NuProc16 R	CAGT**CCATGG**ACTTTCAGTATTGAAGTTATAT
NuProc17 F	GATC**GGATCC**ATAATATGTCTATAAGAAATAG
NuProc17 R	GATC**CCATGG**GAATCTACCATTTTTTACATTTTG
NuProc18 F	GATC**GGATCC**TAAATATGGGTACCTTTATAAAC
NuProc18 R	GATC**CCATGG**CATAAAATCCTCTAAATTATC
NuProc19 F	CAGT**GGATCCA**AAAATGAGTAATTACCAAAATTTGAAG
NuProc19 R	CAGT**CCATGG**TTGTGTTATCCCAAATCTAAGAG
NuProc20 F	CAGT**GGATCC**CAAAATGTCTACAATTAGATGGG
NuProc20 R	CAGT**CCATGG**ATTTAGAGTGTCAAATTTATGTAAC
NuProc21 F	CAGT**AGATCT**TAAAATGGCAGCTAATGAAGG
NuProc21 R	CAGT**CCATGG**AGAAGTAAGAGGATCCTCATTGC
NuProc22 F	GATC**GGATCC**AAAATATGCAAAAGAAGCCATC
NuProc22 R	GATC**CCATG**GCATTTCGGCTATTTCTTTATTTG
n-NuProc1 F	CAGT**GGATCC**AAAGAATGAATGTTACAAATGTAG
n-NuProc1 R	CAGT**CCATGG**CAATTCATCATCATATTGCTC
n-NuProc2 F	GATC**GGA TCC** TTA TGA TGA AGG TGT GGG G
n-NuProc2 R	GATC**CCATGG**TGAAAATATTTCTTCATAATTTG
n-NuProc3 F	GATC**GGATCC**TGAAAATGAGTCAACCACAAAAAC
n-NuProc3 R	GATC**CCATGG**TTTTGCGTTCTGTAAACTGGC
n-NuProc4 F	GATC**GGATCC**AAAAAATGAAGCTCAAATTAAATTTAG
n-NuProc4 R	GATC**CCATGG**TTTATTTTTTTTGCCAACAAC
n-NuProc5 F	GATC**GGATCC**AAAATGTTGGGTTTAATATTTAAAG
n-NuProc5 R	GATC**CCATGG**TAATGTCTTTAATTTCTTTACAATTTTG
n-NuProc6 F	CAGT**GGATCC**GTAATTATGAAGGGTAAAGGTAG
n-NuProc6 R	CAGT**CCATGG**TGATGTGTTATCTGGTGGC
0442 3′repl. F	GATC**CTGCAG**ATAAACGTATTTCCACATGC
0442 3′repl. R	CATG**GCGGCCGC**TATTTATCATATTTTGATTC
0442 int. F	TTAATGGATGTAATAAAAGTGATGATAGC
0442 int. R	ACCTTCAAACTTGACTTCAGCACGTGTCTTGTAGT
